# Mission Overview and Scientific Contributions from the Mars Science Laboratory Curiosity Rover After Eight Years of Surface Operations

**DOI:** 10.1007/s11214-022-00882-7

**Published:** 2022-04-05

**Authors:** Ashwin R. Vasavada

**Affiliations:** Jet Propulsion Laboratory, California Institute of Technology, Pasadena, CA USA

**Keywords:** Mars, Astrobiology, Geology, Meteorology, Climate, Planets

## Abstract

NASA’s Mars Science Laboratory mission, with its Curiosity rover, has been exploring Gale crater (5.4° S, 137.8° E) since 2012 with the goal of assessing the potential of Mars to support life. The mission has compiled compelling evidence that the crater basin accumulated sediment transported by marginal rivers into lakes that likely persisted for millions of years approximately 3.6 Ga ago in the early Hesperian. Geochemical and mineralogical assessments indicate that environmental conditions within this timeframe would have been suitable for sustaining life, if it ever were present. Fluids simultaneously circulated in the subsurface and likely existed through the dry phases of lake bed exposure and aeolian deposition, conceivably creating a continuously habitable subsurface environment that persisted to less than 3 Ga in the early Amazonian. A diversity of organic molecules has been preserved, though degraded, with evidence for more complex precursors. Solid samples show highly variable isotopic abundances of sulfur, chlorine, and carbon. In situ studies of modern wind-driven sediment transport and multiple large and active aeolian deposits have led to advances in understanding bedform development and the initiation of saltation. Investigation of the modern atmosphere and environment has improved constraints on the timing and magnitude of atmospheric loss, revealed the presence of methane and the crater’s influence on local meteorology, and provided measurements of high-energy radiation at Mars’ surface in preparation for future crewed missions. Rover systems and science instruments remain capable of addressing all key scientific objectives. Emphases on advance planning, flexibility, operations support work, and team culture have allowed the mission team to maintain a high level of productivity in spite of declining rover power and funding.

**Supplementary Information:**

The online version contains supplementary material available at 10.1007/s11214-022-00882-7.

## Introduction and Mission Science Strategy

### Introduction

This paper provides an overview of NASA’s Mars Science Laboratory (MSL) mission and the contributions of its international science team from its launch in late 2011 through the eighth anniversary of its landing in Gale crater on August 5, 2020 (Sol 2844). It extends and updates the Sol 0-500 overview of Vasavada et al. (2014). The reader also is referred to Grotzinger et al. (2012) for background information about the mission, payload, and landing site, and Grotzinger et al. (2014, 2015b) and Rampe et al. (2020a) for additional summaries of key geological and geochemical findings.

The rover’s scientific payload is listed in Table 1. Through Sol 2844 the mission has collected hundreds of thousands of images, nearly one million spectral measurements from thousands of rock and soil targets, millions of environmental measurements, and has analyzed 33 samples of drilled rock or scooped soil, leading to more than 700 peer-reviewed publications by team and external authors. MSL data are archived in NASA’s planetary data system (https://pds-geosciences.wustl.edu/missions/msl/index.htm).

**Table 1:** Scientific instruments onboard the Curiosity rover

Instrument name	Instrument description	Reference
*Remote science:*		
Mastcam (Mast Camera)	Multispectral science cameras	Malin et al. (2017)
ChemCam (Chemistry and Camera)	Laser-induced breakdown spectrometer and camera	Wiens et al. (2012), Maurice et al. (2012)
MARDI (Mars Descent Imager)	Nadir-viewing science camera	Malin et al. (2017)
*Contact science (arm-mounted):*		
MAHLI (Mars Hand Lens Imager)	Science camera	Edgett et al. (2012)
APXS (Alpha Particle X-Ray Spectrometer)	X-ray spectrometer	Gellert et al. (2015)
*Analytical laboratories:*		
CheMin (Chemistry and Mineralogy)	X-ray diffractometer	Blake et al. (2012)
SAM (Sample Analysis at Mars)	Mass spectrometer, gas chromatograph, tunable laser spectrometer suite	Mahaffy et al. (2012)
*Environmental monitoring:*		
DAN (Dynamic Albedo of Neutrons)	Active neutron spectrometer	Mitrofanov et al. (2012)
RAD (Radiation Assessment Detector)	High-energy radiation detector	Hassler et al. (2012)
REMS (Rover Environmental Monitoring Station)	Meteorology station	Gómez-Elvira et al. (2012)

The mission’s science objectives and strategy are recapped below. Sections 2–7 summarize scientific results across a number of disciplines. Sections 8 and 9 cover the performance of the rover, instruments, and sampling system. Section 10 discusses mission operations, overall performance, and management principles, and Sect. 11 describes future plans. An extensive narrative covering launch, cruise, landing, and surface operations is included in Online Resource 1 and provides context for the mission’s archived data sets, including maps with place-names. Online Resources 2 and 3 compare the mission’s performance with NASA-formulated requirements, and present a table of rover drive distances and parking coordinates, respectively.

### Overall Science Objectives and Strategy

The MSL science team uses Curiosity as a virtual field geologist and mobile geochemical laboratory to identify and quantitatively assess potentially habitable ancient environments. A habitable environment is one that has persistent liquid water, key chemical elements to build cellular structures and provide nutrients, plus sources of energy to fuel metabolism—in other words, the essential ingredients for life as we know it on Earth (National Research Council 2011). Habitability can be limited by low water activity or extremes of temperature, pH, salinity, or radiation. Insights into early martian surface environments and their evolution are valuable byproducts of a habitability investigation (Grotzinger et al. 2012). The mission accomplishes its NASA-derived science objectives (Online Resource 2) by integrating a range of measurements acquired in multiple geologic settings. Investigations are organized along three themes:

1. *Geologic Context:* Geological, chemical, mineralogical, and isotopic measurements reveal primary depositional processes, subsequent physical and chemical changes, geochemical pathways, and the time-ordered sequence of changing environments, including the presence and duration of liquid water.
2. *Habitability and Preservation of Organic Molecules:* Chemical and mineralogical measurements catalog chemical “building blocks of life” and constrain environmental parameters relevant to habitability, such as the water/rock ratio, pH, redox potential, salinity, and temperature during deposition and diagenesis. Energy sources for life are assessed through chemical and mineralogical diversity, particularly oxidation state. Of significance both to MSL and future Mars missions seeking signs of life is whether the environments recorded in the rock favored the concentration and preservation of organic molecules. Relevant measurements include the inventory of organic molecules (up to 535 atomic mass units), the physical and chemical environments during primary deposition, the nature and history of diagenesis, and the rate and timing of ionizing radiation.
3. *Evolution of the Atmosphere and Climate:* Continual measurements as the rover ascends through progressively younger strata reveal trends in the ancient climate from the nature of transitions between major rock packages, the presence of soluble/hydrated minerals, and the characteristics of aqueous environments. Isotopic analyses can detect the effects of ancient processes in the modern atmosphere and in the volatiles evolved from ancient rocks. Investigations of atmospheric composition, meteorology, and climatology, as well as monitoring of the surface radiation environment, reveal key processes and temporal variability.

Hypotheses in each of these areas are developed in advance and tested as the rover’s strategically planned route takes it across fundamental geologic contacts and into distinct ancient environments. The hypotheses, regional context, and route derive from geological mapping performed by the team using orbiter data sets (e.g., Grotzinger et al. 2014). When the rover arrives at a region of interest, the team builds a geological and geochemical framework from the rover’s observations, seeks to identify the environments and processes that led to the textures, chemical compositions, and mineralogic assemblages encountered, and assesses the potential for habitability and preservation of organic molecules.

## Geology and Paleoenvironment of Aeolis Mons (Informally Mount Sharp) and Aeolis Palus (Surrounding Plains)

**Fig. 1:**
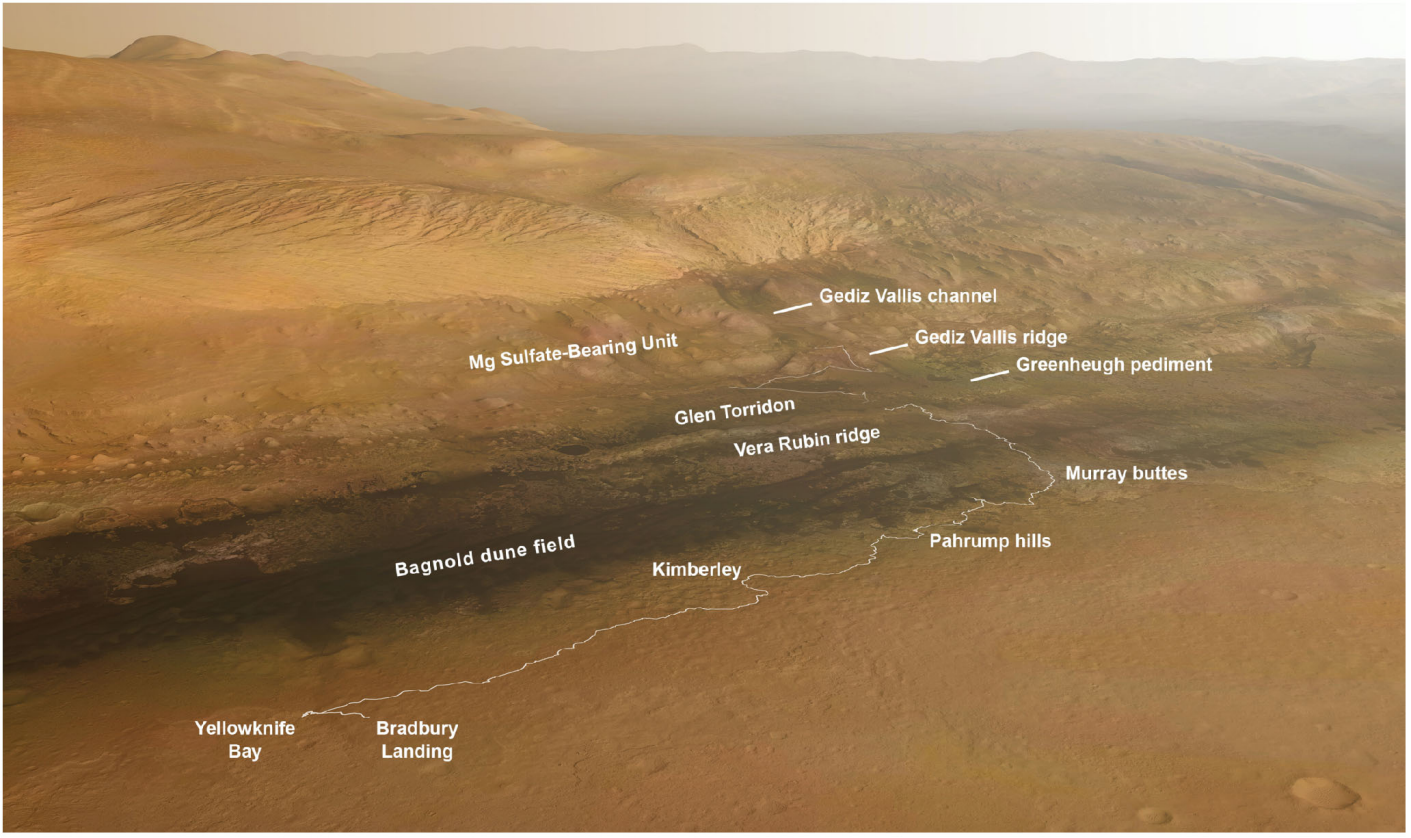
South-facing oblique view of Curiosity’s traverse annotated with geographic markers and morphological/mineralogical units as defined from orbiter data. The Vera Rubin ridge and Glen Torridon are coincident with the orbital hematite-bearing and clay-bearing units, respectively. Curiosity landed on the plains surrounding Mount Sharp and crossed onto the mountain at Pahrump hills. After initially traveling southwest to Murray buttes to reach a point where the Bagnold dunes could be safely crossed, the rover headed southeast and more directly uphill. The future strategic route is shown beyond Glen Torridon, where the rover is at Sol 2844 (background image credit: Seán Doran)

Mount Sharp in Gale crater provides the opportunity to investigate a thick sequence of sedimentary strata that formed within a span of several hundred million years starting about 3.6 billion years ago, and that may have recorded a major climate transition from wetter to drier conditions (Milliken et al. 2010; Thomson et al. 2011; Le Deit et al. 2013; Ehlmann and Edwards 2014). Prior to Gale crater’s selection as Curiosity’s landing site, investigators recognized that the strata vary in texture and mineralogy (Fig. 1), and include evidence for ancient aqueous environments of significance to habitability (Grotzinger et al. 2012).

The MSL science team has used rover observations to assemble the first continuous, high-resolution geological record of ancient Mars spanning ∼400 meters of stratigraphy, representing millions, perhaps tens of millions, of years of deposition. The record reveals that successive strata were deposited in a range of environmental conditions, though mostly in lacustrine settings. Trends in texture, geochemistry, and mineralogy further indicate that the sediments experienced multiple episodes of physical and chemical changes over an extended time period involving varied subsurface fluids, resulting in an overprinting similar to Earth’s earliest rock records. Surface materials investigated by Curiosity are dominantly clastic sedimentary rocks comprising basaltic igneous minerals, secondary phases including hydrated phases, and X-ray amorphous materials. With some exceptions, their compositions can be understood with processes generally involving fluids with moderate pH and low salinity, cool surface temperatures, and cool to warm burial temperatures, suggesting environments favorable for habitability (Sect. 3).

**Fig. 2:**
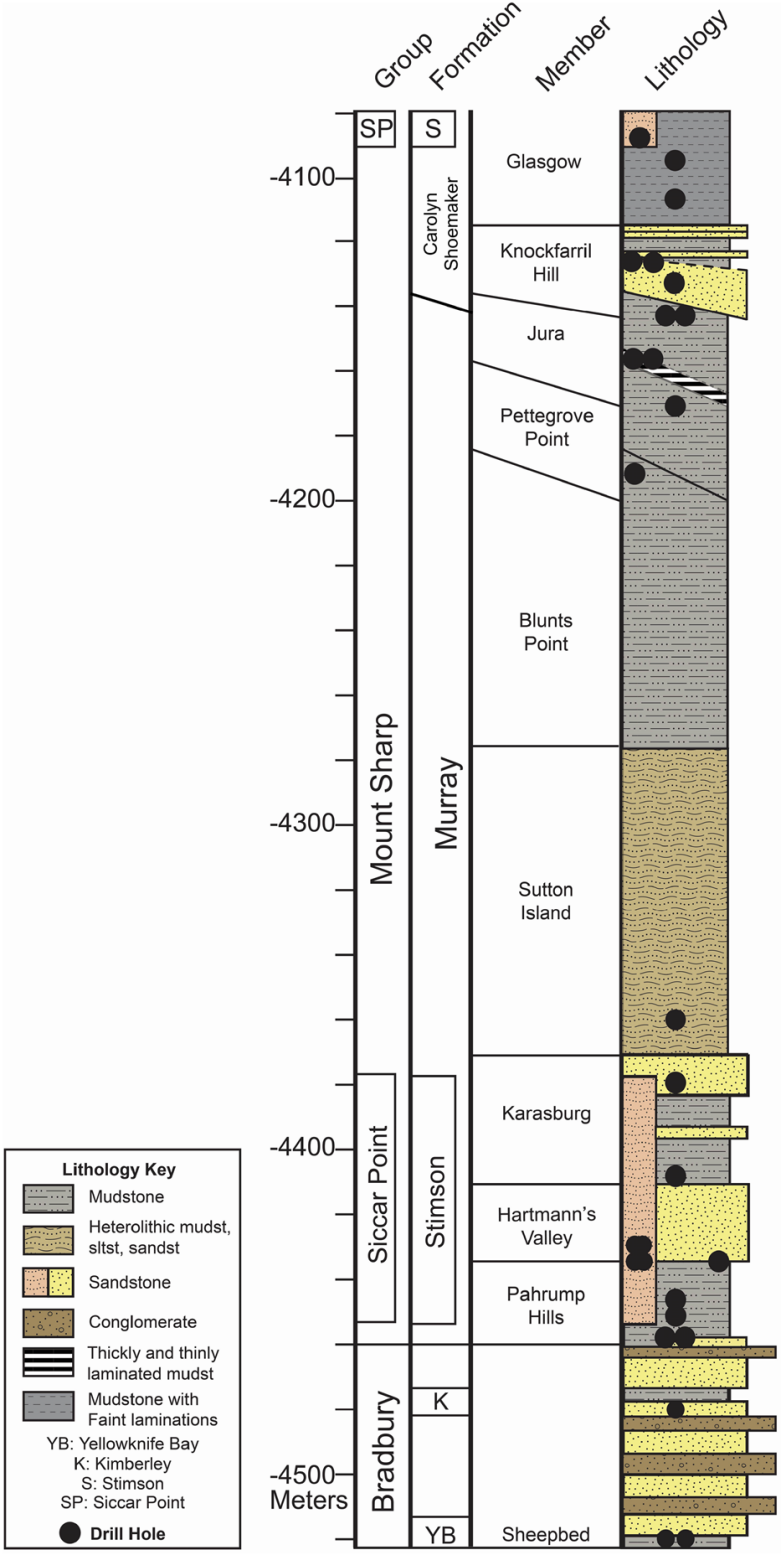
Cumulative stratigraphic column showing the vertical ordering of lithologic units encountered along Curiosity’s traverse. This two-dimensional representation was compiled from rover observations as it traversed both vertically and laterally. The width of segments in the lithology column indicates apparent resistance to erosion

The relative ordering of lithologic units encountered along Curiosity’s traverse is depicted in a cumulative stratigraphic column (Fig. 2). In situ measurements indicate that strata within the Mount Sharp group are flat-lying or nearly so (Stein et al. 2020), while mapping from orbit suggests major stratigraphic units persist laterally for kilometers (at least) around the rover’s path (e.g., Milliken et al. 2010; Fraeman et al. 2016; Edgar et al. 2020). The following sections describe the bedrock stratigraphy of the Bradbury, Mount Sharp, and Siccar Point groups, as well as the geochemical variability in the bedrock and in clasts that were found to have distinct textures and compositions. Investigations of unlithified sand deposits are covered in Sect. 4.

### The Bradbury Group on Aeolis Palus

The Bradbury group was investigated on the plains northwest of Mount Sharp along a ∼9 km traverse that spanned ∼65 m of elevation. It includes sandstone and cross-stratified pebbly sandstone with interbedded conglomerate, minor exposures of laminated mudstone such as at Yellowknife Bay, and rare aeolian strata, as summarized in Vasavada et al. (2014). Near the landing site, bedrock exposures are scattered as the undulating plains are covered with loose materials. Farther south into the crater basin, the terrain near the traverse transitions to a more rugged slope and ridge expression.

Curiosity’s observations of the Bradbury group support a hypothesis that sand and gravel were eroded from Gale crater’s northern rim and carried by streams into the basin, where they eventually encountered bodies of standing water. Cross-stratified sandstones, pebbly sandstones with grain-supported textures, and conglomerates with coarse sand and pebble-sized clasts are interpreted as evidence for bedload sediment transport and deposition in fluvial systems. The presence of subrounded to rounded pebbles suggests abrasion in flows that extended several km (Williams et al. 2013; Szabó et al. 2015). South-dipping inclined bedsets observed in the Kimberley region are interpreted as prograding small deltas (Grotzinger et al. 2015b). Nearby escarpments expose clinoform sandstones and show complex interfingering of sandstone and conglomerate facies (Williams et al. 2018).

The Yellowknife Bay region (Grotzinger et al. 2014) was investigated because its bright, layered appearance and high thermal inertia observed from orbit suggested cemented sedimentary bedrock, and because its location distal to an alluvial fan originating at the crater’s rim prompted a hypothesis of lacustrine deposition. The rover’s drill was used to sample an exposure of the very-fine-grained and smectite-bearing Sheepbed member of the Yellowknife Bay formation (fm), subsequently interpreted as lacustrine mudstone. Because the bulk chemistry indicates very limited element mobility, the preferred conclusion is that the phyllosilicates formed via in situ alteration. There is little evidence of chemical weathering or alteration of the sediment prior to deposition and subsequent formation of the phyllosilicates, suggesting rapid erosion and deposition, and that weathering reactions may have been inhibited by cold temperatures.

Nodules, hollow nodules, and raised ridges within the Sheepbed member are early diagenetic features thought to arise from salinity changes and gas production in fluid-saturated sediment (Grotzinger et al. 2014; Siebach et al. 2014). Curiosity also found evidence throughout the Bradbury group of burial and late-stage diagenesis driven by subsurface fluids, such as well-cemented sandstones and calcium sulfate veins that cross-cut sedimentary units at Yellowknife Bay and the Kimberley (Nachon et al. 2014).

Strong enrichments in manganese have been detected occasionally across the traverse, including notably at the Kimberley region, where they are associated with fracture fills and consistent with the presence of manganese oxides (Lanza et al. 2016). Precipitation and concentration of manganese oxides in mineral veins would imply that strong oxidants were present in fluids that flowed through the fractures, but the source of the manganese, its mineral phase, and the nature of the oxidation are not known.

The areal extent of the Yellowknife Bay lake and its timing relative to the strata of Mount Sharp (Sect. 2.2) have not been determined. However, observations are consistent with the Sheepbed mudstone of the Yellowknife Bay fm being the stratigraphically lowest and therefore oldest unit examined by Curiosity (Caswell and Milliken 2017). Impact excavation of other parts of Aeolis Palus, as well as broader constraints imposed by Gale crater’s geometry, indicate that the present surface is underlain by hundreds to thousands of meters of sedimentary strata, potentially including additional lacustrine deposits (Grotzinger et al. 2015b; Buz et al. 2017).

### The Mount Sharp Group

**Fig. 3:**
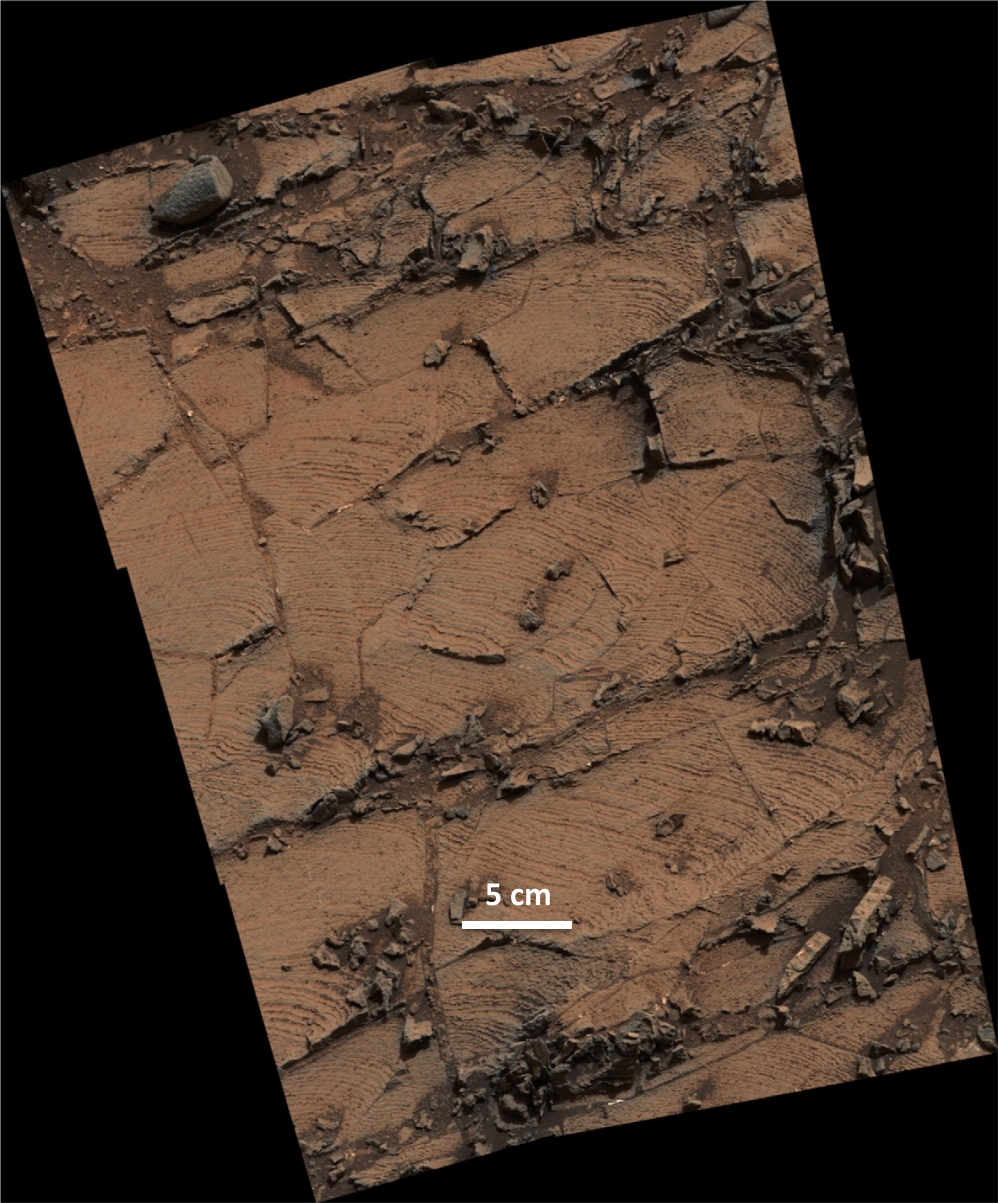
Mastcam mosaic of finely laminated mudstone near the Telegraph Peak drill site, Pahrump Hills, acquired on Sol 837 (MR003676, image credit: NASA/JPL-Caltech/MSSS)

The presence of outcrops interpreted as evidence of fluvial and deltaic environments on Aeolis Palus implied that as the rover traveled further down the depositional gradient into the basin now occupied by Mount Sharp, it would encounter rocks comprising the fine-grained sediment that accumulated near and within the lake (Grotzinger et al. 2015b). Investigation of ∼330 m of strata (to Sol 2844) at the base of Mount Sharp has borne this out. Rocks of the Bradbury group are interpreted to interfinger with those of the Mount Sharp group (e.g., Fig. 3 of Grotzinger et al. 2015b). The basal formation of the Mount Sharp group, the Murray fm, is characterized by three facies assemblages up through the Blunts Point member: mm-scale flat-laminated mudstone and siltstone (Fig. 3), meter-scale cross-stratified sandstone, and a mixed facies of mudstone along with sandstone that exhibits small-scale sedimentary structures indicative of transport by water and wind. The Murray fm strata are most often consistent with lacustrine deposition, but also contain intervals interpreted to record fluvial, fluvial-deltaic, marginal lacustrine, and aeolian environments (Stack et al. 2019; Fedo et al. 2019; Stein et al. 2018).

Rocks of the Mount Sharp group have a wide range of chemical and mineralogical compositions, consistent with sediment from multiple sediment sources, varying degrees of sorting during transport, and diagenesis (Siebach et al. 2017; Bedford et al. 2019; Rampe et al. 2020a; Achilles et al. 2020). Samples drilled from rocks of the Mount Sharp group show evidence for open-system weathering and chemical mobility (Hurowitz et al. 2017; Mangold et al. 2019; Rampe et al. 2020a). Phyllosilicates vary in abundance but are detected in most samples. They trend from trioctahedral toward dioctahedral smectite with elevation (Bristow et al. 2015, 2018), suggesting increasing oxidative weathering. Additional evidence for oxidation, chemical weathering, and diagenesis relative to lower elevations include higher hematite/magnetite ratios, greater abundance of calcium sulfate in the rock matrix, and minor amounts of magnesium and iron sulfates. Isolated high abundances (up to 25 wt.%) of halite are inferred from point measurements of sodium and chlorine on bedrock and at the margins of veins and nodules within several members of the Mount Sharp group. Its occurrence is thought to reflect remobilization and reprecipitation by diagenetic fluids (Thomas et al. 2019). Primary evaporitic sources have not yet been identified, but some sources may have been within the strata investigated by Curiosity. For example, desiccation features (Stein et al. 2018) and thin, sulfate-enriched beds (Rapin et al. 2019) are found in the Sutton Island member, which is characterized by a heterolithic (mudstone-sandstone) facies interpreted to record lake and lake margin environments (Fedo et al. 2019).

Stratigraphically above the Blunts Point member (Fig. 2), the Pettegrove Point member was encountered as the rover ascended the Vera Rubin ridge (VRR), a geomorphic feature associated with strong spectral absorptions consistent with red crystalline hematite (Fraeman et al. 2016). The overlying Jura member crops out both on the upper part of the ridge and within Glen Torridon (Fedo et al. 2020), a shallow trough to the south associated with strong spectral absorptions consistent with smectite clay minerals (Milliken et al. 2010). Curiosity traversed across the contacts between the Blunts Point, Pettegrove Point, and Jura members multiple times on the northern face of the ridge, providing a rare opportunity to assess the lateral continuity of the stratigraphy over a few hundred meters. The transects show a similar stratigraphic sequence, but with small shifts in elevation (Fig. 2) that may be explained by differential compaction or a lateral variation in facies (Edgar et al. 2020).

Both the Pettegrove Point and Jura members are fine-grained, thinly laminated, and interpreted to record deposition dominantly in a low-energy, lacustrine setting (Edgar et al. 2020). Given that neither hematite nor other iron oxides are significantly enriched on the ridge, the strong spectral absorptions likely arise from the effects of larger grain size and enhanced crystallization. Fraeman et al. (2020a) conclude that diagenesis in the presence of subsurface fluids is a cause of the spatially variable abundance, types, and crystallinity of iron oxides observed on the VRR.

Curiosity observed several compositional differences between time-equivalent outcrops of the Jura member on the ridge and in the Glen Torridon trough, most notably abundant hematite, minor akaganeite and jarosite, and a lack of clay minerals on the VRR (Rampe et al. 2020b). These differences suggest the presence of a diagenetic front coincident with the present location of the ridge, where silica-poor brines converted clays into iron oxides and oxyhydroxides (Bristow et al. 2021). Recrystallization of ferric iron oxides may have enhanced cementation at the ridge, demonstrating the importance of both depositional and post-depositional diagenetic processes in forming the present landscape.

The in situ exploration of Gale crater has shown that hydrated minerals and crystalline iron oxides are significantly more widespread than is apparent from orbit. Prior to Curiosity’s exploration, Vera Rubin ridge and Glen Torridon were described as the “hematite unit” and “clay-bearing unit”, respectively, based on interpretations of orbiter spectral data. Curiosity indeed found the highest abundance of each phase at the respective locations. However, hematite and phyllosilicates are present in nearly all samples drilled from the Mount Sharp group, even in areas where they are not detected from orbit. Obscuration by dust and sub-pixel mixing with sand likely inhibit their detection from orbit in certain areas within Gale (Fraeman et al. 2020b). Repeated measurements of the hydration of the bulk shallow subsurface with Curiosity’s Dynamic Albedo of Neutrons (DAN) neutron spectrometer track changes in the abundance of hydrated minerals and reveal the lateral extent of such exposures (Litvak et al. 2014). The implied water abundance of ∼4 wt.% from DAN hydrogen measurements in Glen Torridon extends into areas where reflectance spectroscopy is inhibited by dust and sand (Czarnecki et al. 2020a).

**Fig. 4:**
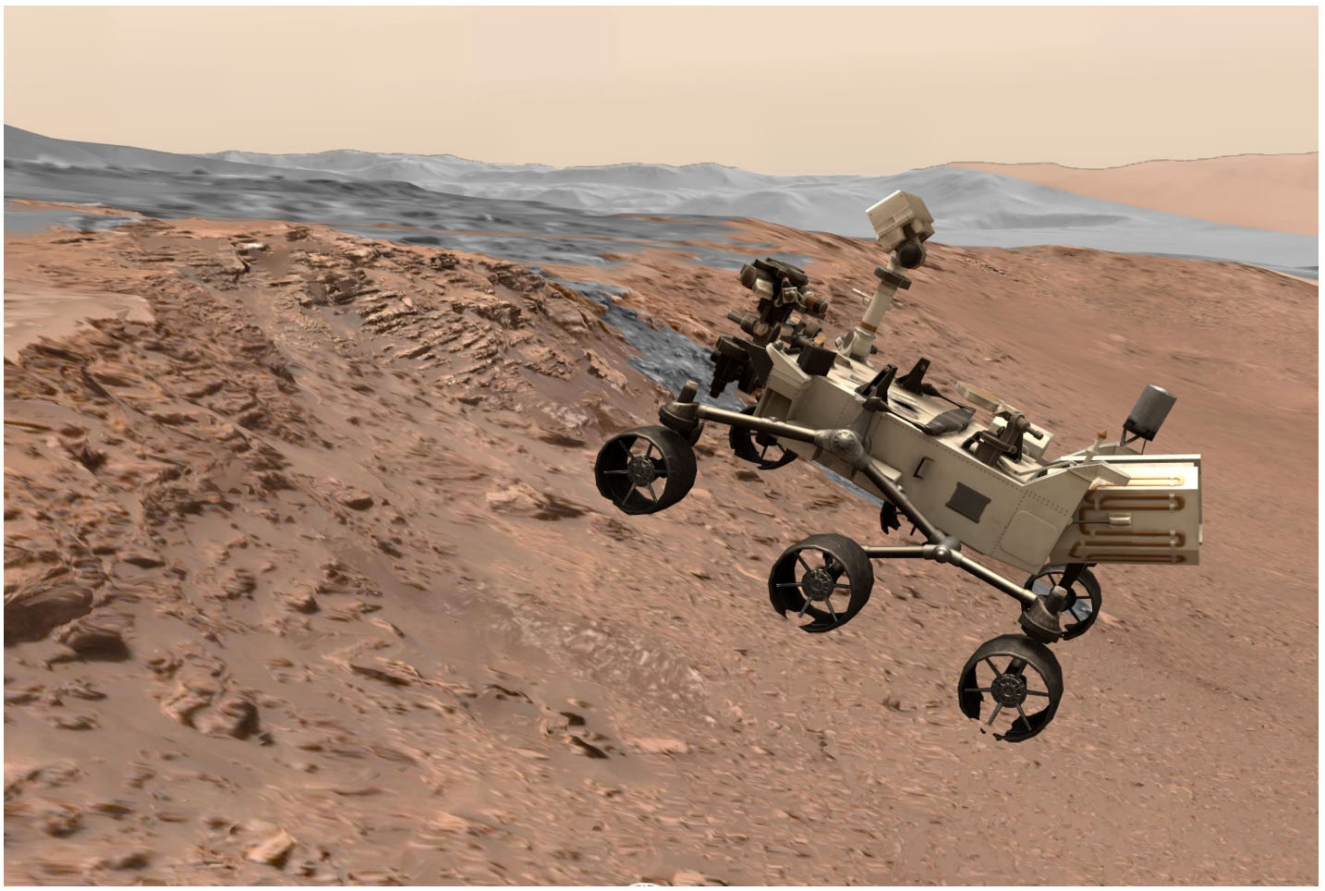
Rendering from the OnSight visualization tool developed by JPL and Microsoft based on the orbiter-derived digital elevation model and rover images and telemetry from Sols 2476-2477. The rover was parked at a near-record 25° tilt in order to acquire remote and contact science observations of bedrock exposed along a ridge in Glen Torridon

Exposure of the smectite-bearing Knockfarril Hill member, which overlies the Jura, is confined to ridgetops in the northern part of Glen Torridon (Fig. 4) but becomes continuous further south. This unit is characterized by a mix of silt and sand grain sizes and shallow trough cross-stratification, and is interpreted to have been deposited within higher-energy lake margin or fluvial environments (Fedo et al. 2020). The Glasgow member, characterized by finely laminated mudstone, was observed between the Knockfarril Hill member and the basal Siccar Point unconformity. The increased diversity of depositional environments within the Knockfarril Hill and Glasgow members diverges from the dominantly lacustrine underlying Murray formation strata, leading the team to place them within a new formation, Carolyn Shoemaker (Fig. 2).

**Fig. 5:**
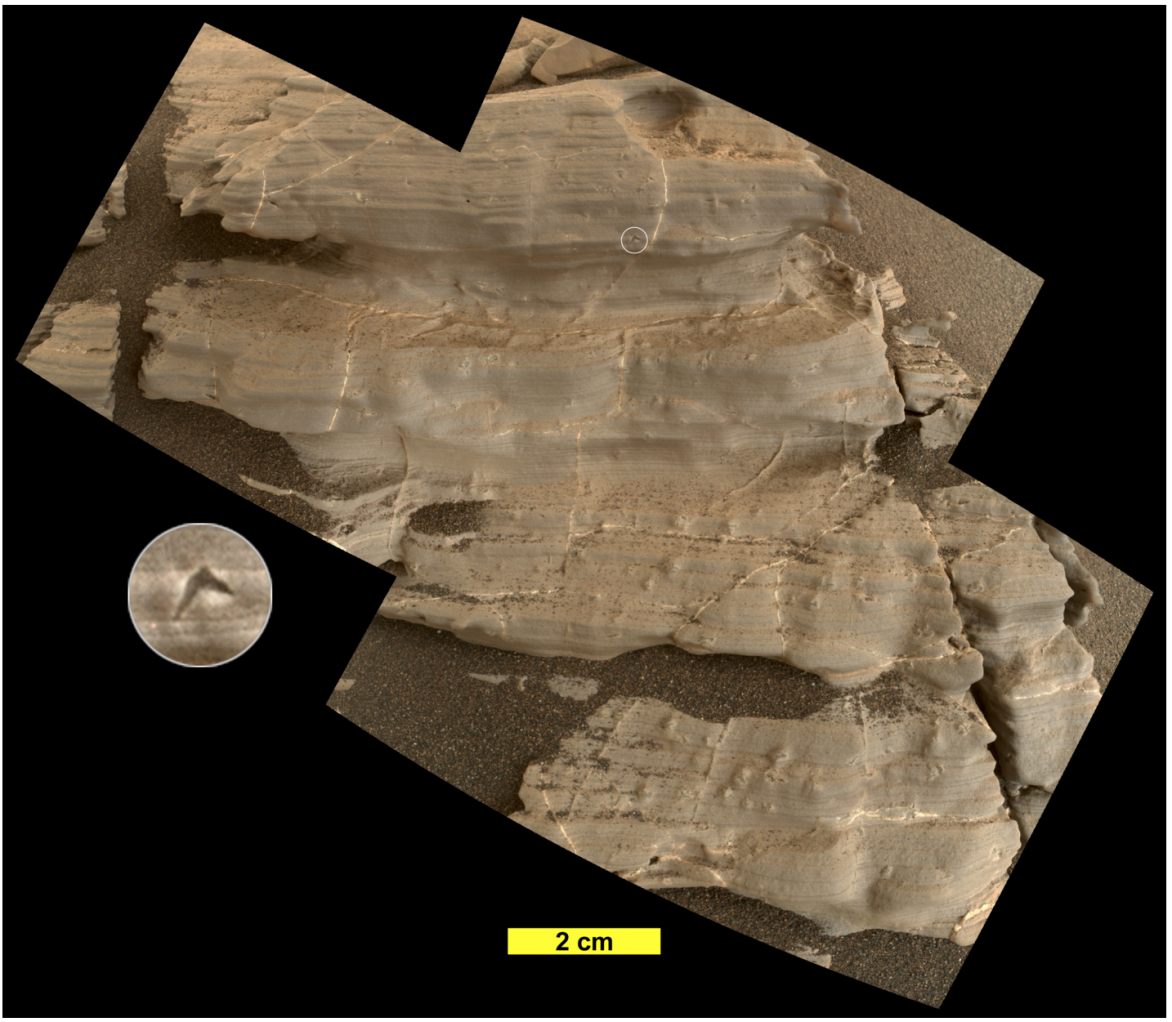
Example of diagenetic overprinting. This MAHLI mosaic from the “Jura” target on Sol 1925 shows a highly eroded fragment of finely laminated mudstone of the Jura member on Vera Rubin ridge. Fractures are filled with calcium sulfate veins. Millimeter-scale crystal forms have lenticular, “swallow tail,” and star forms that are characteristic of gypsum crystals. The inset (3 mm in diameter) is an example of the swallow-tail shape. Neither the veins nor crystal forms deform primary laminations and therefore are interpreted to have occurred post-lithification (image credit: NASA/JPL-Caltech/MSSS)

The diversity of fracture-filling veins and other diagenetic features observed in the Mount Sharp group (Fig. 5) is best explained by multiple generations and compositions of diagenetic fluids. Dendritic aggregates and other relief-enhanced features in the Pahrump Hills member have compositions consistent with magnesium sulfates and appear to have formed after sediment compaction, but before other episodes of diagenesis (Nachon et al. 2017). The widespread occurrence of veins and their cross-cutting relationships suggest that they originated from multiple episodes of hydrofracturing resulting from migration of over-pressured subsurface fluids (Nachon et al. 2014; Kronyak et al. 2019). White veins composed of variously hydrated calcium sulfate are observed throughout the Mount Sharp group. Less common, darker veins near Pahrump Hills are associated with enrichments in fluorine (Forni et al. 2016) and are temporally and compositionally distinct from each other and from the white veins (Kronyak et al. 2019). Most concretions appear to have formed post-cementation and lithification, at which point episodes of concretion and vein formation were intermingled or simultaneous (Sun et al. 2019).

### The Siccar Point Group: Aeolian Sandstones Draping Mount Sharp

The Siccar Point group consists of the eroded remnants of strata that drape the Mount Sharp group over hundreds of meters of elevation. It includes meter-scale cross-bedded sandstones and other capping strata that are observed to unconformably overlie the truncated Mount Sharp group strata (Fraeman et al. 2016; Banham et al. 2018, 2021a). Williams et al. (2018) propose that outcrops of Siccar Point group rocks also unconformably overlie the Bradbury group on Aeolis Palus.

The team closely examined the contact between the Murray fm of the Mount Sharp group and the meters-thick Stimson fm of the Siccar Point group at Marias Pass, documenting evidence for the ongoing erosion of the lithified paleosurface during the deposition of the Stimson (Newsom et al. 2016; Edgett et al. 2020). The rover traversed along the Stimson’s upper surface while on the Emerson and Naukluft plateaus, and observed isolated outcrops at the Murray buttes. Siccar Point group rocks were subsequently re-encountered only after the rover climbed an additional ∼200 m elevation and reached the Greenheugh pediment, a sloping surface just above the Glen Torridon region and capped by Siccar Point group sandstones.

Banham et al. (2018, 2021a, 2021b) interpret the Stimson fm to have been deposited by migrating aeolian dunes in an arid environment sometime after the sediments of the Mount Sharp group were buried, lithified, and exposed by erosion. They also find that the paleoclimate resulted in winds that transported sand to the northeast, in the opposite direction and with larger particle sizes than what is observed in the currently active Bagnold dune field (Sect. 4). An exception is the Greenheugh pediment capping unit, where both northward and southward winds are inferred.

Bedford et al. (2020) find evidence that wind-driven sorting decreased the proportion of felsic minerals from southwest to northeast in the Stimson fm exposures on the Emerson and Naukluft plateaus, as would be predicted from the inferred sediment transport direction, and opposite of the sorting and transport direction observed in the Bagnold dunes. The mineralogy is similar to that of loose sand in Gale, with the notable absence of olivine, implying moderate weathering if derived from a common source (Rampe et al. 2020a). However, similarity of the bulk geochemical compositions of the Stimson fm and Bradbury group led Bedford et al. (2020) to propose that the sediments within those units derive from erosion of the crater rim, while the loose sand may have a more distant source. The Greenheugh capping unit sandstone uniquely includes olivine and phyllosilicates, suggesting it may have a different provenance and/or diagenetic history than its northern counterparts (Bedford et al. 2021).

**Fig. 6:**
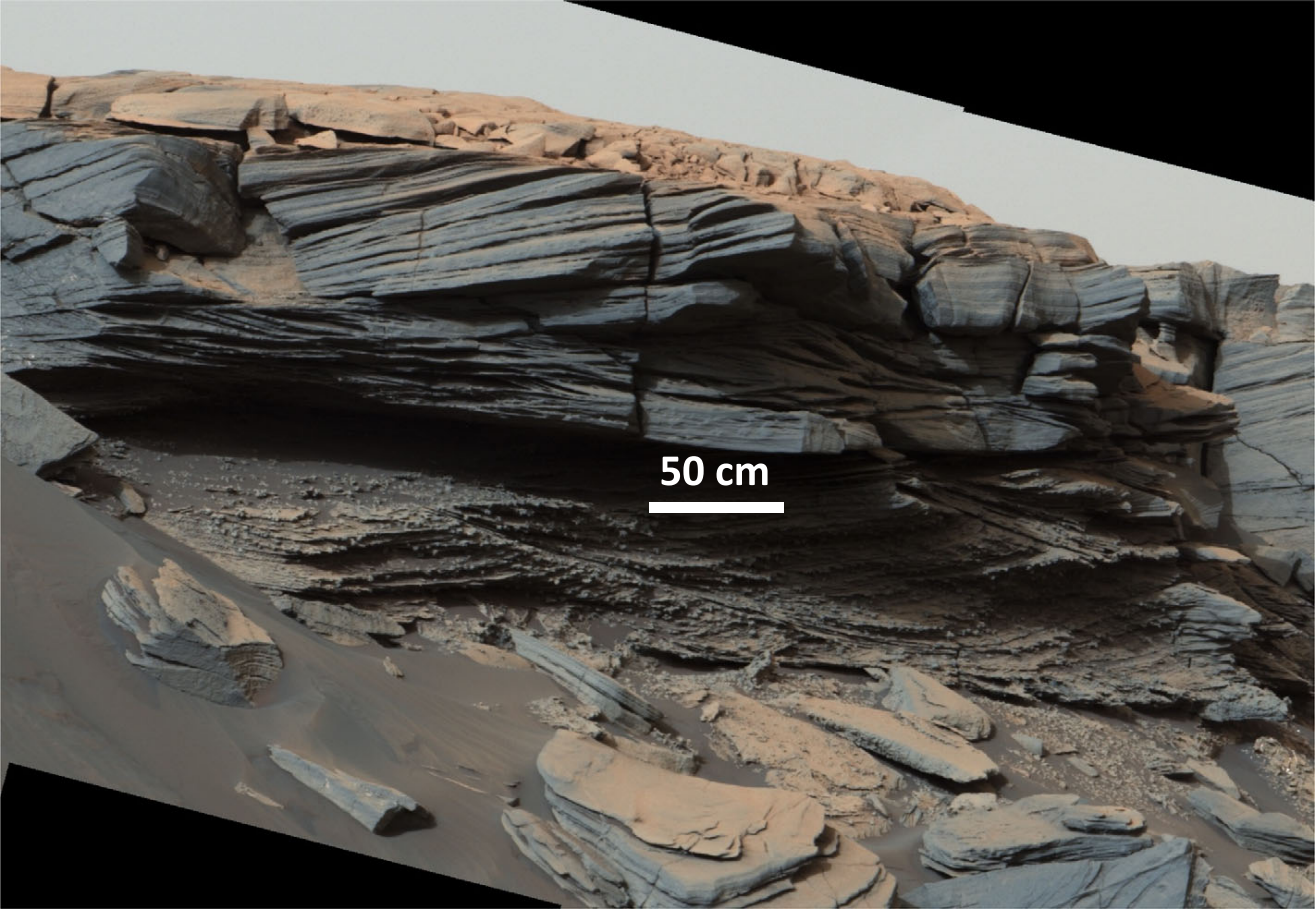
Mastcam mosaic of the basal Siccar Point group unconformity acquired on Sol 2685 showing crossbedding in the Greenheugh pediment capping unit and the nodular texture just above the contact with the underlying Mount Sharp group (MR014053, image credit: NASA/JPL-Caltech/MSSS)

There are multiple lines of evidence that the Stimson fm was subjected to diagenesis involving fluids, one of which is its lithification. In addition, a cross-bedded sandstone facies observed just above the basal unconformity of the Siccar Point group is rich in cm-scale concretions (Fig. 6) that are thought to arise from preferential cementation (Hurowitz et al. 2017; Banham et al. 2018). The Stimson also experienced two generations of post-lithification fractures and veins. The veins are filled with calcium sulfate. The fractures are unmineralized but are characterized by decimeter-scale fracture-associated alteration halos enriched in silica and depleted in the mafic crystalline phases found in the unaltered Stimson fm sandstone. Alteration is thought to have occurred in stages involving both neutral/alkaline and low-pH fluids (Frydenvang et al. 2017; Yen et al. 2017; Hausrath et al. 2018). These alteration halos are observed to cross the contact with the underlying Murray. Additional evidence of diagenesis in the Mount Sharp group related to the overlying unconformity includes lighter coloration, more extensive and prominent veining, and compositional changes (Frydenvang et al. 2017).

### Inferred Paleoenvironments and Paleoclimate

Curiosity’s observations lead to a model that describes the accumulation of sediment within Gale crater (Grotzinger et al. 2015b). The model posits that the lower levels of Mount Sharp were built by fluvio-lacustrine systems in multiple episodes over (at least) millions of years. The Bradbury and Mount Sharp groups are interpreted to be a record of persistent stream, delta, and lake environments arranged in a stacking pattern that moved forward into the basin over time as the crater infilled and the lake level rose. Subsequent erosion, presumably by wind (Day and Kocurek 2016), excavated material inward of the crater rim, giving Mount Sharp its present shape. Accordingly, the stream and lake deposits now exposed in the Bradbury and Mount Sharp groups are erosional remnants of superimposed depositional sequences that once extended laterally across and hundreds of meters above the current elevation of the crater floor. The model further implies that rocks encountered by the rover were once buried to such depths, consistent with observed fracturing that requires significant overburden (Caswell and Milliken 2017). Strictly speaking, the model applies only to the ∼0.4 km thickness of strata explored by the rover thus far, which is ∼5 km below the summit of Mount Sharp and 2 to 5 km below the present-day crater rim. Complete infilling of the crater is not assumed in the model. In fact, complete infilling is contradicted by some findings (Borlina et al. 2015) and would not be possible via fluvially transported sediment (Thomson et al. 2019). Upper units of Mount Sharp have been interpreted as aeolian (Anderson and Bell 2010).

The setting ∼3.6-3.2 billion years ago would have been similar to an “overfilled” lake basin on Earth (i.e., Bohacs et al. 2000), characterized by high sediment flux and an accumulation of water that exceeds loss from evaporation (Grotzinger et al. 2015b; Stack et al. 2019; Fedo et al. 2017). A comparison with depositional rates in Earth’s lacustrine settings indicates that lakes were present in Gale for at least millions of years (Grotzinger et al. 2015b; Stack et al. 2019). There is evidence for lake-level changes, intermittent dry periods, increasing salinity, and more intense chemical weathering in the upper part of the section (Fedo et al. 2017; Gasda et al. 2017; Stein et al. 2018; Bristow et al. 2018; Mangold et al. 2019; Thomas et al. 2019; Rapin et al. 2019). No unambiguous evaporite textures have been found.

The processes inferred to have occurred within Gale imply an ancient atmosphere and climate capable of providing sufficient pressure and humidity to allow fluvial erosion, sediment transport, and re-charging of the lakes with a hydrological cycle. Increased humidity would be needed within Gale to reduce evaporative loss from open water, while increased warmth and humidity on a regional (if not planetary) scale would be needed to reduce net loss of water to frozen regions via cold-trapping. How Mars developed and sustained such a climate, given the faint young Sun and the limited efficacy of greenhouse warming in current climate models, is unresolved and an active area of research (e.g., Wordsworth 2016). Groundwater influx likely helped to sustain Gale’s lakes (Baum and Wordsworth 2020; Horvath and Andrews-Hanna 2017; Roseborough et al. 2021).

Multiple lines of evidence argue for a prolonged history of water at and/or below the surface. Through Sol 2844 Curiosity had not encountered strata within the Mount Sharp group that indicate an end to the dominance of subaqueous deposition. Even after streams and lakes disappeared, water must have persisted in the subsurface in order to create the diagenetic overprints described above, most of which occurred post-lithification. Martin et al. (2017) derive a formation age of <3 billion years for diagenetic jarosite at the Mojave drill site at Pahrump Hills. This finding also implies that acidic fluids may have been part of the later history of water in Gale. The basal unconformity of the Siccar Point group represents a gap of unknown duration, during which a major environmental transition occurred that resulted in a change from net deposition to net erosion of the Mount Sharp group and emplacement of the dry dunes of the Stimson fm. Yet even after this gap, subsurface fluids were involved in lithification and diagenesis. Finally, multiple landforms that post-date the erosion of both Mount Sharp and Siccar Point group rocks are consistent with at least sporadic surficial water. These include a possible fluvial channel within Gediz Vallis on the flank of Mount Sharp and numerous deltas, gullies, and alluvial deposits around Gale crater’s rim and floor that are consistent with lakes that were up to hundreds of meters deep and persisted for at least thousands of years (Palucis et al. 2014, 2016). Such activity may have extended into the Amazonian (Grant and Wilson 2019).

### Additional Notes on Geochemistry, Mineralogy, and Provenance

The geochemical and mineralogical variability encountered by Curiosity suggests that sediments within the crater derive dominantly from basaltic crustal sources, but also reflect input from more evolved (e.g., alkali- or silica-enriched) sources. Sautter et al. (2015) first reported feldspar-rich rocks that reflect unexpected magmatic diversity in the vicinity of Gale crater. Bedford et al. (2019) identify five igneous endmember sources, including a subalkaline basalt, a trachybasalt, a potassium-rich volcanic source, a highly-evolved, silica-rich igneous source, and a fractionated, relatively silica-rich subalkaline basalt. They argue that variable contributions from these sources most strongly control the observed changes in bulk composition with stratigraphic height, with mineral sorting and chemical weathering playing significant and minor roles, respectively.

Cousin et al. (2017a) interpret several dozen clasts observed within Aeolis Palus as igneous and note the wide range of compositions. Basalts and gabbros are the most common overall, but felsic compositions are more abundant near alluvial materials. The latter may have been transported from the crater rim, although felsic materials have not been inferred from orbiter spectroscopy (Buz et al. 2017).

The presence of highly potassic sanidine in the sample drilled from the Kimberley fm suggests detrital input from an alkali-rich source (Treiman et al. 2016). Alternatively, Morris et al. (2020) prefer in-place alteration of plagioclase by hydrothermal fluids, although a geothermal source has not yet been identified within Gale. Volcanic rocks are notably absent along Curiosity’s traverse.

Some stratigraphic levels of the Murray are enriched in amorphous silica as opal-A and opal-CT, crystalline silica as cristobalite, and in the Buckskin sample (Marias pass) as ∼14 wt. % tridymite. DAN neutron spectroscopy is consistent with the high-silica layer extending to at least a meter below the rover near Buckskin (Czarnecki et al. 2020b). Tridymite is the low-pressure, high-temperature polymorph of SiO_2_ and is most commonly associated on Earth with high-silica volcanism (Morris et al. 2016). Its presence suggests that an unidentified silicic volcanic source region provided sediment to the lake. Silica also may have been precipitated from oversaturated groundwaters that entered the lake to create silica-rich primary sediment (Hurowitz et al. 2017).

Samples of sedimentary rock analyzed by Curiosity have consistently high fractions (average of ∼40% by weight) of X-ray amorphous and/or nanocrystalline material, with variable compositions dominated by silica, iron oxides, and sulfates (Rampe et al. 2020a; Smith et al. 2020; Achilles et al. 2021). Patterns in the abundance, composition, and geologic context suggest that the variability may have arisen as sediments accumulated a mixture of phases over time, both pre- and post-deposition, primarily via aqueous processes. It is possible that some of these amorphous materials are cements that bind the sedimentary particles together (Grotzinger et al. 2015a). These phases have persisted in the martian geological record far longer than in Earth’s.

Samples analyzed by Curiosity show a surprisingly large range of isotopic variability in carbon, sulfur, and chlorine, likely the result of fractionation processes in the crust and atmosphere, as well as mixing between these reservoirs (Franz et al. 2017b; Farley et al. 2016). Curiosity’s characterization of the host phase, redox state, and isotopic composition of these elements in materials from different geologic settings and from the atmosphere is significantly advancing this understanding.

## Assessment of Habitability

Mars provides a particularly compelling opportunity to study planetary evolution and the potential for early life because Mars has an ancient sedimentary rock record. Sedimentary rocks record past surface and subsurface environments, and similarly aged sedimentary rocks are the repository of evidence of early life on Earth. Additionally, the sedimentary record of Mars is largely older than the best-preserved ancient sediments on Earth, affording the potential to examine early climate and stages of microbial evolution, if life ever evolved on Mars (Grotzinger and Milliken 2012). Even if life never evolved on Mars, it is possible that prebiotic compounds are preserved in the sedimentary record of Mars, the study of which would provide constraints on the origin of life on Earth (Sasselov et al. 2020).

The MSL mission focuses in particular on the habitability of ancient Mars by investigating sites in the rover’s field area where geochemical and environmental indicators can be extracted from the geological record and compared with the requirements and limits of life (e.g., Cottin et al. 2017; Arvidson and Catalano 2018). By studying multiple sites, the range, diversity, and duration of habitable environments within Gale crater becomes apparent. Because these investigations also inform the future search for signs of ancient martian life, the mission seeks to identify those aspects of habitable environments, and the subsequent history of the rocks that record them, that are more conducive to preserving organic molecules or other potential biosignatures (Grotzinger 2014). For example, preservation of organic molecules might be favored in settings where they were initially concentrated and quickly protected (e.g., by early burial or mineralization) and subsequently received minimal exposure to oxidants or UV/high-energy radiation.

The selection of Gale crater as the mission’s landing site anticipated that the diversity of Mount Sharp’s strata, with multiple morphological and mineralogical indicators of aqueous environments inferred from orbiter data, would enable a comparative study of habitability. The team further hoped that the discovery of complex organic molecules, or other unusual chemical, mineralogical, or isotopic compositions that might be interrogated as potential biosignatures by future missions, would broaden the habitability assessment to also include taphonomy and preservation (Grotzinger et al. 2012). As described below, Gale crater indeed has revealed a variety of habitable environments, produced detections of diverse organic molecules, and yielded insights into preservation.

Some of Curiosity’s findings also are intriguing in the context of searching for potential biosignatures. Examples include atmospheric species in apparent disequilibrium such as oxygen and methane (Sect. 5.2), and isotopic compositions that differ significantly from expectations, such as highly depleted ^34^S (Franz et al. 2017b) and ^13^C (House et al. 2022). In all cases, non-biological explanations are available, and furthermore, the understanding of martian geochemical and isotopic processes is limited. More advanced instrumentation and/or returning samples to Earth by future missions would be necessary to take this assessment further (Farley et al. 2020).

### Evidence of Ancient Habitable Environments

#### Constraints on Temperature, pH, and Salinity

As described in Sect. 2, Curiosity’s investigations have led to the interpretation that the strata on the plains and foothills of Mount Sharp were deposited in fluvial, deltaic, and lacustrine settings over a period spanning at least millions to tens of millions of years. After burial and lithification, subsurface fluids pervaded the rocks in multiple episodes and with a variety of fluid compositions. With few exceptions, Curiosity’s observations of chemical and mineralogical composition are consistent with surface and subsurface fluids that remained within the ranges of temperature, pH, and salinity that could have supported life over much of the time that the fluids were present, indicating an extended period of habitable conditions within Gale crater.

Samples drilled from the Sheepbed mudstone at Yellowknife Bay on Aeolis Palus yielded evidence for early, in-place aqueous alteration of primary basaltic minerals in a lake and groundwater system characterized by cold temperatures (from the lack of chemical weathering), circum-neutral pH, and low salinity (Grotzinger et al. 2014; McLennan et al. 2014; Ming et al. 2014; Vaniman et al. 2014). The kinetics of alteration require that such conditions persisted for thousands to hundreds of thousands of years (Bristow et al. 2015). At both Yellowknife Bay and the Kimberley, analyses of drilled samples indicate that minimal weathering of the parent rocks occurred, perhaps because a relatively cold climate was prevalent when the sediment was eroded and transported.

Compared with the Bradbury group, the rocks of the Mount Sharp group have experienced greater chemical weathering and have interacted with fluids with a greater range of salinity and pH (Sect. 2). However, there is little evidence for environmental conditions that would have posed a significant challenge to microbial metabolism. Curiosity has found no evidence for frozen conditions when the lakes were present, nor evidence of authigenic minerals formed by alteration at high temperature (e.g., chlorite). Hydrated calcium sulfate observed in fracture fills throughout the rover’s traverse is consistent with nonacidic fluids and moderate temperatures (< 60°C) (Nachon et al. 2014; Rapin et al. 2016). Mineral phases that may have formed in a low-pH environment, such as iron sulfates, occur in minor abundances and might reflect late-stage, localized conditions.

#### Geochemistry Relevant to Habitability

Rocks within Gale crater bear H, C, N, O, P, S, and other elements of importance to biological processes (e.g., Grotzinger et al. 2014). In addition, they show chemical and/or mineralogic evidence for redox disequilibrium, such as the presence of mixed valence iron- (e.g., magnetite, akaganeite) and sulfur-bearing (sulfide, sulfate) minerals, indicating environments that could have been exploited by chemoautotrophic microorganisms (Grotzinger et al. 2014; Wong et al. 2020). Iron oxidation associated with phyllosilicate formation (Bristow et al. 2015) and hypothesized redox stratification within lakes (Hurowitz et al. 2017) also may have provided chemical energy for life (Arvidson and Catalano 2018).

Trace amounts of oxidized nitrogen-bearing compounds were found in the inactive Rocknest sand deposit and in lacustrine mudstones at abundances that are comparable with the Dry Valleys of Antarctica (Stern et al. 2015). The presence of fixed nitrogen (the form available to biology) in the form of nitrate may be evidence for part of an Earth-like nitrogen cycle. The oxidized nitrogen could have been produced by volcanic, impact, or photochemical processes (Stern et al. 2015; Navarro-González et al. 2019). Nitrogen may have been a limiting nutrient in regions such as Glen Torridon, however, where drilled samples lack nitrate (Sutter et al. 2020).

Minor amounts of boron, considered important for prebiotic chemical synthesis, were detected in calcium sulfate-bearing veins within the Yellowknife Bay, Murray, and Stimson formations, likely as borate (Gasda et al. 2017; Das et al. 2020). Its mineral association is unknown. Boron that initially weathered from the crust may have been concentrated in lacustrine sediments or (unidentified) evaporites before being remobilized in fluids that moved through fractures after lithification. Das et al. (2020) suggest that a weak inverse correlation between boron and lithium with stratigraphic height may record the precipitation sequence of the diagenetic fluids.

### Organic Molecules, Taphonomic Windows, and Preservation

Curiosity’s inventory of carbon and organic molecules has shown that this “raw material” was available for potential life. Organic matter also can be a food source, implying that energy sources were present for both chemoautotrophy and heterotrophy (Sutter et al. 2017; Eigenbrode et al. 2018). Organic molecules, which may be produced abiotically or delivered by impactors, are subject to transformation and degradation by oxidation as well as UV and high-energy radiation. Curiosity’s discoveries, however, demonstrate that organic matter has been preserved over geologic time.

#### Detections of Carbon and Organic Molecules

Organic molecules are detected by analyzing gases evolved from heated samples in a mass spectrometer either in bulk or after separation using gas chromatography. The team identified dichloroalkanes (C_2_ to C_4_) and chlorobenzene (C_6_) in the Cumberland lacustrine mudstone sample from Yellowknife Bay (Freissinet et al. 2015), in addition to sulfur-bearing organics released at high temperature (Eigenbrode et al. 2018). Although the chlorine and carbon are martian in origin, the chlorinated hydrocarbons are thought to be the result of pyrolysis degradation and chlorination of more complex precursor molecules—perhaps benzoic acid (Millan et al. 2020; Freissinet et al. 2020; Miller et al. 2016)—by oxychlorine compounds such as perchlorate (Freissinet et al. 2015; Glavin et al. 2013). Laboratory work and a targeted search led to the additional detection of trichloromethylpropane and two to three isomers of dichlorobenzene (Szopa et al. 2020).

Samples from the Pahrump Hills area of lower Mount Sharp show a diversity of organic molecules including aromatic and aliphatic (C_1_ to C_5_), as well as thiophenic (organic sulfur) structures (Eigenbrode et al. 2018). They were detected in gases that evolved above the temperatures that most instrument-derived organics are observed, and also above the temperature of perchlorate decomposition. These results are consistent with fragments released by pyrolysis from geologically refractory organic matter in the form of large macromolecules (e.g., a kerogen-like material), or organic matter released at high temperature from sulfates (François et al. 2016).

The Sample Analysis at Mars (SAM) instrument suite has a derivatization capability using liquid MTBSTFA:DMF sealed in seven sample cups (Mahaffy et al. 2012). It targets more complex and refractory organics such as amino acids, carboxylic acids, alcohols, and nucleobases in solid samples, and thus may help to identify additional organic molecules as well as precursors of the chlorinated hydrocarbons detected previously. Early in the mission it was found that vapor from the cups had leaked into the instrument (Glavin et al. 2013). The leak created an opportunity to search for products of the reaction between the vapor and organic molecules in samples cached within the instrument. Analysis of the Cumberland sample after exposure to the vapor showed the presence of decane (C_10_), dodecane (C_12_), and tentatively undecane (C_11_), likely tied to sulfate decomposition (Freissinet et al. 2019).

During the time that the rover’s drill was unavailable for use (Sect. 8.1), the first nominal derivatization experiment was performed using sand scooped from the Bagnold dune field at Ogunquit Beach (Millan et al. 2021b). Analyses confirmed the presence of derivatized benzoic acid and ammonia, mass spectra matching derivatized phosphoric acid and phenol, nitrogen-bearing molecules, and a number of unidentified high-molecular-weight compounds, although work remains to rule out sources from within the instrument. The Ogunquit Beach experiment demonstrated the capability of the derivatization technique to extract molecules not detectable in standard SAM runs and helped optimize experimental protocols for future derivatization analyses. Within the Glen Torridon region, multiple SAM runs, including a derivatization experiment on a sample drilled from the smectite-bearing Knockfarril Hill member at Glen Etive, revealed a diversity of organic molecules, including a wider suite of sulfur-bearing organics and high molecular weight organics (Millan et al. 2021a). Work continues to identify molecular precursors and extract additional results from the experiments mentioned above.

Evolved gas analyses indicate CO_2_ and CO in quantities well above background. Their abundance cannot be explained simply by the carbon in the instrument reacting with martian oxidants or by inorganic minerals in samples. The most plausible explanations involve the release of CO_2_ and CO from carboxylated phases, including salts of organic acids produced by the degradation of organic matter, and oxidation of carbon molecules derived from the martian sediments (Franz et al. 2020; Sutter et al. 2017; Lewis et al. 2021). These indirect measurements of organic molecules and organic salts, along with the direct detections and inferences from laboratory work, suggest that hundreds or even thousands of parts per million (by weight) of organic molecules may have been present in the sediments within Gale crater (Sutter et al. 2017; Freissinet et al. 2020). Importantly, Sutter et al. (2017) note that even the amount of organic carbon *directly* detected is comparable with that found in oligotrophic (nutrient-limited) environments that sustain microbial populations on Earth.

#### Taphonomic Windows and Preservation

In the context of the MSL mission, taphonomic windows refer to particular sets of sedimentologic or diagenetic processes that, integrated over geologic time, favor preservation of organic molecules or other potential biosignatures (McMahon et al. 2018; Tan and Sephton 2020; Grotzinger et al. 2012; Summons et al. 2011). Following the recommendations of its Biosignature Working Group (Summons et al. 2011), the mission sought a field area with sediments deposited in deltaic and lacustrine settings, which are known to concentrate organics on Earth, as well as concentrations of crystalline minerals, including phyllosilicates and sulfates, that are capable of absorbing and/or encapsulating organics. Curiosity also has encountered finely laminated, fine-grained mudstones enriched in amorphous silica (Hurowitz et al. 2017), a combination that could be favorable for preservation (McMahon et al. 2018). Rocks with significant abundances of halite, carbonates, or phosphates have not been encountered. In terms of threats to preservation, no volcanic or hydrothermal sources have been observed in the field area, nor is there evidence for thermal metamorphism or major structural alteration.

The distribution and inventory of organic molecules detected by Curiosity point to potential pathways to preservation (Eigenbrode et al. 2018). One is the association of organic material with smectite-bearing, fine-grained, lacustrine mudstones. Second, both the sulfidic and potentially macromolecular nature of the organic matter suggest that these characteristics were important to its preservation, as is commonly observed in coal and oil shale on Earth, as well as in carbonaceous chondrites. Sulfate minerals also may contribute to preservation, as they are known to preserve amino acids from decarboxylation in terrestrial settings (Aubrey et al. 2006).

Ionizing radiation is uniquely challenging to preservation, since Mars lacks the thick atmosphere and magnetic field which greatly reduce radiation at Earth’s surface. Levels of present-day ionizing radiation measured by Curiosity place severe constraints on preservation at the surface (Hassler et al. 2014), but recently exhumed rocks would be less impacted. The young cosmogenic exposure age of the Sheepbed mudstone at Yellowknife Bay, combined with its location near an outcrop of an overlying sandstone, suggests that the organic molecules detected there had been protected from degradation by high-energy radiation until the sandstone was removed relatively recently (∼80 Ma) by erosion (Farley et al. 2014). Geologic mapping can identify places where rocks that record ancient environments have been protected and only recently exposed by ongoing geologic processes.

Some aspects of the mission’s detections of organic molecules are somewhat surprising. For example, organics were detected in the Mojave sample from Pahrump Hills, in spite of evidence for multiple episodes of fluid-driven diagenesis, including acidic and oxidizing fluids (Rampe et al. 2017; Eigenbrode et al. 2018; Kah et al. 2018; Martin et al. 2017). Organic molecules were detected (although not yet confirmed as indigenous) in the Glen Etive drill sample within the Knockfarril Hill member, interpreted to have been deposited in a higher-energy, lake margin environment (Millan et al. 2021a; Fedo et al. 2020). Perhaps most surprising, if confirmed, is the variety of organics found in sand from an active sand sheet at Ogunquit Beach (Millan et al. 2021b), given the exposure to the surface environment. These findings may indicate that preservation is possible over a wide range of depositional environments, diagenetic histories, and surface exposure.

## Modern Aeolian Activity

Signs of wind-driven erosion and sediment transport are evident all along Curiosity’s traverse. Examples include atmospheric dust and dust lifting (Sect. 5), surfaces cleaned of air-fall dust by wind or aeolian abrasion, ventifacts, sandy deposits worked into ripples, and fresh dune slipfaces. Bedforms range in size from cm-scale ripples, to isolated meter-scale ridges and megaripples, to dunes. Curiosity has observed active saltation in association with deposits of well-sorted, fine-grained sand. Deposits that also have a significant component of coarser grains are interpreted to be modern but presently inactive, perhaps last mobilized during a different climate regime. Given the dominant role of aeolian processes in modifying the modern landscape (Day and Kocurek 2016), as well as their application to extracting paleoenvironmental indicators from the sedimentary rock record, the mission has dedicated significant time to their evaluation. Curiosity has completed the most comprehensive in situ study of martian aeolian processes to date.

### Inactive Aeolian Deposits

The mission’s first sample was scooped from Rocknest, a small aeolian deposit in the lee of nearby cobbles. Trenches created by the rover’s wheel and scoop revealed a surface layer dominated by ∼1 mm grains overlying an interior of finer sand. This bimodal size distribution is consistent with aeolian transport by combined saltation and saltation-induced creep, similar to coarse-grained ripples observed on Earth and observed on Mars at Gusev crater and Meridiani Planum (Minitti et al. 2013; Weitz et al. 2018). Induration (e.g., standing trench walls) and a thin mantle of dust on surface grains indicate that the Rocknest deposit is not currently active. Additional examples of indurated bedforms with coarse-grained and dusty surfaces were occasionally observed along Curiosity’s traverse, including the small transverse aeolian ridge at Dingo Gap (Zimbelman and Foroutan 2020), and near active deposits of finer sand in Glen Torridon.

Material scooped from Rocknest has a basaltic composition typical of martian soils (i.e., mixed unconsolidated material) measured by other landed spacecraft. X-ray diffraction did not reveal any crystalline hydrated phases, but found that a few 10s of % of the scooped material is X-ray amorphous (Blake et al. 2013; Bish et al. 2013). Water and other volatiles detected through evolved gas analysis likely came from the amorphous component (Leshin et al. 2013). Abundances of S and Cl are consistent with a component of typical atmospheric dust (Berger et al. 2016).

### The Bagnold Dune Field and Other Active Aeolian Deposits

The extensive Bagnold dune field was investigated in a two-phase campaign between 2015 and 2017, comprising the first in situ study of an active dune field on another planet. Phase one focused on barchan and barchanoid dunes along the northern margin of the dune field in southern fall/winter, while phase two examined a related linear dune and extensive ripple field during the windier southern summer (Bridges and Ehlmann 2018; Lapotre and Rampe 2018). Saltation in summer was vigorous enough for sand to accumulate on the rover’s deck and to enter the Mast Cameras’ (Mastcam) baffles ∼2 m above the surface. The campaign sought to characterize the physical properties of the sand and their variation along bedform; to classify and interpret dune and ripple morphologies; to monitor activity and correlate it with measured and modeled winds; to determine chemical and mineralogical composition and how they vary along bedform and across the dune field; and to compare with results derived from orbiter observations.

**Fig. 7:**
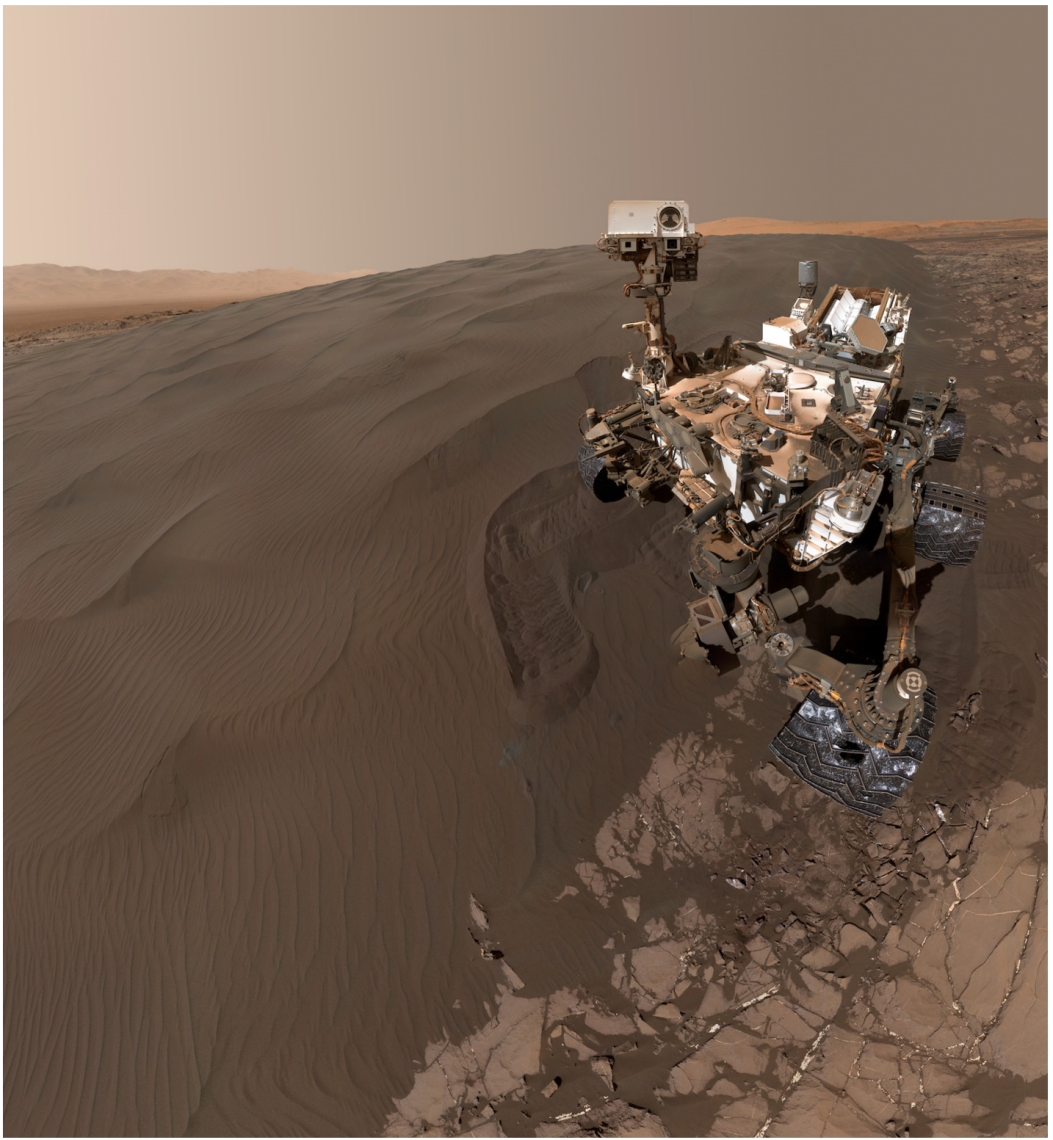
Curiosity at the Gobabeb site on Namib dune on Sol 1228. The MAHLI mosaic shows the primary (meter-scale) and secondary (cm-scale) ripples on the dune as well as an arcuate trench intentionally created using the rover’s wheel (image credit: NASA/JPL-Caltech/MSSS)

The Bagnold dune field consists of fine to very fine sand, with minor but variable fractions of coarser particles near larger ripple crests. Many aspects of dune and superimposed ripple morphologies are recognizable from terrestrial settings. One exception is the prevalence of large ripples of meter-scale wavelengths without coarse, creep-limited grains covering their crests (Fig. 7), distinct from the smaller-scale impact ripples that are common to both planets. Lapotre et al. (2016, 2018) argue that wind drag in the higher kinematic viscosity of the martian atmosphere limits the size of these ripples. Sullivan and Kok (2017) and Sullivan et al. (2020) suggest that impact ripples may be able to grow to larger scales on Mars given the much lower wind dynamic pressures affecting ripple crests.

Composition is dominated by basaltic minerals with a significant X-ray amorphous component (Achilles et al. 2017). Although broadly similar to soils from Meridiani Planum, Gusev, and other sites in Gale, the Bagnold sands are chemically distinct. This is attributed to effects of activity, such as compositional sorting, lack of chemical alteration, and lack of dust (Cousin et al. 2017b; O’Connell-Cooper et al. 2017). Some coarser particles appear to be fragments derived from the erosion of local bedrock and mineral veins (Weitz et al. 2018).

Relative to most bedrock samples, aeolian materials are enriched in O_2_ and NO, which may derive from oxychlorine and nitrate, respectively (Stern et al. 2018). A possible explanation is that sand has accumulated oxychlorine and nitrate from the atmosphere with limited subsequent exposure to water. In evolved gas experiments, carbon was evolved as CO_2_ in greater concentrations and at higher temperatures compared to rock samples (Stern et al. 2018). Possible sources are carbonates and organic molecules that were combusted during the experiment. Both the higher temperature and enrichment in ^13^C are consistent with the presence of siderite (Franz et al. 2020). If present at similar concentration in globally distributed sands, the carbonate would hold the equivalent of about half a bar of atmospheric CO_2_.

A key objective of the team was to better understand the processes and conditions required to initiate and sustain saltation, particularly when it was possible to coordinate imaging of grain motions with Rover Environmental Monitoring Station (REMS) wind measurements (Bridges et al. 2017; Baker et al. 2018b). Abundant grain and ripple motions were observed despite wind speeds that only rarely (if ever) exceed the fluid threshold friction speed required to initiate saltation. This led to the development of a new saltation model that accounts for the initiation and sustainability of low-flux sand transport with wind speeds below the fluid threshold but above the impact threshold (Sullivan and Kok 2017).

Using observations away from large aeolian deposits, Baker et al. (2018a) found that the wind-induced movement of isolated particles on bedrock and the migration of small ripples both have a strong seasonal dependence. Mobilization occurs only during southern spring and summer, consistent with expectations of stronger winds around perihelion. Similar to what was observed for smaller particles in the dune field, particles 0.5 to 3 mm in diameter also were observed to move in spite of modeled winds falling below theoretical thresholds. Baker et al. (2018a) argue that such observations highlight the need to broaden interpretive tools beyond classical transport models in order to account for gusts and alternate modes, such as drag-induced rolling and impact-driven creep.

## Atmospheric Evolution and the Modern Environment

Curiosity’s payload and measurement strategies have contributed significantly to the understanding of the long-term evolution of the martian atmosphere, as well as its present state and behavior. The mission has collected an unprecedented, nearly continuous hourly record of key meteorological variables spanning more than four Mars years, along with compositional measurements, imaging, and spectroscopy. It is the most comprehensive meteorological record ever gathered from the martian surface (Martínez et al. 2017).

SAM analyzes the chemical and isotopic composition of the atmosphere with its Quadrupole Mass Spectrometer (QMS) (CO_2_, N_2_, Ar, O_2_, CO, Kr, Xe,) and Tunable Laser Spectrometer (TLS) (CH_4_, H_2_O, and CO_2_). REMS sensors on two short booms located ∼1.6 m above the surface measure wind speed and direction (through Sol ∼1500), air temperature, relative humidity, and ground brightness temperature. REMS monitors pressure from inside the rover through an inlet port and measures six spectral bands of UV irradiance using sensors on the deck. The Mastcam and Navigation Camera (Navcam) imagers view the sun, sky, and horizon in order to measure atmospheric optical depth in the vertical column and horizontal line-of-sight, water-ice cloud morphology and motion, and aerosol optical properties. They also view the surface to capture dust lifting, dust devils, and wind-induced movement of fines (Sect. 4). The Chemistry and Camera (ChemCam) spectrometer in passive mode measures aerosol optical properties and column water vapor and oxygen abundance.

REMS measurements are collected at 1 Hz for at least five minutes each hour, twenty-four hours per sol. In addition, several full hours are distributed within each sol such that coverage of the entire diurnal cycle at 1 Hz is achieved every few sols. Imaging of atmospheric phenomena repeats on scales of sols to weeks, while SAM experiments are carried out seasonally. The science operations team maintains a comprehensive planning schedule such that Curiosity’s observations are optimized to capture phenomena with characteristic timescales of seconds to years.

Curiosity’s field site imparts both opportunities and challenges. The rover’s equatorial site complements the meteorological records of past missions. Gale crater and Aeolis Mons are major topographic features that alter regional winds and atmospheric tides, constrain the mixing of gases and aerosols with the outside atmosphere, and generate local circulations and pressure gradients (Tyler and Barnes 2013; Rafkin et al. 2016; Richardson and Newman 2018). This complexity is fascinating to explore, but also results in measurements with convolved local, regional, and planetary-scale signals. Measurements made along the traverse help correlate phenomena with topographic settings and elevation.

### Atmospheric Reconstruction from Entry and Descent

The mission’s first atmospheric science results came from data collected during the spacecraft’s descent and were analyzed along with measurements from MRO and REMS (post-landing). The Mars Entry, Descent, and Landing Instrument suite (MEDLI) included a set of sensors built into the heat shield that measured dynamic pressure (Karlgaard et al. 2014). The rover also recorded 3-axis accelerations and rotations from which atmospheric properties were derived using the spacecraft’s aerodynamic characteristics. The result is a reconstruction of density, temperature, and pressure along the spacecraft’s trajectory (Chen et al. 2014; Holstein-Rathlou et al. 2016) with a spatial resolution and vertical extent that exceed what is possible from orbit or the surface. The reconstructed atmosphere follows the MRO-MCS temperature profile below ∼70 km but resolves additional vertical structure. Large thermal oscillations at higher altitudes, including a deep minimum near 81 km, are suggestive of thermal tides. The reconstruction also allows assessments of the atmospheric characterization process (Vasavada et al. 2012) that informed the design and execution of the EDL system, and the numerical models and orbiter observations that fed it. Chen et al. (2014) note the overall soundness of the approach along with some discrepancies. The reconstructed atmosphere deviates from numerical model results by up to 10% in density and pressure in the middle atmosphere, but remains within assumed uncertainty estimates. There also is evidence of an unexpectedly strong north-to-south crosswind during the spacecraft’s multiple bank reversals at ∼ 11 km altitude.

### Chemical and Isotopic Composition

The SAM QMS and TLS have determined volume mixing and isotope ratios in repeated measurements with unprecedented precision. These measurements have refined our knowledge of the atmosphere’s chemical and isotopic composition, provided strong support and new detail to the hypothesis of substantial atmospheric loss over Mars’ history, and revealed diurnal and seasonal methane activity.

#### Atmospheric Composition

Curiosity’s precise and regular measurements of all major atmospheric species provide unique insights into their behaviors. SAM confirmed that CO_2_ (annual mean mixing ratio of 0.951), N_2_ (0.0259), ^40^Ar (0.0194) and O_2_ (0.00161) are the most abundant by volume (Trainer et al. 2019; Franz et al. 2017a; Mahaffy et al. 2013) and found ^40^Ar/^14^N to be 0.376±0.0008, refining the values of 0.3 and 0.35 measured by Viking Landers (Trainer et al. 2019). Abundances published early in the mission (Mahaffy et al. 2013) required updating after the SAM instrument calibration was refined using an onboard calibration cell (Franz et al. 2017a). Adjustment of surface pressure by the polar condensation and sublimation of CO_2_ occurs 20-40° of $L_{\mathrm{s}}$ ahead of adjustment of composition in northern summer, revealing that transport outpaces mixing (Trainer et al. 2019). The abundance of O_2_ does not vary in parallel with the other non-condensable gases. Its observed large range (1300 to 2200 ppmv), gradual increase in northern spring/summer, and interannual variability are not currently explained by known sources and sinks (Trainer et al. 2019).

#### Atmospheric Evolution from Isotopic Composition

Isotope ratios in the atmosphere represent the cumulative effects of mass-dependent fractionation occurring as gases are supplied to the atmosphere, lost to space, and exchanged with surface/subsurface reservoirs. The ^40^Ar/^36^Ar ratio measured by SAM QMS confirms earlier results from Mars meteorites and indicates that the nearly complete loss of Mars’ primordial atmosphere through hydrodynamic escape has allowed the radiogenic isotope to become dominant (Mahaffy et al. 2013). Xe isotopes provide additional evidence of this early loss (Conrad et al. 2016).

The low ^36^Ar/^38^Ar ratio compared with other solar system objects implies that the secondary atmosphere also has experienced significant loss (50% and perhaps as high as 85-95%) (Atreya et al. 2013). Modeling of ion sputtering constrained by Curiosity and MAVEN observations suggests that two thirds of atmospheric argon has been lost to space (Jakosky et al. 2017). Multiple Kr isotopes also preserve evidence of loss processes in the secondary atmosphere (Conrad et al. 2016).

Mahaffy et al. (2013) note that the SAM-QMS measured $\delta ^{13}$C to be ∼45 per mil, a value that was independently confirmed in CO_2_ by the TLS (Webster et al. 2013). They interpret this enrichment in the heavier carbon isotope to be a signature of significant loss of carbon from Mars’ secondary atmosphere. Both the carbon and argon results are more precise and closer to those measured in meteorites of likely martian origin than are earlier Viking values, providing additional strong support for the meteorites’ martian origin.

SAM-TLS completed the first in situ measurement of $\delta $D in water vapor, finding it to be ∼5000 per mil (D/H ∼6), indicating significant enrichment of HDO via photolysis and loss of the lighter isotope to space, with an unknown contribution from seasonal cycling with the surface (Webster et al. 2013). The $\delta ^{15}$N of from SAM-QMS also reflects escape of the secondary atmosphere, although exchange with the surface is poorly understood (Wong et al. 2013).

D/H in water evolved from Hesperian smectite clay minerals is intermediate between the present atmospheric value and the primordial value, suggesting that a larger exchangeable water inventory existed at ∼3.6 Ga when the clay minerals formed in an ancient lake and groundwater system within Gale crater (Mahaffy et al. 2015).

#### Transient and Seasonal Methane

Curiosity’s precise, in situ measurements of atmospheric methane have revealed a persistent but seasonally variable background abundance (0.25 to 0.65 ppbv) as well as transient spikes (up to ∼20 ppbv) (Webster et al. 2018, 2021). The background level is about six times lower than model predictions of methane produced by UV-irradiation of carbonaceous planetary infall (i.e., dust, meteoritic, cometary), but the conversion rate of infall material is poorly constrained (Schuerger et al. 2012). The transient spikes currently defy explanation but are consistent with small, localized sources of methane released from surface or subsurface reservoirs.

The seasonal variation is much greater than that predicted from either UV degradation of impact-delivered organics or from passive enrichment as a non-condensable gas. It can be explained if release of methane from the subsurface (e.g., produced by serpentinization) is moderated by near-surface temperatures and/or winds (Moores et al. 2019a; Viúdez-Moreiras et al. 2020). Such methane releases would be expected to accumulate in the near-surface atmosphere at night, due to a combination of nocturnal inversion and convergent downslope winds, and to be rapidly diluted by convection and circulation during the day (Pla-Garcia et al. 2019; Moores et al. 2019b). This explanation is consistent with day-night differences measured by Curiosity (Webster et al. 2021) and with the daytime non-detections both from Curiosity and from ExoMars Trace Gas Orbiter measurements at higher altitudes (Montmessin et al. 2021; Korablev et al. 2019; Knutsen et al. 2021). Assuming that methane is produced over an area wider than Gale crater, yet-unidentified rapid sequestration and/or destruction processes are required to keep levels below the upper limits measured from orbit (Moores et al. 2019b; Montmessin et al. 2021). Processes that would more definitively reconcile the surface and orbiter measurements continue to be pursued, as well as sources of error (Webster et al. 2018).

### Atmospheric Pressure

Curiosity’s record of atmospheric surface pressure captures a rich variety of phenomena over a range of temporal and spatial scales, including seasonal changes in atmospheric mass, planetary-scale and synoptic circulations, atmospheric tides, convective vortices, and wind-induced fluctuations (Gómez-Elvira et al. 2014; Haberle et al. 2014; Harri et al. 2014a; Guzewich et al. 2016).

#### Atmospheric Tides and Hydrostatic Readjustment

The amplitude of the diurnal pressure variation can exceed 10% of the mean, several times larger than at the Viking Lander sites (Harri et al. 2014a; Haberle et al. 2014; Guzewich et al. 2016). About half of the variation is due to the synoptic-scale distribution of topography and surface properties that cause constructive interference between the westward-propagating Sun-synchronous tide and the eastward-propagating Kelvin wave. The other half arises as the atmosphere hydrostatically readjusts to Gale crater’s topography in response to the large diurnal cycle in near-surface air temperature (Richardson and Newman 2018).

Tidal theory indicates that the diurnal tide is a sensitive indicator of tropical and local atmospheric heating by dust, while the semidiurnal tide is forced over planetary scales. Guzewich et al. (2016) find that the amplitudes of the diurnal and semidiurnal modes are strongly correlated (∼0.9) with the local dust optical depth, indicating that atmospheric dust over Gale crater varies in sync with global dust loading. There are exceptions when discrete storms cause local and distant dust conditions to diverge.

#### Equator-Crossing Transient Eddies

Orbiter imaging and numerical models show that dust-lifting fronts in the northern hemisphere during fall through spring are associated with traveling baroclinic systems, and that they can cross into the southern hemisphere and trigger additional dust lifting (Wang et al. 2003; Hinson and Wang 2010). Haberle et al. (2018) found tiny (∼3 Pa) pressure signatures of associated transient eddies using REMS, confirming that the systems are equator-crossing.

#### Fluctuations from Convection and Gravity Waves

Ullán et al. (2017) examine small pressure fluctuations that recur on different sols and evolve with season. They interpret these fluctuations as dynamic pressure events (i.e., induced by wind) and find correlated fluctuations in air and ground temperature. One class of fluctuations that occurs in the daytime with a maximum amplitude of 0.2-0.3 Pa and an annual mean duration of 40 s is attributed to insolation-driven boundary layer convection. Air temperatures increase after sunrise, peak in the late afternoon well after the insolation maximum, then fall rapidly in the early evening before decreasing slowly throughout the night. This behavior and the greater variability in the afternoon are signatures of convection (Pla-Garcia et al. 2016).

Overnight pressure fluctuations of ∼1 Pa with durations of 1-10 min, as well as correlated fluctuations in air and ground temperature, are attributed to gravity waves excited by downslope flows, also known as “mountain waves” (Harri et al. 2014a; Haberle et al. 2014; Pla-Garcia et al. 2016; Rafkin et al. 2016; Ullán et al. 2017).

#### Convective Vortices and Dust Devils

Transient pressure drops (< 5 Pa and < 10 s) are interpreted to be caused by passing convective vortices in the daytime and by mechanically forced turbulence at night (Haberle et al. 2014; Moores et al. 2015a; Kahanpää et al. 2016; Ordóñez-Etxeberria et al. 2018). They occur at all seasons, with the largest amplitude and frequency in southern spring and summer. The daytime population peaks around noon and appears to be dominated by those insufficiently strong to lift dust, likely a result of intercrater overturning circulations that suppress the convective boundary layer (Tyler and Barnes 2015). The frequency of daytime pressure drops more than doubled as Curiosity reached and ascended Mount Sharp. Image detections of dust devils and lifting events also increased. These increases are attributed to lower surface thermal inertia, higher elevation, and stronger winds (Newman et al. 2019; Ordóñez-Etxeberria et al. 2020).

### Atmospheric Dust

#### Dust Optical Depth

Aerosol optical depth above Gale crater has been measured every few sols by observing the sun with Mastcam using techniques described in Lemmon et al. (2015). It is dominated by dust but a small fraction comes from water ice in the colder seasons (Kloos et al. 2018). The seasonal variation over Gale crater generally follows the global dust climatology seen from orbit and other landed missions. The optical depth at 880 nm decreases throughout southern fall and early winter to ∼ 0.3 until abruptly rising to ∼ 0.7 at $L_{\mathrm{s}}$=135-140° as dust activity occurs in the northern hemisphere. It rises again after $L_{\mathrm{s}}$=210° in southern spring when Mars’ primary dust-lifting season gets underway. It stays elevated over the remainder of southern spring and summer but is variable both within a given year and between years. Each year there are local maxima near $L_{\mathrm{s}}$=220-250° and $L_{\mathrm{s}}$=310-340°, separated by a local minimum near $L_{\mathrm{s}}$=270-300°.

Smith et al. (2016) characterized numerous transient but significant changes in optical depth by controlling the hourly REMS UVS data to the smoothed Mastcam trend in order to account for the gradual accumulation and removal of dust on the UVS sensor windows. These short-lived variations likely represent dust lifting closer to Gale crater.

A coarse vertical profile of the dust mixing ratio above Gale crater and its seasonal evolution can be assembled from horizontal extinction derived from the Navcam (Moores et al. 2015a; Moore et al. 2016), column optical depth from REMS UVS and Mastcam, infrared sounding of the planetary limb from the MRO Mars Climate Sounder, and a general circulation model. Guzewich et al. (2017) find that above the planetary boundary layer (PBL), dust evolution largely follows that of the surrounding atmosphere, reaching a maximum abundance and altitude in the perihelion season near southern spring and summer. Dust within the crater varies similarly but more smoothly, moderated by the rate of mixing both vertically and horizontally. Air inside the crater becomes starved of dust during the aphelion season when a thinner PBL restricts the exchange of air over the crater rim (Rafkin et al. 2016; Fonseca et al. 2018).

Dust was found to be removed from rover surfaces during the more dusty and dynamic perihelion season and deposited in southern fall and winter (Vicente-Retortillo et al. 2018). While dust accumulates on both horizontal and vertical rover surfaces, vertical surfaces experience more efficient cleaning and less net accumulation (Yingst et al. 2020). Gale crater may be a net source of dust in southern spring and summer (Moore et al. 2019).

#### Dust Aerosol Properties

Vicente-Retortillo et al. (2017) and Smith et al. (2016) used REMS UVS and Mastcam optical depth measurements to determine that the effective (area-weighted) mean radius of atmospheric dust varies from about 0.6 μm during the low-opacity season to 2 μm during the high-opacity season, affecting the rates of atmospheric heating, transport, and settling. The shift toward larger particles in Mars’ dusty season is a planetwide phenomenon that also has been described in MER and orbiter data (Lemmon et al. 2015).

### Winds and Circulation

Wind patterns within Gale crater are the result of complex interactions between planetary scale (e.g., the mean meridional circulation, thermal tides), regional (e.g., slope winds on the martian topographic dichotomy), and local (e.g., slope winds on the crater rim and Aeolis Mons) circulations that are driven by diurnal and seasonal forcing (Tyler and Barnes 2013; Rafkin et al. 2016; Baker et al. 2018a). Viúdez-Moreiras et al. (2019a) derived wind speed and direction from REMS through sol ∼1500, taking care to account for the effects of rover structure and degradation of the wind sensors. The observed winds can be tied to the individual circulation components listed above as they vary in dominance over diurnal and seasonal cycles. Slope winds on Aeolis Mons are the overall dominant component of the circulation near Curiosity, with the largest speed midday and near the equinoxes (Rafkin et al. 2016; Viúdez-Moreiras et al. 2019b). A dedicated experiment characterized how a large dune influenced local wind patterns (Newman et al. 2017).

### Water Vapor and Water Ice Clouds

Relative humidity at 1.6 m above ground is very low during the day and higher during the colder night, but never saturates (Gómez-Elvira et al. 2014; Harri et al. 2014b). It peaks in the southern winter. If water vapor pressure at the surface is assumed to be the same as at 1.6 m, measured surface temperatures indicate that the frost point is reached for several nights each fall and winter and a few nights in spring and summer (Martínez et al. 2016). However, several attempts at detecting frost have resulted in only a tenuous result (Gough et al. 2020).

Column water vapor measured by ChemCam exhibits a seasonal behavior similar to that of orbiter measurements, but near-surface mixing ratios derived from REMS are depressed at night and rapidly increase at sunrise, consistent with temperature-dependent adsorption by surface materials (Savijärvi et al. 2016, 2019a; McConnochie et al. 2018; Steele et al. 2017). Curiosity’s instruments do not have the sensitivity to directly measure this adsorption. The inferred adsorption increases over sand and decreases over bedrock (Martín-Torres et al. 2015; Savijarvi et al. 2019b).

The presence, diurnal and seasonal variability, morphology, altitude, vertical structure, dynamics, and scattering properties of thin (optical depth < 0.05) water ice clouds over Gale crater have been determined from regularly acquired Navcam observations (Moores et al. 2015b; Campbell et al. 2020; Cooper et al. 2019). The Aphelion Cloud Belt brings elevated water ice cloud opacity and cloud activity over Gale crater in southern autumn and winter. Cloud opacity is greatest in the morning with a secondary peak in mid-afternoon (Kloos et al. 2016, 2018).

### 2018 Planet-Encircling Dust Event

Curiosity provided in situ meteorological observations throughout the onset and decay phases of the 2018 planet-encircling dust event, executing a pre-conceived campaign (Guzewich et al. 2019; Viúdez-Moreiras et al. 2019c; Savijärvi et al. 2020). Dust optical depth reached 8.5, resulting in a 95% decrease in incident UV insolation, in spite of no dust lifting observed locally within Gale. Dust effective radius increased by a factor of 3 (Lemmon et al. 2019). The team also noted a cessation of vortex activity, higher water vapor mixing ratios, and both local and planetary-scale signatures of dust loading in the diurnal pressure cycle. Little change to wind-driven sediment transport was observed during the event.

## High-Energy Radiation Environment

Curiosity’s Radiation Assessment Detector (RAD) has characterized energetic particle radiation continuously since Curiosity launched. Understanding how the broad spectrum of radiation from the sun and space is modulated by solar activity, the martian atmosphere, and the surface is of interest because radiation that reaches the martian surface poses a hazard to potential martian life and inhibits the preservation of biosignatures (Sect. 3). Furthermore, knowledge of the radiation environment is necessary to quantify the health risk to crewed missions and to inform mitigation strategies (Guo et al. 2021). Through Sol 2844 the RAD investigation was conducted jointly by NASA’s Science Mission Directorate and Human Exploration and Operations Mission Directorate.

RAD observations span both cruise, during which five solar particle events were detected (Zeitlin et al. 2013; Ehresmann et al. 2016), and for the first time, the surface (Hassler et al. 2014; Ehresmann et al. 2014; Köhler et al. 2014). The flux at the surface is modified by shielding from the planet as well as from the atmosphere as the atmospheric mass above Gale varies over diurnal and seasonal time scales (Rafkin et al. 2014; Guo et al. 2015). Hassler et al. (2014) used RAD results to estimate the radiation dose for a notional round-trip crewed mission. The RAD team also facilitated a first-of-its-kind workshop in which participants used RAD data to evaluate widely used radiation transport models (Matthiä et al. 2017). Such models are critical for assessing the radiation hazard to crewed missions under solar conditions different than present during the MSL mission.

Radiation at Mars comes from galactic cosmic rays and solar energetic particles. Between 2015 and 2018, the galactic cosmic ray induced dose rate increased by 50% as a consequence of the declining solar cycle (Zeitlin et al. 2019), and continues to increase. The flux of solar energetic particles can vary by a few orders of magnitude in response to coronal activity, and major solar storms can accelerate particles to energies high enough to propagate through the martian atmosphere to the surface. RAD has become an important outpost for monitoring space weather in the inner solar system. When combined with other solar observatories, RAD data permit tracking of the interplanetary propagation of solar magnetic eruptions from Earth to Mars (Guo et al. 2018; Freiherr von Forstner et al. 2019).

A series of solar storms that occurred in September 2017, while relatively small, comprise the most significant event recorded by RAD and the first to be observed simultaneously at Earth and Mars. Those observations showed that a reduced “Quality Factor” (potential for biological damage) during the event, as well as a post-event Forbush decrease (sweeping away of cosmic rays by the solar wind), resulted in a delivered radiation dose that, unexpectedly, was only slightly greater than that outside of the event (Hassler et al. 2018; Zeitlin et al. 2018; Ehresmann et al. 2018; Guo et al. 2018). Continued observations as the solar cycle progresses are expected to capture larger events and further illuminate these complex interactions.

## Special Techniques and Observations

Curiosity’s science instruments were designed to have the set of capabilities required to achieve mission science objectives. However, unanticipated capabilities have emerged out of creativity and problem-solving. For example, even though the arm-mounted Mars Hand-Lens Imager (MAHLI) camera was designed for close-up imaging of the surface, its ability to focus over a wide range of distances led to its use for inspecting the rover and its wheels (Arvidson et al. 2017a), measuring surface reflectance over multiple emission and phase angles (Liang et al. 2020), and acquiring rover self-portraits. Meanwhile, a joint effort by JPL engineers and the ChemCam team developed the capability for the rover to autonomously select scientifically desirable targets for ChemCam, enabling measurements at new locations without waiting for communication with Earth (Francis et al. 2017). Using ChemCam in passive mode to record solar reflectance spectra of the surface (Johnson et al. 2015), ChemCam’s Remote Micro-Imager for long-distance imaging (Le Mouélic et al. 2015) and the Mars Descent Imager for tracking of surface textures (Minitti et al. 2019) are additional examples.

The versatility of the SAM laboratory has allowed the development and use of experimental strategies and procedures that were unanticipated at launch, including geochronology (Farley et al. 2014; Martin et al. 2017) and “opportunistic” wet chemistry (Sect. 3.2.1). For example, the laboratory was designed with the capability to send gases that evolved from samples over specific temperature ranges to its gas chromatography columns, tunable laser spectrometer, and/or mass spectrometer. It also can cache sample material in internal cups so that experiments can be deferred until mission resources are available, or while testbed runs are completed on Earth. Novel use of the SAM scrubber to remove CO_2_ and H_2_O has improved the detection limit of atmospheric methane by a factor of ∼25.

Scientific and technical results also have been extracted from rover engineering systems that were not designed with such use in mind. Lewis et al. (2019) used Curiosity’s inertial measurement unit (designed for navigating and orienting the rover) to perform the first surface gravity traverse on Mars. Changes in gravitational acceleration as the rover ascended Mount Sharp indicate a lower-than-expected bulk density. In another example, telemetry from the rover’s mobility system, including wheel and suspension motions, rover attitude, and visual tracking of drive progress, reveal how the rover interacts with martian surface materials. This allows estimation of soil properties such as cohesion and shear modulus (Arvidson et al. 2014). Comparison of telemetry with a high-fidelity simulation of the rover’s full mobility system on three-dimensional terrains provided insight into how aspects of the rover, soil, and surface topography (e.g., slope, spacing and height of ripples) affect traction (Arvidson et al. 2017b). Telemetry from the rover’s drill system has been used to estimate the compressive strength of rock targets, yielding clues about the geological processes that have acted on them, including the nature of cements (Peters et al. 2018). These results were enabled through ad hoc collaborations between science team members and project engineers.

The science team also finds opportunities to accomplish scientific objectives that are outside of the mission’s formal scope. A few dozen iron meteorites have been recognized by their distinctive color, morphology, and/or spectra (by Mastcam and ChemCam in passive mode). Active ChemCam measurements on some of these “finds” confirmed their iron-nickel composition and constrain the physical and chemical weathering they have endured (Meslin et al. 2017). Curiosity has periodically observed the martian moons Phobos and Deimos in transit across the sun. The precise measurement of their timings can be used to refine knowledge of their orbits and rates of orbital evolution, which in turn constrain properties of Mars’ interior that control its tidal response (Lemmon et al. 2013). In 2015, when NASA’s STEREO spacecraft were temporarily unavailable, Curiosity’s Mastcam became the only camera in the solar system capable of observing sunspots and incipient solar activity on the “far side” of the sun.

## Rover and Instrument Status

Rover and instrument systems are subjected to environmental stresses from extreme diurnal temperature cycles and radiation each sol on Mars, as well as thermal and mechanical wear from their activities. Yet after eight years, the rover and instruments remain capable of achieving the same quality and breadth of scientific measurements as at the end of the prime mission, with few exceptions. In several cases where degradation has been experienced, the team has developed effective mitigations. Most significant is the recovery of drill-based sampling after the loss of a key motor, as described below. Use of rover and instrument consumables, such as expected mechanism life and pristine sample receptacles, proceed with heightened scrutiny but has not slowed science operations.

At Sol 2844, the top unrealized risks to the mission are the loss of rover flash memory, wheel damage that leads to impaired mobility, loss of the SAM wide-range pumps, and a high-consequence error in commanding. A significant external risk is the loss of a relay orbiter, particularly MRO or Mars Odyssey, given the importance of their consistent afternoon overflights to the pace of MSL operations. The following sections describe the status of key systems in further detail.

### Rover Systems

#### Rover Computer Memory

Extensive analysis after unexpected flight software resets on Sols 2320 and 2339 led the project to conclude that the remaining flash memory on the A-side computer had become unreliable. Half of the flash memory had already been restricted from use after an anomaly on Sol 200 (Vasavada et al. 2014). The project is developing software to make the A-side computer capable of performing cross-string diagnostics in addition to keeping the rover safe indefinitely while the team recovers the (currently fully functional) B-side in the case of an anomaly (update: completed and installed on Sol 2963). The A side is no longer usable for nominal science operations.

#### Wheel Wear

An alarming rate of wheel damage (Arvidson et al. 2017a) was first confirmed after wheel imaging on Sols 488-490. The investigation determined that damage can be exacerbated when, for example, five of the rover’s wheels build up loads within the suspension system that push against a sixth that has encountered a sharp obstacle. The damage rate has been slowed by strategic route planning that avoids terrain assessed to be risky (Arvidson et al. 2017a), as well as careful daily path planning to avoid specific hazards and frequent use of onboard image-based path tracking to reduce positional uncertainty. Furthermore, the sharp, embedded rocks that caused the most damage on the plains are less abundant on Mount Sharp. Traction control software developed to allow the wheels to move independently over obstacles has been in use since Sol 1678 and has successfully reduced internal suspension loads (Toupet et al. 2020).

Regular wheel inspections indicate that Curiosity’s wheels continue to accumulate cracks, punctures, and broken grousers. Damage has been concentrated on the middle wheels and the left front wheel, with the other three wheels showing little damage. Two grousers have broken on the left middle wheel (found on Sol 1641) and one has broken on the right middle wheel (found on Sol 2407). To better characterize the long-term wear and predict life/distance performance, extensive testing was performed at JPL using a wheel and suspension test rig that was driven over analog Gale crater terrains. Test statistics revealed that when three grousers are broken on the same wheel, that wheel has ∼40% of its life remaining. By this metric, at Sol 2844 Curiosity’s wheels have at least 16 km of life remaining, or at least 39 km in total, far exceeding the original design requirement of 20 km.

#### Mechanisms

Several mechanisms on the rover’s mast, arm, turret, mobility system, and high-gain antenna are near or exceeding their design life. In all cases, accumulated use will remain below the durations/cycles used for life testing (usually at least twice the design life) through at least 2022. The drill bit used since landing continues to be effective, as does the brush. On Sol 1231, the CHIMRA (sample processing tool) tunnel motor, used to inspect and clean the 150-um sieve, experienced a stall. The stall has not repeated, but limitations have been placed on the use of the motor, within minimal impact to sampling operations. Mechanical systems and actuators other than those discussed above and below are performing nominally and are expected to exceed their design life.

#### Drill Percussion

Prior to launch, the drill’s percussion mechanism was found to have a flaw that could result in electrical shorts. Although it could not be fixed, rover hardware and software modifications were made to allow rapid detection and response. The mechanism experienced its first short during the transfer of sample from the drill to CHIMRA on Sol 911, halting the operation. After further analysis, onboard fault thresholds were raised to tolerate low-current shorts. The motor controller software also was patched to better protect sensitive electronics and to monitor currents when using percussion. The magnitude and frequency of shorts during subsequent use has been variable, with no trend toward increased degradation. To mitigate the potential future loss of percussion, the project developed the capability to collect drilled samples using a rotary-only mode. A testbed program found that rotary-only drilling makes slow but steady progress for 10-30 mm in Mars analog mudstones and sandstones, but then makes extremely slow progress. In some cases, it does not reach deep enough to collect material in the time available. Rotary-only drilling has since been used successfully on Mars, but its ineffectiveness on more resistant rocks led the project to accept the risk of continuing to use percussion when needed (Sect. 9.2).

#### Drill Feed and Chuck

The drill’s feed motor, used to extend the drill bit into a rock while the arm and turret are stabilized against the surface on fixed posts, experienced a stall on Sol 1536. Diagnostic activities revealed a potentially worsening, once-per-revolution obstruction. Each stall could be recovered through a laborious process, but it became clear that the feed was no longer usable for autonomous drilling. It was permanently moved to a fully extended position to allow the bit to clear the stabilizer posts. A 1.5-year development and test program produced a “feed-extended drilling” technique that relies on the unstabilized robotic arm to control the force on the drill, in lieu of the feed (Sect. 9.2). Fortunately, the loads on the arm are within tolerances, even though it was not designed for such use. The drill chuck motor (used to disengage and exchange drill bits) is functional but shows signs of degradation. The drill feed, chuck, and tunnel motors are the same type and share a vulnerability to the use of a turret vibration mechanism employed to mobilize sample material within the CHIMRA tool. Further use of this mechanism is restricted, which doesn’t affect drill-based sampling (Sect. 9.2) but effectively bars scoop-based sampling.

#### Power System

**Fig. 8:**
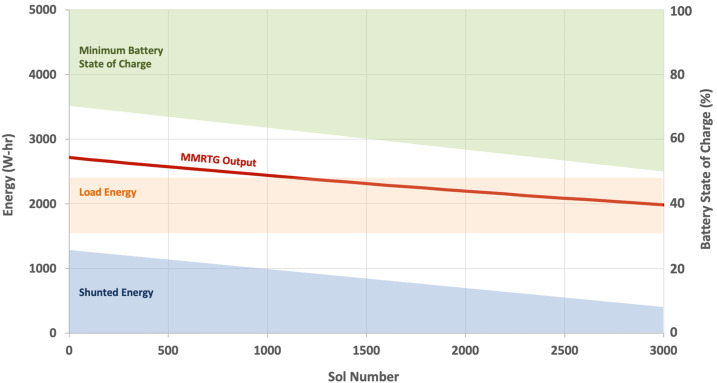
Schematic of rover energy utilization as a function of mission sol. The red line shows the declining MMRTG energy output per sol. The energy used for rover activities (load energy) is typically within the orange region, leaving excess energy to be shunted to the environment as shown by the blue region. The lowest battery state of charge reached each sol falls within the green area. The chart shows that the energy used for rover activities has remained relatively constant. However, as the energy supply and battery capacity have decreased over time, rover activities have discharged the battery to lower levels and left less excess energy. Soon the diminished energy supply and battery capacity will require reductions in energy use and additional time dedicated to battery charging

The output of the rover’s Multi-Mission Radioisotope Thermoelectric Generator (MMRTG) (Fig. 8) continues to decline due to plutonium decay and thermocouple degradation at a rate that agrees with predictions, as does the storage capacity and discharge/recharge rates of the rover’s batteries. The reduction in energy available for science is significant—on Sol 2844 it is only 60% of that at landing—yet it is not expected to notably reduce productivity until 2024 or later. Figure 8 shows that although the energy load due to rover and payload activities has remained relatively constant, the drawdown of the batteries has increased as their capacity has degraded, and the amount of excess energy (shunted away as heat) has decreased as the MMRTG output has declined. In the next few years, the pace of rover operations will become limited by the amount of available energy and by battery recharge times. However, more accurate models of the rover’s heating requirements and science and rover activities, more efficient heater use, as well as increased running of activities in parallel will help to maintain the pace of activities. The battery state-of-charge is kept within limits that are known to optimize battery life.

### Science Instruments

Notable instrument consumables and changes in instrument performance are summarized below. Other instrument components remain fully functional.

A steady and dramatic decrease observed over Sols 799 and 801 in the power output of ChemCam’s autofocus laser was determined to be unrecoverable. A short-term but resource-intensive solution of taking every measurement over a range of focus settings allowed science operations to continue. The ChemCam team developed a final workaround that uses onboard analysis of multiple Remote Micro-Imager images to focus the instrument (Peret et al. 2016). The new method, used since Sol 985, also provides focus to infinity, compared with < 18 m before, allowing long-range imaging with greater efficiency and quality.

The DAN active neutron generator is well beyond its expected lifetime but continues to operate nominally, and scientific use proceeds with the significantly reduced neutron output.

REMS wind sensors on boom 1 were found to be inoperable upon landing. Sensors on boom 2 remained partially operational until Sol ∼1500. Visual inspection did not have sufficient resolution to confirm physical damage from landing debris and/or blowing sand as the suspected cause in either case.

On Sol 887, the SAM team detected a partial obstruction in the transfer line to the 5th (MXT-CLP) gas chromatograph column. The team believes that volatiles from a previously run experiment saturated available pore spaces in the injection trap, enhanced by the degradation of the adsorbent material from thermal cycling. The team has switched to using another column with similar capability. If necessary, they may attempt to clear the trap with flash heating.

Both of the partially redundant SAM wide-range pumps have exceeded their design life with no indication of lube or bearing degradation. Losing one pump would extend the duration of each solid sample experiment. A qualification model was successfully tested to twice the design life.

## Performance of Curiosity’s Sampling System

Curiosity’s sampling system was designed to acquire soil and powdered rock with a scoop and rotary-percussive drill, respectively, to sieve the material to the range of particle sizes that meet instrument requirements, to divide the material into portions, and to deliver the material to the Chemistry and Mineralogy (CheMin) and SAM instrument inlets, to a sample analysis tray, or to the ground for inspection by remote or contact science instruments (Anderson et al. 2012, 2015; Helmick et al. 2013). The system has enabled analyses of 33 samples of loose aeolian material, mudstones, and sandstones through Sol 2844, including twelve after recovering from the drill feed anomaly.

### Scoop-Based Sampling

Eight of nine scooping attempts have been successful (Table 2). The Gobabeb 3 attempt was aborted after the stall of a motor within the CHIMRA (Sect. 8.1). Material from six of those eight scoops was processed and delivered to at least one instrument. The two not delivered include Rocknest, used only to dilute terrestrial contamination on CHIMRA interior surfaces, and Rocknest 2, discarded when foreign object debris was observed near the collection site (Anderson et al. 2015). CHIMRA was designed to sieve material into one of two particle size ranges prior to delivery: <150 μm or <1 mm. During the Gobabeb campaign, sampling engineers were able to use both sieves to create a distribution between 150 μm and 1 mm, improving the science team’s ability to understand how composition varies with particle size.

**Table 2:** Sample collection attempts via scooping and processing notes relevant for sample analyses. The unsuccessful attempt is listed in parentheses

Target name	Sol	Notes and processing path
Rocknest	61	Decontamination #1; not delivered
Rocknest 2	66	Discarded due to foreign object; not delivered
Rocknest 3	69	Decontamination #2; <150 μm
Rocknest 4	74	Decontamination #3; <150 μm
Rocknest 5	93	<150 μm
Gobabeb	1224	<150 μm
Gobabeb 2	1228	<1 mm with enrichment
(Gobabeb 3)	1231	<1 mm with enrichment; tunnel motor stall
Ogunquit Beach	1651	<150 μm

### Drill-Based Sampling

**Fig. 9:**
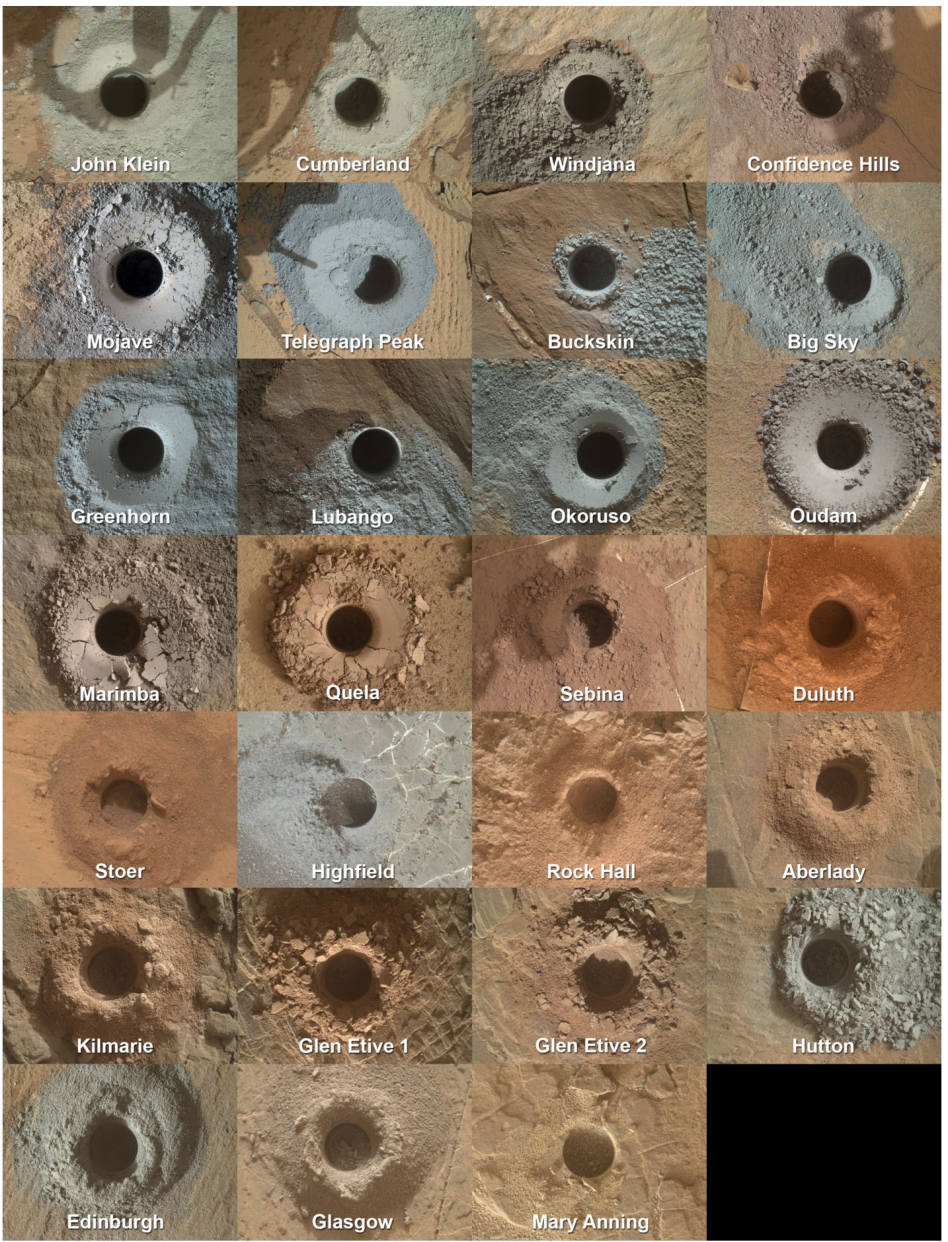
Gallery of MAHLI images of the 27 holes successful drilled (see Table 3 for details) through Sol 2844 (image credit: NASA/JPL-Caltech/MSSS). The diameter of each drill hole is ∼16 mm. In most cases the surface is coated by typical martian dust and the color of the drill tailings reveals the different composition and spectral properties of the rock interior

Curiosity successfully drilled and analyzed material from 27 full-depth holes (Fig. 9) through Sol 2844 (Abbey et al. 2019, 2020). Nine attempts were unsuccessful (Table 3). Before each drilling attempt, the team assesses the rover’s stability against the forces and dynamics of drilling, the safety of the drill as it interacts with the target, the risk that drilling will displace or fracture the rock and remove the pressure needed to force cuttings into the drill stem, and the possibility that the powdered material might clog or stick to hardware based on its chemical composition. Abbey et al. (2019) discusses drilling requirements and triage in greater detail. Approval for drilling and sample collection comes from a group of engineers, scientists, and project management. Given the prevalence of small blocks and highly fractured outcrops, on several occasions the team has chosen to accept that drilling or sample acquisition may not successfully complete, as long as risk to hardware is ruled out.

**Table 3:** Sample collection attempts via drilling and notes relevant for sample analyses. Attempts that failed to collect sample material are listed in parentheses

Target name	Test (Sol)	Full (Sol)	Achieved depth (mm)	Notes
John Klein	180	182	63.9	
Cumberland	–	279	66.3	
Windjana	615	621	65.1	
(Bonanza King)	724	–	–	Target unstable
Confidence Hills	756	759	65.8	
(Mojave)	867	–	–	Target fractured
Mojave 2	881	882	65.0	
Telegraph Peak	–	908	65.9	
Buckskin	1059	1060	64.5	
Big Sky	1116	1119	64.4	
Greenhorn	1134	1137	65.3	
Lubango	–	1320	65.3	
Okoruso	–	1332	65.1	
Oudam	–	1361	65.3	
(Marimba)	–	1420	4.6	Low rate of penetration
Marimba 2	–	1422	65.2	
Quela	–	1464	63.9	
Sebina	–	1495	65.4	
(Precipice)	–	1536	0.0	Rotary only; drill feed stall
(Lake Orcadie)	–	1977	9.8	Rotary only; low rate of penetration
(Lake Orcadie 2)	–	1982	2.1	Rotary only; low rate of penetration
Duluth	–	2057	57.4	
(Voyageurs)	–	2112	4.2	Low rate of penetration
(Ailsa Craig)	–	2122	4.8	Low rate of penetration
Stoer	–	2136	48.8	
(Inverness)	–	2170	5.6	Low rate of penetration
Highfield	–	2224	48.0	
Rock Hall	–	2261	43.3	
Aberlady	–	2370	42.9	Target fractured
Kilmarie	–	2384	44.2	
Glen Etive	–	2486	45.2	
Glen Etive 2	–	2527	42.6	
Hutton	–	2668	43.3	
Edinburgh	–	2711	26.2	Low rate of penetration
Glasgow	–	2754	46.9	
Mary Anning	–	2838	46.9	

After displacing the Bonanza King target and fracturing the Mojave target, the drilling protocol was revised to reduce the use and magnitude of percussion (Peters et al. 2018). Later concern over the degradation of the percussion mechanism led the project to develop a rotary-only drilling method (Abbey et al. 2020), baselined for use starting with the Precipice target. Persistent issues with the drill’s feed motor, first noticed when it stalled at Precipice, instead forced a major redesign of drill-based sampling. The investigation of the stall determined that drill’s feed motor was no longer reliable (Sect. 8.1). This affected not only drilling, but also sample processing, since it is necessary to retract the feed in order to move collected sample material into CHIMRA. If the feed motor were to permanently fail in the retracted state, the drill would no longer be able to extend beyond its stabilizer posts and access the surface. In response, the project developed new sampling methods that keep the feed fully extended, called Feed-Extended Drilling (FED) and Feed-Extended Sample Transfer (FEST).

**Fig. 10:**
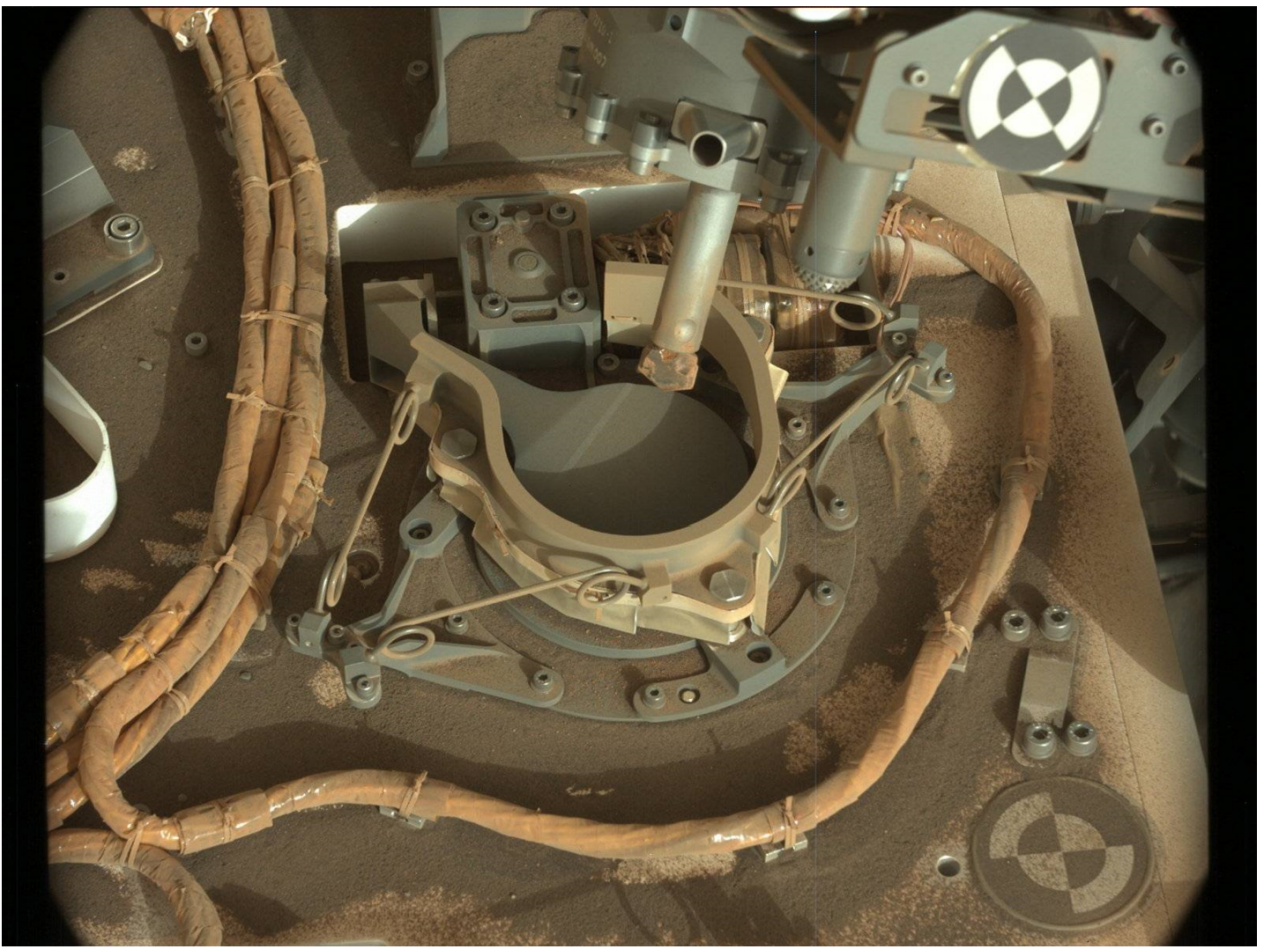
Sol 2068 Mastcam image showing the rover’s drill bit positioned over the closed inlet to the CheMin instrument. After the loss of the drill’s feed motor, sample material must be delivered directly from the drill to the CheMin and SAM instrument inlets. Prior to the loss, sample material was processed and delivered by a dedicated tool (ML010980, image credit: NASA/JPL-Caltech/MSSS)

In FEST, material within the drill stem is portioned directly into the instrument inlets (Fig. 10) using small pulses of rotation and percussion. FEST portions differ in three significant ways from those processed and delivered by CHIMRA. First, without sieving there is no way to guard against larger particles that could obstruct the 1-mm instrument inlet screens or clog other pathways. Fortunately, testbed analyses and experience on Mars show that the drill creates particles that are 10s of micrometers or smaller. Second, FEST portions are smaller and more variable in mass than those created by CHIMRA. Neither CheMin nor SAM was designed to quantify delivered portion mass, but it can be measured in the testbed and estimated from images of test portions on Mars. During each FED/FEST campaign, portions are delivered to the workspace and/or closed instrument inlet covers for characterization. Statistics from these tests are used to determine how many portions to deliver to each instrument as well as the total number of portions likely to be in the drill stem. Third, early use of FEST on Mars revealed that a significant fraction of each portion may fail to enter the instrument inlet due to deflection from wind and/or the horizontal velocity imparted by the drill bit. Some SAM analyses are consistent with low portion mass. Recent efforts to safely lower the drop-off height by bringing the drill even closer to the instrument inlets appear to be successful at reducing loss from deflection.

Unsuccessful drill attempts (Table 3) were a result of target instability, fracturing, the drill feed anomaly, and resistance of the target to drilling. Resistance to drilling was a problem especially on Vera Rubin ridge, likely due to more challenging rock properties. An initial, non-percussive version of FED failed to reach sampling depth in two attempts at Lake Orcadie. Subsequently, drilling algorithms were adjusted to start with non-percussive drilling and use moderate percussion as needed. When percussive FED also had difficulty penetrating the rocks of the ridge, the project accepted the risk of using the highest percussion levels when needed. To reduce the duration of percussion, commanded hole depth was reduced from 65 mm to 50 mm at Duluth, 45 mm at Voyageurs through Highfield, and 40 mm afterward. Although the attempt at Edinburgh did not reach full depth, it did pass ∼20 mm, the depth at which the drill begins ingesting sample material. In spite of the shallow depth, both CheMin and SAM received sufficient material for successful analyses.

## Mission Operations, Performance, and Management Principles

### Operations Process

The MSL operations process enables the use of a highly sophisticated robotic system to address complex and diverse scientific questions. It succeeds to the extent that scientific objectives can be defined, prioritized, and accurately communicated to technical experts (e.g., roboticists and systems engineers) who translate the objectives into command sequences. It also attempts to optimize for science return among a myriad of constraints that include rover resources and safety, the martian environment, telecommunication, and the limitations of the software, hardware, and processes involved. Finally, the rover is a shared resource: a variety of science and engineering activities must divide the available energy, time, and transmitted data volume, and must coordinate the use of the rover’s mast, arm, sampling, and mobility systems.

**Fig. 11:**
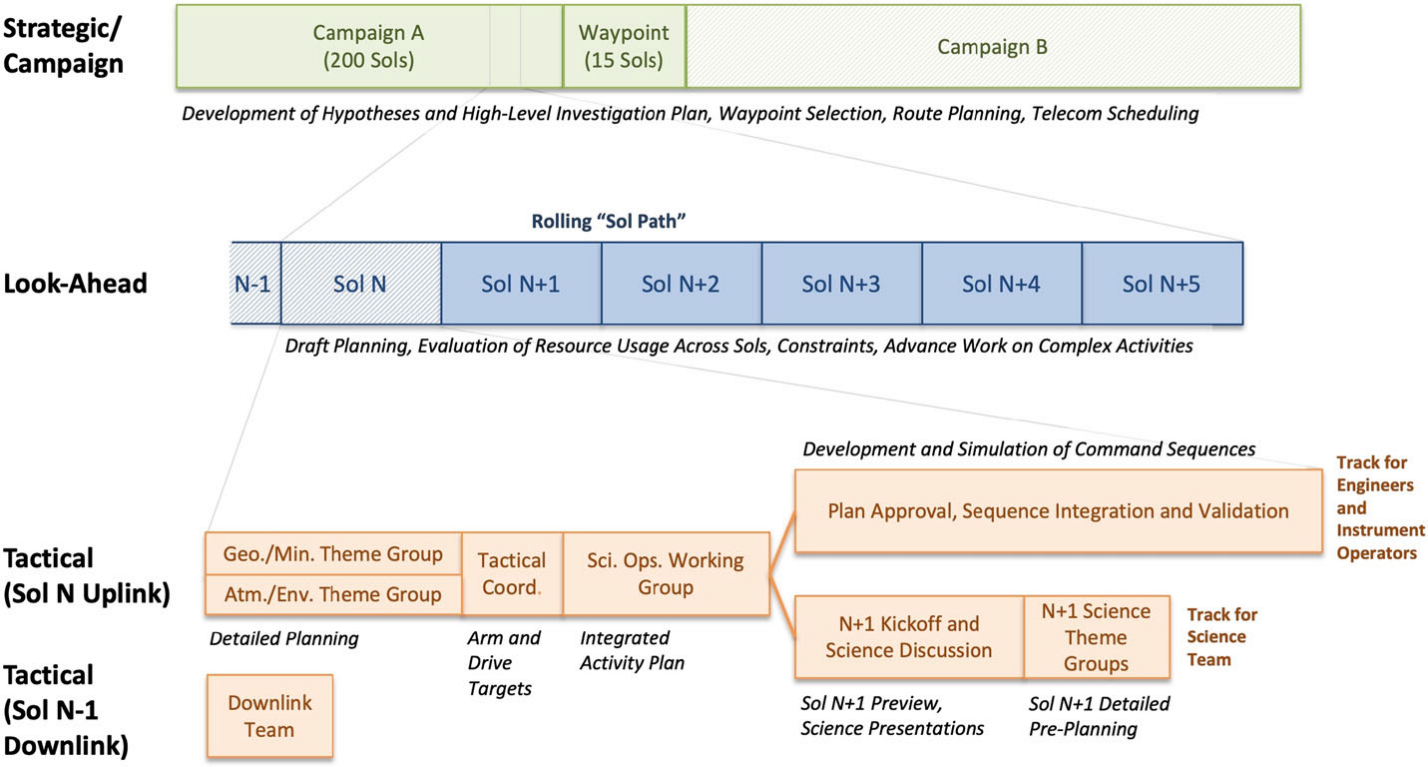
Schematic and descriptions of science operations planning tiers. MSL science operations involve planning over three time domains: Strategic (10s to 100s of sols), Look-Ahead (2-10 sols after the current uplink), and Tactical (next uplink, referred to as the “Sol N” plan). The steps shown in the Tactical row occur over a single shift in the order shown. Hashed areas are time periods that are not the primary focus of the planning tier but provide initial conditions or future constraints

Missions involving landed spacecraft are by nature intensely interactive; time is of the essence as the team reviews the latest telemetry and measurements and responds with the next plan. In the midst of this daily pressure, the team also must monitor progress toward high-level mission objectives and manage the draw-down of consumable resources. Recognizing the inherent complexity of the endeavor, Curiosity’s operations process includes long-term (strategic), near-term (look-ahead planning), daily (tactical), and supporting components (Fig. 11). Below we update and expand on the descriptions of Vasavada et al. (2014) and Chattopadhyay et al. (2014).

#### Strategic Planning

The strategic planning process covers the next 10s to 100s of sols. It seeks to develop an overarching science investigation plan for each region by defining hypotheses, measurement objectives, and sites of interest, as well as by constructing a traverse route through those sites in coordination with rover mobility experts. The science team may set a calendar goal for completing a regional investigation, called a campaign, but no longer creates sol-by-sol strategic plans as was done early in the mission. Strategic plans are occasionally re-assessed in response to discoveries or changes in system capability or mission performance. Strategic planning also includes negotiations with the Deep Space Network for uplink sessions and with the relay orbiters teams for downlink opportunities.

#### Look-Ahead Planning

Each day of tactical planning (the Sol N plan), anticipated rover and instrument activities (specific activities or placeholders) are scheduled into a rolling sol-by-sol plan covering the following 2-10 sols (sol N+1 and following) that passes initial checks against available time, energy, and data volume, timing of telecom sessions, interdependencies, and safety constraints. The resulting “sol path” integrates near-term measurement objectives from the science team as well as engineering activities such as routine maintenance or checkouts of new capabilities. As part of look-ahead planning, rover drivers refine upcoming routes toward science targets and assess their accessibility to the arm. New or logistically complex activities are developed and documented to guide later tactical implementation. All this advance work enables the tactical team to execute plans with higher complexity and science quality than would otherwise be possible. However, the operations process also is designed to allow the tactical process to rapidly adjust in response to discovery or anomaly without advance planning, although potentially with reduced complexity.

#### Tactical Planning

A core requirement of MSL operations is to be able to assess downlink as well as plan up to three sols of rover activities within a single shift. A downlink team consisting of rover and instrument specialists verifies the success of previous plans, identifies any issues, and updates resource estimates. On the uplink side, a lead scientist, sub-leads for geology and environmental monitoring, instrument specialists, and additional science team members work closely with a team of rover robotic operators to choose targets and plan observations and rover motions. Subsequently, systems engineers integrate instrument and rover activities, model rover resource usage, review plans and rover simulations for safety, and create and validate command sequences for uplink to the rover.

The operations process allows for the timely transfer of information between the strategic, look-ahead, and tactical planning components. For example, strategic plans are assessed at biannual science team meetings, but a campaign (strategic) working group meets once a week to monitor progress and feed look-ahead planning. Look-ahead and tactical roles work the same shift and share information throughout the day. The JPL-based roles are co-located in adjoining rooms. Look-ahead planners provide context and assess near-term impacts of tactical options (e.g., any downstream effects of using more energy) in real time. They also rapidly adjust upcoming plans, drives, and targets in response to tactical outcomes. In 2015 the project created a new role, the Science Operations Coordinator (SOC), intended to be staffed continually by a single JPL-based individual (with alternates used rarely). The SOC role was created in response to the need to better facilitate communication between the science and engineering teams and between supratactical and tactical planning, as well as to provide a “long-term memory” given that the day-to-day staffing of most other roles cycles through a pool of personnel, often with different people staffing key roles each day.

#### Supporting Activities

Management and engineering teams continually work to improve operations efficiency, upgrade mission tools and capabilities, and train new personnel. Rover and instrument engineering teams review performance trends, investigate anomalies, and develop mitigations and workarounds to ensure the continuity of key capabilities. The rover’s route and scientific targets are continuously updated in a Geographic Information System database in order to provide a common reference across the project. Science team members use instrument testbeds and laboratory studies to improve measurement techniques and instrument capabilities, and to aid interpretation of results from Mars. The project maintains a high-fidelity rover testbed at JPL that continues to be critical and in constant demand for investigating and mitigating hardware and software anomalies (Sect. 8), including the restoration of drilling, and for developing advanced rover/instrument capabilities and software updates.

### Overall Mission Performance

The value of the MSL mission ultimately is tied to its scientific contributions and technological achievements. In a formal sense, the mission met its NASA-defined success criteria in its Prime Mission and has demonstrated most of the functional requirements that guided its design (Online Resource 2). However, it’s also important to assess how efficiently the mission’s resources were used and what factors limited productivity. We first consider the constraints that exist on Mars, and then those that exist on Earth.

What the rover can accomplish on a given sol may be limited by time on Mars, downlink data volume, or energy. Some rover and instrument activities are limited to specific local time ranges for scientific or thermal reasons. Activities that must be downlinked before the next planning shift begins on Earth have a particularly restricted time period during which they must occur on Mars. The window between the receipt of new commands by the rover and this downlink can be as short as four hours. Plans also may be constrained when the data volume required to inform the next planning cycle exceeds the capacity of the relay passes. Energy becomes a key constraint when activities are energy intensive, when the rover’s batteries are not able to recharge between plans, or when a minimum charge limit is reached. Whether time, data volume, or energy is the driving factor on a given sol is determined by the plan’s content (and that of neighboring plans), relay orbiter timings and data volumes, and the gradual decline of the rover’s power source. The team continually works to mitigate these constraints by developing ways to use resources more efficiently. Because of the importance of low-latency data to mission progress, the availability and capacity of afternoon relay communication passes, most reliably from orbiters in sun-synchronous orbits, has proven to be more of a constraint than the total downlink capacity from all relay passes.

Productivity also is limited by the logistics and capacity of the science operations team, such as the timing and pace of planning shifts, the complexity of command loads, and the time needed for scientific analyses in order to make decisions. For a number of years, the team worked an 8- to 10-hour shift that could slide up to -2 or +3.5 hours past a nominal start time in order to partially track the phasing of Earth and Mars local times over their 5-week cycle. A shift was scheduled only when this phasing allowed data from the prior plan to be received by the start of the shift, and only on weekdays. In this scenario, operations were roughly split between 5 days/week and 3 days/week each ∼5-week period, for a total of ∼16 shifts/month. This can be compared with the maximum possible 29-30 shifts/month if the team worked on “Mars time” and weekends. The rate of ∼16 shifts/month leaves much untapped productivity, but is driven by funding and sustainability (human factors). The shift rate and duration used since the start of EM3 in October 2019 (∼14 shifts/month and up to a +2 hour slide) results from available funding. Within a few years, however, the decreased output of the MMRTG (Sect. 8.1) will require dedicating sols to recharging the battery, and 12 shifts/month (e.g., Monday, Wednesday, Friday) will suffice.

The scope of each command load is intentionally capped to ensure that the operations team can complete it. Fortunately, the team’s experience now allows complex plans to be completed in significantly less time than earlier in the mission. The pace of operations also may be deliberately limited to allow time for scientific analysis. A common example is when the result of one CheMin or SAM analysis informs the next. Finally, a myriad of other constraints affect productivity in small or infrequent ways, such as limitations on operability (e.g., allowable temperatures, not pointing ChemCam at the sun), maintenance activities, challenges encountered on Mars (e.g., slippery terrain, wind, target illumination), weekends, holidays, solar conjunction (no commanding possible), safety-driven faults, and anomalies with the rover, instruments, operations infrastructure, relay orbiters, or the Deep Space Network (DSN). Constant vigilance and proactive effort are required to maximize the rover’s productivity in light of the steady stream of small issues that, when combined and integrated over time, would significantly hinder it.

In terms of metrics, the eight Earth years after landing contained 280, 247, 187, 201, 188, 190, 184, and 159 command cycles, respectively. The first two years included some Mars time and weekend shifts. Subsequent years were planned at ∼16 shifts/month until the eighth year, which was planned at ∼14 shifts/month. The team sent commands on 1636 of 2844 sols (58%). Seventy-one sols (2.5%) were *completely* lost due to anomalies, with about half from rover-related issues, 23 from DSN-related issues, three while the JPL-based team rapidly transitioned to remote operations during the COVID-19 outbreak, and a few each from instruments and operations process issues. Science activities on a few hundred sols were *partially* lost due to command, instrument, or rover issues during execution, or because resources were dedicated to recovering from prior anomalies. These include cases in which the martian environment presented a challenge that was beyond the team’s ability to anticipate, such as excessive slip in a drive or fracturing of a rock during drilling, triggering the rover’s fault response to ensure its safety. There were drives on 780 sols (27%) with an average length of 30 m. Over the first seven years, about 10% and 8% of drives were terminated early by mobility fault protection and non-mobility causes, respectively, but the rover achieved 91.7% of commanded distance (Rankin et al. 2021).

### Guiding Management Principles

Over the course of the mission, MSL project management has been guided by a number of principles that have helped to maximize the mission’s productivity and the quality of its scientific results within available resources. They also serve as “lessons learned” for future missions that have operational complexities similar to those described above.

#### Responsive and Flexible Tactical Operations

Planetary rover missions are intended to allow scientists and engineers to interactively use the spacecraft to explore a field area. It follows that the productivity of the mission is highly correlated with the frequency of opportunities for scientists and engineers to receive the latest information from the rover and respond with commands. The caliber of those interactions also matters, such as the volume of new information enabled by the communications infrastructure and the level of planning complexity that can be achieved by the operations system in the time available. Curiosity’s tactical operations process strives to maintain a high level of both responsiveness and flexibility, as described in Sect. 10.1.

Responsiveness and flexibility in tactical planning are strong drivers of the mission’s cost. In simple terms, the mission cost is a combination of the cost per planning cycle (i.e., staffing of operations shifts) and the fixed costs from strategic and supporting work (Sect. 10.1.4). In theory, savings could be realized if there were fewer tactical shifts, if coordination with the Deep Space Network and relay orbiters were relaxed, or if commanding were constrained to follow generic templates that could be planned and approved in advance. While the project has found numerous ways to reduce cost, it has avoided such changes that would limit the science team’s ability to interactively use rover and payload capabilities in the most appropriate ways for a given target, and as a result, significantly reduce the value of the measurements.

#### Mission Time Management Follows Science Strategy

The mission’s time, both over the scale of sols and over the mission’s life, is managed with a view to completing an efficient but comprehensive scientific investigation of the rover’s extensive field area. The foundation of this time management is strategic planning. At the highest level, the mission’s strategy is to ensure that the rover will reach all key areas of interest in the foothills of Mount Sharp while it still has the capabilities needed to investigate them. The mission follows a strategic traverse route that optimizes for science value, rover safety, and efficiency. Continuity of observations between key areas of interest (i.e., not skipping ahead) also is a priority, given the criticality of measuring textural and compositional trends to the understanding of the geologic history of Mount Sharp. The tension between the breadth and depth of observations, coupled with the finite rover lifetime, is constantly on the minds of team members.

Mission time is (sometimes ruthlessly) managed to sustain forward progress while maintaining at least a minimum depth and continuity to the rover’s science investigations. Once the team has decided to investigate a particular site, the rover remains there just long enough to capture what the team determines to be the highest-value observations. Unless the rover is executing a multi-sol campaign objective or drilling, the typical practice is to insert a drive in every tactical plan (after a few hours or 1-2 days of remote and contact science on weekday or weekend plans, respectively). Additional time may be needed to assess these data and realize their significance, however. The team therefore accepts the cost of occasionally sending the rover back to a recent site. Sometimes multiple passes are deliberately planned through a key area, with the observations in each pass benefiting from a more mature scientific understanding than the previous one (e.g., Yingst et al. 2016). Such strategies generally have produced an acceptable balance between time and depth of study, but naturally there are parts of the traverse that, in hindsight, were too rushed or too prolonged.

**Fig. 12:**
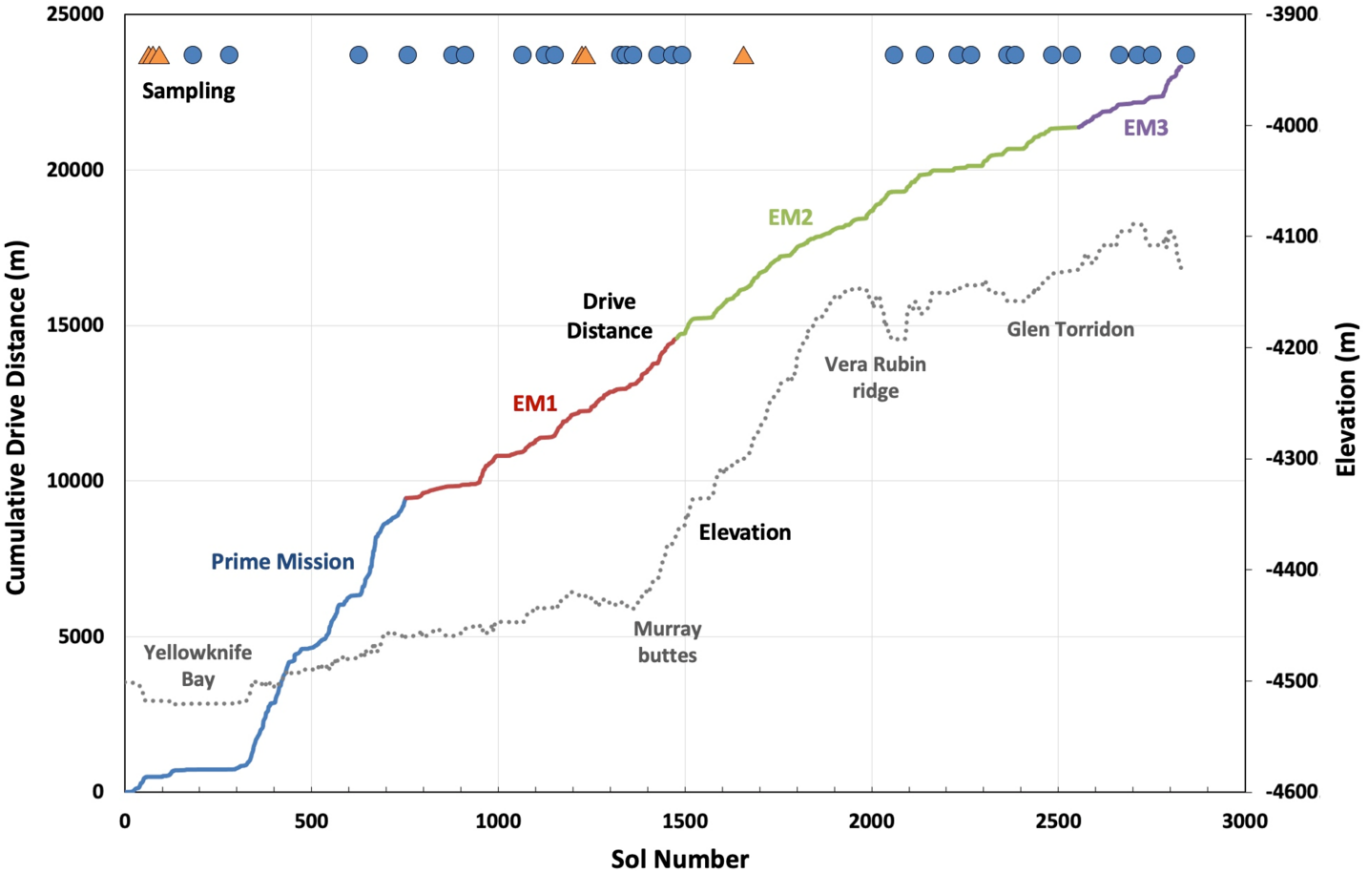
Summary chart of mission performance showing cumulative drive distance (solid line) and elevation (dotted line) as a function of sol and mission phase. After a sprint to reach Mount Sharp in the latter half of the 26-month Prime Mission, the pace of driving (a proxy for total rover activity) remained relatively constant in EM1 (two years) and much of EM2 (three years). The pace subsequently has slightly slowed, with the exception of a sprint near the end of the period shown. The elevation trend shows the descent into Yellowknife Bay in the early Prime Mission, the direct climb up Mount Sharp to the Vera Rubin ridge from mid-EM1 to mid-EM2, and the more gradual ascent through the Glen Torridon region. Orange triangles and blue dots mark the timings of successful scooping and drilling campaigns, respectively

The mission strategy also acknowledges that it must be adaptable to scientific discoveries and unforeseen technical or budgetary issues. Accomplishing mission science objectives takes precedence over reaching particular distance or elevation milestones. For example, the rover was expected to reach and explore Mount Sharp during its Prime Mission, but instead it remained on the plains. This delay was largely driven by the discovery of a region (Yellowknife Bay) where the mission’s prime objectives could be (and were completely) addressed. Total time spent was exacerbated by major technical issues with the rover’s memory and wheels, as well as overly optimistic timelines for the early mission (Sect. 5.3 of Vasavada et al. 2014). In its second year, the mission used a faster pace of driving (Fig. 12) to make up time as described in Online Resource 1. In EM1 and EM2, the predicted timelines for reaching specific areas on Mount Sharp were again lengthened in response to unexpectedly rich scientific results, particularly discoveries of additional types of habitable environments. At each step, the mission team acknowledged the added risk to later scientific targets. However, these deferrals also were opportunities to re-amortize the mission’s lifetime over its expected field area, incorporating the latest predictions of the rover’s health and mission resources. In EM3, the mission has a greater focus on reaching key remaining areas by fixed dates, in light of the diminishing output of the rover’s power system, as well as increasing technical and funding concerns.

#### An Integrated and Inclusive Team Culture Increases Science Return

The MSL mission is driven by science but enabled by technology and engineering. The project therefore has prioritized communication and relationship-building between scientists and engineers at all stages, from development (Vasavada 2009) through operations. Mission scientists can optimally design and operate their instruments if they understand the capabilities and constraints of the rover’s systems. Similarly, nuanced discussions of mission science objectives, geological materials and processes, and the martian environment will result in rover design and operations choices that produce higher science return. Reaching a state in which all project contributors share a common understanding, motivation, and criteria for measuring success is not automatic; it must be modeled and reinforced by project leaders.

MSL science operations are structured to share decision-making among science and engineering representatives and to base decisions on inputs from both groups. The project strives to use this model at all levels, from the geologist working interactively with a robotics engineer on how best to place an instrument on a rock target, to the Project Manager and Project Scientist jointly making decisions about the rover’s risk posture or project resources.

MSL has been described as having the organizational complexity of a flagship orbiter (i.e., independently selected, PI-led instrument investigations and Participating Scientists), but with the pace and team synchronization required of a rover. For example, the payload instruments, while selected separately, provide complementary measurements that together accomplish the overall habitability goal. Furthermore, all instruments and investigations are affected by daily operations decisions (e.g., where to place the arm or drive). Therefore the MSL science team operates both as a single team and as PI-led investigation teams. Mission PIs meet regularly with the Project Scientist to oversee the mission’s science, and all have agreed to make decisions as a team, primarily through representatives that are staffed in operations roles. Most decisions involving science strategy and instrument/investigation resource usage are made in teamwide discussions. To aid decision-making, recommendations are prepared by Long-Term Planners, campaign leaders, path planners, instrument teams, or individual team members.

The MSL mission actively promotes early career engineers and scientists to lead the resolution of technical issues and organize scientific plans and analyses. Several major investigation campaigns have been facilitated by postdoc-level scientists, benefiting the team with their energy and devotion, and providing them with valuable mission leadership experience. The benefits to NASA are widespread, but are most evident in the Mars 2020 mission, in which many key roles are staffed by engineers and scientists trained by MSL. In the coming years, additional emphasis will be placed on widening diversity in team membership, primarily through the students and post-docs that rotate through the team, and in leadership, as long-serving project and instrument team leaders are encouraged to plan for succession.

## Future Plans and Feed Forward

### Plans for the Remainder of EM3 and Beyond

Curiosity’s third extended mission (EM3) began October 1, 2019 (Sol 2544), when the rover was partway through its investigation of Glen Torridon. EM3 focuses on understanding the persistence of water and further diversity of habitable environments in Gale crater. The traverse planned for the remainder of this three-year extension would significantly increase the thickness of strata on Mount Sharp explored by the rover, likewise extending the temporal record of ancient environments. It spans the transition from predominantly clay-bearing to hydrated magnesium sulfate-bearing strata that is hypothesized to record a local and possibly global desiccation.

Clay-sulfate transitions are observed widely on Mars from orbit and may have resulted from a transition to a more arid climate (Bibring et al. 2006; Milliken et al. 2010; Grotzinger and Milliken 2012; Ehlmann and Edwards 2014; McLennan et al. 2019). EM3 and later extended missions provide the first opportunity for any mission to examine and document a clay-sulfate transition on the ground. Is it associated with the end of long-lived lakes within Gale? Are the sulfates authigenic, diagenetic, or associated with reworked particles, among other possibilities? What might explain the presence and dominance of magnesium sulfates with different hydration states, as interpreted from MRO-CRISM data (Sheppard et al. 2021)? The detailed study of the sulfate mineral assemblages will expand our understanding of the range of habitable environments that existed at the surface and subsurface, and how different environmental conditions affect the preservation of organic molecules.

Curiosity also will examine younger landforms that are central to understanding the record of younger habitable environments in Gale. These include further study of the Greenheugh pediment (Sect. 2.3) as well as Gediz Vallis, a wide canyon that cuts through the sulfate-bearing unit, and the stratified Gediz Vallis ridge that is hypothesized to contain a record of mass flow, and possibly fluvial or deltaic environments (Palucis et al. 2016). Running along the floor of Gediz Vallis above the ridge is a possible fluvial channel, an intriguing feature as it represents a chance to study the youngest water-carved landform that the rover can access, and may contain debris delivered from higher on Mount Sharp. These features record a period of wind- and water-driven erosion and deposition that occurred after the clay- and sulfate-bearing units were deposited, buried, lithified, and exhumed.

Through EM3 and beyond, Curiosity will extend its measurements of atmospheric composition and dynamics, cycles of water vapor and dust, and the radiation environment by additional Mars years. The rover will traverse to higher elevations and closer to the center of the crater, allowing further assessment of the influence of the crater and Mount Sharp on the meteorology and the mixing of air between the crater interior and surroundings (Rafkin et al. 2016). Compositional measurements will attempt to further constrain the processes driving the variable methane abundance, as well as the factors that control the abundance and cycling of atmospheric water vapor and oxygen. Ongoing measurements of the radiation environment will witness the recovery from the deep solar minimum of 2019-2020, providing novel information about how the solar cycle affects the composition, energy, and dose rates that would be experienced by future human explorers.

### Broader Impacts on Mars and Planetary Exploration

Curiosity’s assessments of paleoenvironmental conditions, habitability, and preservation of organic molecules were designed to precede NASA’s commitment to return and analyze samples from Mars. The mission’s results indicate that ancient Mars offered habitats for potential life and increase confidence that ancient biosignatures are capable of being preserved in rock samples. The selection of Jezero crater as the landing site for the Perseverance rover is a testament to the success of the MSL strategy of exploring an ancient fluvial-lacustrine system. Lessons learned by MSL in conducting robotic geological and sampling investigations, co-interpreting orbiter and in situ measurements, and observing a diversity of atmospheric phenomena from a surface station will increase the return from Perseverance and future missions.

The MSL mission has engaged millions of people around the world through the stories of its ambition, challenges, and discoveries via its web sites, social media, press releases, image and video products, countless public talks, and a blog updated daily by science team members. Perhaps the mission’s most tangible legacy and future impact are the thousands of engineers and scientists, including hundreds of students and postdocs, that have participated in its design and operation, and in the analyses of returned data.

## Supplementary Information

Below are the links to the electronic supplementary material.

Mars Science Laboratory Mission Narrative through Sol 2844 (Eight Earth Years) (PDF 4.8 MB)

Mission Science Objectives, Functional Requirements, and Success Criteria (PDF 69 kB)

Summary Table of Rover Traverses (PDF 241 kB)

## Data Availability

NASA Planetary Data System.

## Materials Availability

N/A.

## Code Availability

N/A.
