# Supplementary material for: Mission Overview and Scientific Contributions from the Mars Science Laboratory Curiosity Rover After Eight Years of Surface Operations
Source: Space Sci Rev. 2022 Apr 5;218(3):14. doi: 10.1007/s11214-022-00882-7 (PMC8981195; doi:10.1007/s11214-022-00882-7)

ONLINE RESOURCE 1

Mission Overview and Scientific Contributions from the Mars Science Laboratory Curiosity Rover After Eight Years of Surface Operations

Space Science Reviews

Ashwin R. Vasavada

Jet Propulsion Laboratory, California Institute of Technology, Pasadena, California, USA

Email: ashwin.r.vasavada@jpl.nasa.gov

Mars Science Laboratory Mission Narrative through Sol 2844 (Eight Earth Years)

This document contains a detailed narrative of the Mars Science Laboratory (MSL) mission from launch through Sol 2844. It is intended to be a contextual reference for those studying the mission or using its scientific data by recounting mission events, scientific decisions and strategies, major rover and instrument activities, and place names. It describes scientific findings only to the extent needed to support the narrative. Additional contextual information including weekly mission status reports, daily downlink and uplink planning reports, and a table of activities by sol can be found in the Curiosity (MSL) Analyst's Notebook at the NASA Planetary Data System's Geosciences Node (https://an.rsl.wustl.edu/msl). Mission press releases and a daily science operations blog can be found on the mission's web site (https://mars.nasa.gov/msl/).

Table 1 provides a conversion between the Mars sol number and the Earth date of mission milestones.

1. Launch through Landing

1.1 Launch and Cruise Activities

On November 26, 2011, at 10:02 A.M. Eastern Standard Time, the MSL spacecraft lifted off from Launch Complex 41 at Cape Canaveral Air Force Station aboard a United Launch Alliance Atlas V (541) rocket. About 44 minutes later, after a burn to reach a parking orbit, a coast phase, and an additional burn and spin-up, the MSL spacecraft separated from the Centaur upper stage and began its interplanetary trajectory. Launch occurred at the beginning of the daily launch window and on the first attempt, though on the second day of the 24-day launch period due to a one-day delay to service the rocket that was announced a week earlier. Six minutes after separation, the Deep Space Network station at Canberra, Australia gained direct communication with MSL and received a positive health status report. Abilleira (2013) provides an overview of trajectory and performance from launch through landing.

Numerous engineering checkouts and maintenance activities occurred throughout cruise. The operations team also addressed two post-launch anomalies. The catalyst beds of a number of the cruise stage thrusters were found to be warmer than expected, so use of heaters was reduced to keep the beds at intended temperatures. On November 29, 2011, the spacecraft experienced a reset of its prime (A-side) computer as it attempted its first transition to celestial guidance (star tracking). The reset put the spacecraft briefly into "safe mode." Further analysis revealed a bug in the memory management of the computer which was subsequently patched, avoiding future resets that could have interrupted critical events.

Curiosity's science mission began on December 6, 2011, when the RAD instrument conducted a post-launch checkout and began measuring energetic particle radiation. It operated throughout most of the cruise phase before powering off for landing preparations on July 14, 2012. The only other regular payload activity was a weekly decontamination

heating of the ChemCam spectrometer to reduce the build-up of volatile contaminants on its optics.

A highly accurate injection by the launch vehicle allowed the first planned Trajectory Correction Maneuver (TCM) to be postponed from early December to January 11, 2012, in order to allow the team to focus on the memory anomaly. Execution of TCM-1 aimed the spacecraft toward Mars, removing a bias intended to protect Mars from contamination by the launch vehicle's upper stage. In early February, a new version of flight software was uplinked and tested on both computers. The primary payload checkout occurred March 12-22, revealing all instruments and the engineering cameras to be responsive and healthy after launch. This was followed by a successful TCM-2 on March 26, aiming the spacecraft at the desired atmospheric entry point. In mid-April, the science and engineering cameras were checked from the backup (B-side) computer, when operations were swapped to that computer for engineering activities.

In late May, the flight software load to execute entry, descent, landing (EDL), and early surface operations was uplinked and tested on both computers. In mid-June, a version of the flight software optimized for surface operations was uplinked for use after landing. The team also conducted secondary payload checkouts of REMS, the science and engineering cameras, and SAM on the A-side computer in order to compare with the first checkout.

Monte Carlo simulations of EDL performed during cruise narrowed the estimated landing error ellipse to 21 by 7 km, down from 25 by 20 km (both 3σ). The MSL Project determined that the center of the ellipse could be shifted 6.5 km southward and 1.3 km westward in order to reduce potential drive distances to Mount Sharp. The small TCM-3 maneuver (June 26) included this shift.

1.2 Preparation for Arrival at Mars

By mid-July of 2012, spacecraft operations became focused on the final approach to Mars and preparation for EDL. During this time, a working group of EDL engineers and science team members conducted a real-time monitoring campaign of Mars' atmosphere. The group assessed global imaging of atmospheric dust and water ice from the Mars Color Imager (MARCI) and atmospheric temperature retrievals near the landing site from the Mars Climate Sounder (MCS), both onboard the Mars Reconnaissance Orbiter (MRO). The purpose of these assessments, held occasionally starting eight weeks before entry and then daily in the week prior to entry, was to determine if the present state of the atmosphere differed significantly from that estimated previously (Vasavada et al. 2012) and used to design the EDL system and set flight parameters.

Of particular concern were regional dust hazes that might alter the density profile encountered by the spacecraft by 5% or more, or local-to-regional dust storms that could generate anomalous densities and winds. Study of previous Mars years near L_s=150.7° revealed low-dust conditions in Mars Years (MY) 28 and 30 but increased levels of dust in MY29. Should such phenomena be detected, be expected to occur over the landing area at the time of entry based on translation speeds, and be judged likely to persist through the

time of entry, performance simulations could be modified and flight parameters updated accordingly.

Although dust activity in Hellas picked up in the weeks prior to entry and intermittently injected dust on a trajectory toward Gale crater, final pre-entry MARCI imaging showed a cold and low-dust atmosphere over the landing site. The presence of water ice clouds further indicated the absence of significant dust. Atmospheric temperature retrievals from MRO-MCS one day prior to entry were consistent with cold and low-dust conditions. In the end, no changes were made to the EDL performance simulations or flight parameters (Chen et al. 2014a; Cianciolo et al. 2013).

Throughout cruise and approach, the interplanetary navigation proved highly accurate (Martin-Mur et al. 2014). A small, corrective TCM-4 was executed on July 28. Planned windows for TCMs 5, 5x (backup), and 6 (contingency) were not utilized given the small errors determined from ongoing navigation solutions. The spacecraft reached its atmospheric entry point with an entry flight path angle just 0.013° shallower than the -15.5° target. The navigation team updated the onboard entry state for the final time six days before entry, initializing the descent guidance system with the best estimate of the expected position and velocity at the entry point. Navigation errors continued to converge favorably as the spacecraft neared entry. Additional opportunities to update the spacecraft were not utilized, including the final chance at 8:20 P.M. Pacific Daylight Time on August 5, two hours before landing. From that point on, Curiosity was on its own.

1.3 Entry, Descent, and Landing

Nearly all critical elements of the EDL system performed as designed and simulated (Way et al. 2013). Entry heating and aerodynamic deceleration were well within design limits and closely matched predictions. Entry guidance and control delivered the vehicle to the desired parachute deploy conditions approximately 12 km above the ground. The supersonic parachute deployed nominally at Mach 1.75 and provided the predicted drag to reach the targeted heatshield separation and backshell separation conditions. The Mars Descent Imager (MARDI) was commanded to begin acquiring images about six seconds before separation of the heat shield. Powered flight started approximately 1.6 km above the ground, as expected, and completed with ample fuel margin.

Two notable anomalies occurred during EDL. First, the X-band radio shut itself down due to an internal fault, resulting in a loss of direct-to-Earth communications a few seconds earlier than expected. Spacecraft data continued to flow uninterrupted to Earth via the ultra-high-frequency (UHF) radio and the relay orbiters overhead. Second, the local gravitational acceleration in Gale crater was lower than the onboard estimate by 0.0044 m s^{-2} due to the use of a coarse gravity field representation that did not adequately capture local topography. This resulted in a softer than expected vertical touchdown velocity and a slightly higher than expected horizontal touchdown velocity. The touchdown conditions were well within the rover's capabilities.

Post-landing analyses showed that the final entry state (uploaded six days prior to entry) had a position error of ~200 m and a velocity error of 0.11 m s^{-1} (against its velocity of 5845 m s^{-1}) allowing the spacecraft to accurately guide its flight toward the landing target. Post-landing reconstruction of the as-flown atmosphere based on onboard inertial measurements, dynamic pressure sensors on the MSL heat shield, orbiter data sets, and atmospheric models determined that the atmospheric structure and dynamics compared favorably with pre-flight estimates for low-dust conditions (Chen et al. 2014b; Cianciolo et al. 2013; Karlgaard et al. 2014).

Curiosity safely touched down at Bradbury Landing (Fig. 1) on August 5, 2012 (Pasadena, California time). Additional details, including the precise time and location of landing and descriptions of post-touchdown events, are included in Vasavada et al. (2014).

2. Science Operations on Gale Crater's Plains

2.1 Bradbury Landing, Yellowknife Bay, and Dingo Gap

A detailed narrative of the mission from landing through Sol 500 that includes the commissioning phase, activities at Yellowknife Bay, and the first leg of the traverse to Mount Sharp (Fig. 1) can be found in Sect. 2 of Vasavada et al. (2014). That paper also describes the mapping quadrangles and naming conventions used by the project.

As of Sol 500, Curiosity was progressing along the Rapid Transit Route, a path charted to take the rover across the plains to the start of potential traverse routes ascending Mount Sharp (Vasavada et al. 2014). Drive progress toward the Kimberley waypoint (Fig. 2) was the top strategic priority on the Rapid Transit Route, so the science operations team attempted to plan a drive in every planning cycle, along with a focused set of observations designed around it. When it was not possible to plan a drive (i.e., because downlink from the previous drive was not yet received), the team acquired remote and contact science data on targets of opportunity, typically clasts and soil targets around the rover. The team's strategy during this phase of the mission was referred to as "Mount Sharp or Bust."

In the month prior to Sol 500, the operations team noted an alarming increase in the rate of dents, punctures, and tears on the rover's wheels and began an investigation (Vasavada et al. 2014). To minimize further wheel damage during this investigation, drive distances were limited to the extent of the stereo navigation camera (Navcam) coverage acquired after the previous drive (~20-30 m), allowing rover planners to carefully choose routes and avoid wheel hazards.

The team began 2014 by planning Sol 502 on January 2. The rover crossed into the Kimberley mapping quadrangle on Sol 513, named after the region in northwest Australia known for studies of Precambrian geology. On Sols 515 and 519, the team tested a new style of operations called "Drive-Only Sols." These sols would be planned using a 6-hr timeline for the tactical planning team (down from 10 hours) enabled by a focus on driving and environmental monitoring, and no Science Theme Groups. The shorter timeline allowed a planning shift to be scheduled on days when the downlink from Mars arrived

later in the morning, or commands needed to be sent earlier in the afternoon, than would be acceptable if using a 10-hr timeline. It was used several days each month over approximately two months in order to make progress toward the Kimberley waypoint.

On Sol 523, the team set Curiosity's near-term drive goal to Dingo Gap (Fig. 2), the entrance to a narrow valley that marked the beginning of a newly adjusted traverse route to the Kimberley region. The new route was chosen in order to favor sandy areas over rocky areas in an effort to minimize wheel wear. Orbiter images showed a large ridge of sand spanning Dingo Gap, so the team sent the rover there to assess the traversability of both the ridge and the valley beyond it (Arvidson et al. 2017). Plans for Sols 530-532 included contact science measurements on the ridge, which was intentionally scuffed by a wheel. The rover planners noted that the ridge's slope was less steep than anticipated and chose a route toward the left side of it. The Sol 533 drive was a "toe dip," designed to test the performance of the mobility system on the ridge without committing to crossing it. The rover drove to the top of the crest, with its left front wheel ending just over the crest. While images showed minimal sinkage into the sand, the rover slipped and turned, both slightly to the right. On Sol 534, a new "crabbing" drive procedure was designed to counter the slip and maintain the heading. On Sol 535, the rover crossed the ridge and proceeded down into Moonlight Valley. The team used the Mast Camera (Mastcam) to image stratigraphy exposed on the valley's walls. With much of the rover's subsequent traverses re-routed through topographic lows, where the concentration of fines provides a safer substrate for the wheels, such imaging became standard practice.

2.2 The Kimberley Campaign

After crossing Dingo Gap, Curiosity drove expeditiously along the strategically planned traverse route, including back-to-back 100-m drives on Sols 547 and 548. Some drives were done backward to distribute wear on the wheels. However, doing so required turning in place near the end of the drive to acquire images of the planned parking spot without the rear of the rover blocking the terrain. Such images are needed to assess the risk of wheel slippage when using the arm. Turning in place also adds wear to the wheels, so this strategy was eventually discontinued. On Sol 549 the rover was positioned in front of Bungle Bungle, a prominent outcrop of conglomerate investigated on Sol 550.

The Kimberley waypoint (Fig. 2) was determined from orbit-based mapping to be the highest-value science target between Yellowknife Bay and Mount Sharp—and the only one designated for drilling—because of the presence of an exposed, layered sedimentary sequence with clear stratigraphic relationships. Of particular interest was a rock unit with a striated appearance that was exposed in multiple places over a wide area on the plains (Stack et al. 2016). As the rover neared the waypoint, team discussions considered whether to continue to plan for a campaign at the Kimberley or to access another site, Kylie, that was closer to the strategic route. Stratigraphic relationships also could be studied at Kylie, but over less vertical relief than at the Kimberley. The team stuck with the Kimberley as the target but also prioritized imaging and other observations as the rover passed Kylie. Williams et al. (2018) describe key observations between Dingo Gap and the Kimberley. Sols 555-557 were dedicated to a Sample Analysis at Mars (SAM) combustion experiment.

After several weeks focused on driving, on Sol 571 the team began long-range reconnaissance imaging of the Kimberley waypoint. Sols 576 to 581 were spent cleaning and inspecting the sampling system, and bumping forward to access a dust-free exposure of the striated unit at the northern end of the Kimberley. The Square Top target was examined on Sols 583 to 585. In addition to Alpha Particle X-Ray Spectrometer (APXS) measurements and nested Mars Hand Lens Imager (MAHLI) imaging, the team acquired three 8-frame, one 12-frame, and one 20-frame spatial mosaics with MAHLI. Some of these were "dog's eye" mosaics, with MAHLI looking obliquely from a position low to the ground. Curiosity then proceeded south along the eastern margin of the Kimberley complex. Observations starting on Sol 589 offered close-up and along-strike views of the striated unit. On Sol 595, four side-looking stereo Mastcam mosaics were acquired during the drive to record the three-dimensional geometry of this unit.

Sols 603 to 609 brought the rover to the Windjana drill target, chosen in a fine-grained unit (Dillinger member of the Kimberley formation) hypothesized to be recently exposed by wind-driven scarp retreat of the overlying unit (Mount Remarkable member). After using vibration to clear surface particulates from the APXS on Sol 611, the rover began sampling activities on Sol 612. Sols 616 to 620 were spent addressing a MAHLI anomaly. A sample was drilled from Windjana on Sol 621. After analyses of the sample with the Chemistry and Mineralogy (CheMin) and SAM instruments, and measurements of the drill hole, tailings, and nearby features, the team drove the rover away from the Kimberley on Sol 630 with sample material cached in the sample processing system. Rice et al. (2017) presents an overview of the Kimberley campaign and key findings.

2.3 Completing the Traverse to Mount Sharp

Drive progress was excellent after leaving the Kimberley, helped by relatively benign terrain and the use of auto-navigation (now back in use after the wheel anomaly was better understood). The project also identified the dates over the coming months when using a shorter 7-hr planning timeline and reduced activities would enable more drives. This strategy was similar to Drive-Only Sols (Sect. 2.1), but re-designed as “Rapid-Traverse Sols” with the addition of more planned remote science observations. This strategy was used until the rover reached Mount Sharp. On Sol 636, the rover crossed into the Hanover mapping quad, named after the Hanover Shale in the northeastern United States. A triple-portion of the cached Windjana sample was delivered to SAM on Sol 653 for a noble gas extraction experiment. The drive on Sol 665 was the longest of the mission to date at 142.5 m, enabled by smooth terrain, a long view in the stereo Navcam images, and the use of autonav to drive beyond the stereo mesh.

After completing its first Mars year on Sol 669, the rover crossed into the Shoshone mapping quad on Sol 670, themed after geological features around the Mojave Desert in the southwestern United States. The drive on Sol 672 took Curiosity right to the edge of its landing ellipse (Fig. 1 of Vasavada et al. 2014), only to be stopped by a fault when high wheel slip occurred on sand ripples. On Sol 673, the team examined sand that had been disturbed by the wheels and on Sol 674, the rover exited its landing ellipse. A test of the

rover's mobility system on Sol 683 had the rover drive over a sand ripple while engineering telemetry and images were collected at high rates. The goal was to understand the performance and risk on sand, since the strategic route chosen to minimize wheel damage was often routed through valleys where sand patches and ripples were present.

Sol 688 began a multi-sol traverse over rugged terrain on Zabriske plateau (Fig. 3), resulting in shorter drives in light of increased risk of wheel damage. On Sol 694, another triple-portion of the cached Windjana sample was delivered to SAM for a later experiment. The team chose not to command science activities on Sols 697-699 while a problem with the rover's backup computer was assessed. On Sols 704 and 705, the remaining Windjana sample material in the sampling system was dumped and analyzed, and the hardware was cleaned.

The rover descended on Sol 706 into Hidden Valley, a U-shaped valley with rippled sand covering the entire floor. It had been hoped that the rover could cross ripple fields safely or skirt around them, even if it meant driving off camber on the lower part of the valley wall. Finding no traversable path around the ripples, on Sol 709 Curiosity attempted to drive across them to the other side of the valley, where the lower wall appeared traversable. The drive faulted due to very high slip rates, however, raising concerns about the rover's ability to traverse rippled deep sand (Arvidson et al. 2017). In addition to becoming stuck, there also was a concern that if the rover made progress along downward-sloping sand but then lost traction, it might not be able to retrace its path uphill. With no obvious side exits in the remainder of Hidden Valley, the team opted to reverse out of Hidden Valley and drive westward toward another entrance to the system of valleys that would eventually take the rover southward.

By Sol 714, the rover was on the plateau above Hidden Valley and the team was scouting for an alternate path. Meanwhile, the science team was considering whether to drill a sample of the hard sandstones typical of the plateaus or the recessive, fine-grained bedrock seen in the walls of Hidden Valley. The team chose to drill at Bonanza King, a small outcrop of fractured, recessive bedrock located on the entrance ramp into Hidden Valley. Although the target remained stable when pushed by the arm on Sol 722, it broke apart and shifted during the drilling of a test hole on Sol 724. After some deliberation, project management decided that it was not safe to proceed and that similar nearby targets also were likely to fail during drilling.

Curiosity traversed across the plateau and arrived at the entrance into Amargosa Valley on Sol 742. This valley allowed for a few long drives, interspersed with some shorter drives to occasionally ascend ridges to permit longer views for route planning. With the descent into Amargosa Valley, the rover crossed out of terrain associated with the plains surrounding Mount Sharp, and into terrain associated with Mount Sharp itself. The drive on Sol 753 took the rover to the base of Pahrump hills (Fig. 3), a set of hills with outcrops of light-toned, recessive bedrock first identified in orbiter data. This isolated exposure was the first one reached by the rover that could be tied through orbiter imagery to the Murray formation, the basal layer of Mount Sharp.

3. Science Operations at Mount Sharp

3.1 Pahrump Hills Campaign

Upon arrival at the base of Mount Sharp, the strategic route was adjusted to begin climbing the mountain in southwest direction, rather than continuing west another two kilometers to begin the climb within the Murray buttes. The science team decided to slow the drive pace and study the Pahrump hills section (Stack et al. 2019) in detail. The section would provide a baseline understanding of the Murray formation that the rover would be driving through for at least 150 vertical meters, according to orbit-based maps. For example, what is its stratigraphic relationship to the surrounding plains, and what was its depositional environment and history of water-rock interaction? Further, after fourteen months focused on driving, project and NASA management agreed that investigating these first outcrops of Mount Sharp was appropriate, even considering the cost to total drive progress over Curiosity's lifetime.

As the rover approached Pahrump hills, long-range imaging revealed that the lighter-toned rock that gives it a distinctive appearance in orbiter images was exposed in a section about 13 m thick. Occasional beds of more resistant rock also were present. Geologists on the team suggested that an initial walkabout of the section would reveal its basic characteristics and help the team select areas for additional, resource-intensive study. In the interest of managing time, this first pass would be a chemo-stratigraphic survey involving only ChemCam and mast-based imaging. A second pass would add contact science observations at a small number of sites. There was some debate about whether the contact science sites should be placed at regular intervals of stratigraphic height to provide unbiased profiles of chemical and physical properties. However, the team opted to choose sites that contained type examples of particular characteristics or that exposed key relationships. Finally, a sample would be drilled from the base of the section before starting the walkabout. Further drilling decisions would be based on results from the first and second passes through the section.

Sols 755 and 756 were used to analyze and test-drill the bedrock target Confidence Hills. The drilling sequence was interrupted briefly to examine a new type of erosion-resistant feature on Sol 758. Drilling took place on Sol 759. Curiosity’s Prime Mission ended on Sol 766. By Sol 780, the sample was analyzed by CheMin and SAM, and the rover proceeded toward Book Cliffs, the first stop on the walkabout. Along the way, the remaining Confidence Hills sample was dumped and the sampling hardware was cleaned. On the nights of Sols 782-784, Curiosity observed Comet C/2013 A1 (Siding Spring) with Mastcam and ChemCam. The comet was tracked successfully and detected by the Mastcam.

The walkabout included stops at Book Cliffs, Alexander Hills, Chinle, and Whale Rock. The rover traversed back down to the base of the section on Sol 797. Along the ascent and descent, MARDI captured a continuous sidewalk mosaic of nadir-pointed images with sufficient overlap to enable stereo. Before proceeding with the second pass, the team decided to perform a wheel scuff and analysis on a sand ripple, a lien remaining from the issues in Hidden Valley. This also gave the team time to analyze the data sets from the

walkabout and to select the sites for further study. Sol 799 had the rover use its right front wheel to scuff a ripple, while Sols 801 to 803 included multispectral imaging and compositional measurements of the disturbed sand and the wall of the trench left by the wheel. Downlink from these sols revealed an anomaly in the laser used to focus ChemCam (Sect. 8.2 of main paper).

The second pass of Pahrump hills began on Sol 803 with a drive back to the base of the section and remote and contact science measurements near Confidence Hills on Sol 805. The rover then drove to Pink Cliffs (Sol 807), Book Cliffs (Sol 812), Alexander Hills (Sol 817), Chinle (Sol 826), and Whale Rock (Sol 837).

After a two-week holiday break, the science and operations teams completed the observations at Whale Rock and brought the rover on Sol 862 near Mojave, the first target selected for drilling because of the millimeter-scale crystal forms observed on the rock. The drive to Mojave included a leg that re-climbed toward Whale Rock from a different azimuth in order to assess the three-dimensional structure of bedding within the outcrop. Drill activities began on Sol 867 and included a self-portrait acquired with MAHLI on Sol 868. Sols 873 to 879 were used to uplink a new version of the rover's software. After test drilling broke the surface of Mojave, the team re-targeted at Mojave2, where a sample was successfully collected on Sol 882.

By Sol 896, the sample was analyzed by CheMin and SAM, and two additional triple-portions were delivered to SAM for future analyses. The remaining material was dumped and analyzed on the ground, the sampling hardware was cleaned, and the rover drove toward Whale Rock to scout for a drillable bedrock target there. The science team desired to drill at Santa Ana based on data from the second pass, but the rover tilt would have significantly complicated the drilling process. Instead, the team searched for alternate targets at a similar stratigraphic level and choose one that was about 5 m from Santa Ana. The outcrop exposure was small and the rock was heavily fractured, but chemical measurements showed a composition similar to that of Santa Ana. Telegraph Peak was reached on Sol 903 and a sample was drilled on Sol 908.

The team commanded a drive out of Pahrump hills on Sol 912, with plans to execute the CheMin and SAM analyses of Telegraph Peak along the traverse. However, on Sol 911 (executing on Mars while Sol 912 was being planned), an electrical anomaly occurred when transferring material from the drill to the sample processing system (Sect. 8.1 of main paper). The rover's fault protection system halted further activities. The configuration of the arm and sampling system was kept unchanged through Sol 921 while the team gathered diagnostic data. The interrupted sampling activities resumed on Sol 922, including a delivery to CheMin. On Sol 923, the 6.5-month Pahrump hills campaign concluded as the rover drove toward Garden City with Telegraph Peak sample material cached in the sample processing system.

3.2 Artist's Drive and Logan Pass

Three science objectives guided the planning of the traverse through Artist's Drive (Fig. 3), the valley above Pahrump hills: i) find an accessible exposure of the bright and dark mineral veins observed below Salsberry peak; ii) approach and analyze a boulder that may have fallen from that peak; and iii) image the stratigraphy exposed in the surrounding walls. The Garden City vein complex satisfied the first objective for remote and contact science, but no drillable targets were found. The rover arrived at Garden City on Sol 926 and delivered Telegraph Peak sample material to SAM for analysis before investigating the veins.

On Sol 940, the rover traversed to Kanosh, a boulder that likely derived from Salsberry peak. Measurements on Kanosh accomplished the second objective on Sol 942. After analysis and discussion of the initial results from Garden City, the team chose to return there for additional contact science on Sols 946-948. On Sol 949, Curiosity addressed the third objective by returning to driving while imaging stratigraphy exposed on the valley walls and surrounding hills. Imaging locations were carefully chosen from orbiter images to provide views through low points in the near-field topography.

Curiosity descended from Artist's Drive into a set of valleys that led toward Logan pass (Fig. 4), located above a steep ravine which the rover would need to ascend to continue its journey south. Sol 954 was used to deliver cached Telegraph Peak sample material to SAM for future analysis, after which the remainder was dumped and analyzed. Mastcam images showed that the dumped material was intermingled with pebbles and the rough substrate, so an additional APXS measurement of only the background materials was acquired.

The rover crossed into the Arlee mapping quad on Sol 956, with targets named after geologically significant sites around western Montana in the United States. MRO-HiRISE maps showed that the bright, recessive mudstones studied at Pahrump hills continued to crop out along the lower walls of valleys that separated mesas capped by erosion-resistant rock. Logan pass sits between Apikuni mountain to the north and Mount Stimson to the east. MRO-HiRISE images revealed a large exposure of mudstone on the north face of Mount Stimson in contact with the darker, bedded rocks of the unconformably overlying Stimson formation (Banham et al. 2018). This site was chosen as a waypoint in order to provide a first look at the Stimson formation. The team was particularly interested in understanding the depositional environment recorded by the Stimson, its composition, and the nature of its contact with the Murray formation.

As the rover approached Logan pass, science team members noted a topographic ridge to the west with an unusual bedding geometry. Mudstone was present along most of the ridge, but beds of darker, more resistant rock were present at one location. The lateral extent of the darker beds increased with stratigraphic height, forming an inverted triangle. The team hypothesized that the mudstone was incised by a channel and filled with coarser material. The importance of such a structure for understanding ancient environmental conditions resulted in a vigorous team discussion and decision to plan a ~ 100-m out-and-back excursion (called "Logan's Run") to the base of Mount Shields. The rover drove to the area on Sols 967 and 971, performed contact science on the lower lens of putative fill material on Sols 974 and 975, and returned to the strategic route on Sol 976.

The rover crossed through a saddle between Jocko butte and Apikuni mountain on Sol 981, entering the valley at the base of Logan pass. Navcam images showed that a rippled sand patch first seen in MRO-HiRISE images was likely to pose a significant mobility challenge. The team decided to skirt around the sand toward the mudstones on Mount Stimson. However, the rover faulted to due excessive slip on Sol 983 when the left wheels encountered deep sand. An attempt to drive higher on the sloping valley wall on Sol 984 was terminated by the rover when it began slipping laterally downhill. These drives were the most challenging attempted in the mission at the time, due to the presence of sand, loose gravel, high pitch (maximum of 16° backward), and high roll (maximum of 16° to the left, for a maximum tilt of 22°).

Given the scientific importance of understanding the relationship between the Murray and Stimson formations, the team made a considerable effort to find either an alternate path for accessing Mount Stimson or other exposures of the contact on MRO-HiRISE and rover images. As the rover backtracked to the saddle near Jocko butte, mobility engineers and science team members assessed the traversability, wheel risk, and length of particular routes. The full science team was asked to provide feedback on the scientific suitability of the routes and on how much mission time was worth spending on reaching the contact.

3.3 Marias Pass

The science team and project management ultimately decided to access a smaller exposure of the contact at Marias pass (Fig. 4), located at the top of a ravine on the other side of Apikuni mountain from Logan pass. It hinged on the successful climb of a 6-m hill. The science team decided that if the climb was unsuccessful, the rover would resume climbing Mount Sharp via an alternate route and give up on studying the contact in this area. Sol 990 brought the rover to the base of Mount Shields below Marias pass. After choosing to ascend a spur rather than the ravine, the team successfully commanded the ascent on Sol 991. Moderate slip occurred, but a lack of sand and the presence of bedrock helped the rover find traction even with a maximum slope of 21°. From the top, the science team could see that the outcrops of interest—only visible in MRO-HiRISE images up to this point—were largely free of debris and accessible to the rover. On Sol 995, Curiosity drove around sand toward the far end of the exposure. Contact science was carried out on Sols 998 and 999. As of Sol 1000, Curiosity had driven 10.8 km from Bradbury Landing, the farthest of any Mars rover at a similar point in its mission.

Throughout May 2015, the operations team prepared for the second solar conjunction. A set of sequences to execute Rover Environmental Monitoring Station (REMS), Radiation Assessment Detector (RAD), and Dynamic Albedo of Neutrons (DAN) observations was uplinked in advance for execution starting on June 3 (Sol 1005). Sols 1000-1002 were restricted in scope as rover mechanisms and instruments were progressively placed (and verified) in safe configurations. Daily transfers of data to MRO and Odyssey continued during conjunction, with the orbiters storing the data until communication with Earth resumed after three weeks.

Curiosity resumed operations on Sol 1027. A bump on Sol 1030 allowed close-up inspection of the contact at Missoula using a dog's eye MAHLI mosaic. The team decided to drive back toward the Sol 991 area on Sol 1035 to follow up on elevated silica and hydrogen measurements from ChemCam and DAN, respectively, that were discussed by the science team over conjunction.

The rover was briefly directed to head back toward the Missoula area for a drill attempt in the Stimson formation, but the team decided instead to drill the atypically high-silica mudstone of the Murray formation discovered near the Sol 991 area. Rocks in that area such as Lamoose were too small for drilling, so on Sols 1049-1056 the rover was commanded to approach an area to the southeast with exposed bedrock that was thought to be laterally equivalent. After ChemCam confirmed the elevated silica, the team decided to drill at Buckskin. The drill site was a compromise because the bedrock had only intermediate silica abundance, but it was accessible to the brush and drill from a stable parking spot. The drill campaign began on Sol 1057 and completed with a successful sample acquisition on Sol 1060 followed by delivery to CheMin on Sol 1061. Sample material was cached in the sample processing system for later delivery to SAM.

On Sol 1066, planned on the 3-year anniversary of Curiosity's landing, the rover turned back to Missoula to address additional questions about the Murray-Stimson contact. Curiosity continued on Sol 1070 through Marias pass and onto the Stimson formation at Emerson plateau (Fig. 5), resuming its strategic traverse with a next major waypoint set at the Bagnold dune field. Stops were made at a spectacular exposure of the Stimson at Williams on Sols 1087-1094 and an exposure of the underlying Murray (putative) at Bridger Basin on Sols 1105-1109. Buckskin sample material was delivered to SAM on Sols 1075 and 1089, with the remainder dumped on the ground for observation.

After noting discoloration and compositional differences of the Stimson bedrock in the local area, especially along fractures, the science team decided to search for drillable targets both inside and away from a fracture-associated halo. Big Sky, a typical Stimson target, was successfully drilled on Sol 1119, followed by deliveries to CheMin and SAM. The rover approached the halo target Greenhorn on Sol 1127, drilled a test hole at nearby Pilgrim, and successfully acquired sample material from Greenhorn on Sol 1137. Material was delivered to CheMin (and SAM after driving away). The Greenhorn drill campaign was interleaved with the final activities of the Big Sky campaign, as well as two SAM measurements of atmospheric methane.

After re-orienting to place DAN over Greenhorn for neutron spectroscopy, Curiosity headed on Sol 1144 to a position where it could acquire a context mosaic of the floor of the Bridger basin. Although this area was the subject of vigorous discussion due to very high silica abundances, unique color and texture, and potential association with the Murray-Stimson contact, it ultimately was not selected for drilling. The rover then proceeded along the strategic route in a series of longer drives. On Sol 1157, the team tested new software that allowed bedrock and clasts to be autonomously detected in Navcam images and targeted with ChemCam. Curiosity entered the Windhoek mapping quadrangle on Sol 1158 en route to the Bagnold dune field. The quad was named in recognition of geologically significant

sites in Namibia, particularly around the Namib and Kalahari deserts. Target names were compiled by staff at the Gobabeb Research and Training Center in Namibia. On Sol 1160, the team studied the dark, circular Brandberg feature.

3.4 Bagnold Dunes Campaign

The planning and implementation of the first of two campaigns investigating the Bagnold dune field is summarized in Bridges and Ehlmann (2018). Drives on Sols 1162, 1167, and 1168 headed toward High and Namib dunes (Fig. 5) while orienting the rover to survey the wind field over all azimuths. The goal was to observe the wind field in the vicinity of the dunes, but apart from their influence. The team collected reconnaissance images and spectra of the dunes during the approach. On Sol 1174, the rover reached patches of rippled sand near High dune. Because the upcoming strategic route also included surfaces partially or fully covered by rippled sand, on Sols 1181-83 the mobility engineers characterized the rover's ability to drive on a sand patch by traversing it while collecting high-rate telemetry.

After High dune, on Sol 1185 Curiosity began driving to the south of Namib dune, where the team monitored its wind environment and slip face. An arm fault occurred on Sol 1198 while dumping the Greenhorn sample onto the ground for observation. The material was analyzed on Sols 1202-04 after recovery. A set of pre-made plans kept the rover busy while the team took a holiday break on Sols 1206-1213. On Sol 1215, the rover drove to the west side of Namib dune so that the rover could scuff the secondary slip face with its right front wheel and study the scuffed and undisturbed areas.

With the rover at the scuffed sand target, Gobabeb, a scoop-based sampling campaign began on Sol 1221. A complex series of processing steps on two samples scooped on Sols 1224 and 1228 provided the < 150-μm fraction to SAM and CheMin, the fraction between 150-μm and 1 mm to SAM, and created piles of these fractions on the ground, as well as the > 150-μm fraction, for observation by remote and contact science instruments. A third scoop on Sol 1231 was intended to double the amount of the middle size fraction delivered to SAM, but an anomaly in the sample processing system prevented delivery.

3.5 Naukluft Plateau, Return to the Murray, Murray Buttes

The rover drove onward on Sol 1248 toward the Naukluft plateau (Fig. 5), an exposure of the Stimson formation that the strategic route required ascending and crossing before returning to the Murray formation. After a few sols studying the Murray-Stimson contact, the rover ascended onto the Naukluft plateau on Sol 1281. The drive across the plateau was slow because of the roughness of the eroding sandstone. On Sol 1316, as the rover was nearing the western edge of the plateau, the team decided to backtrack to an area where ChemCam had once again detected silica enrichments in fracture-associated haloes. A drilled sample was collected on Sol 1320 near a fracture at Lubango. The rover drove a short distance away from the fracture on Sol 1329 and collected a drilled sample at

Okoruso on Sol 1332. Sol 1339 marked the completion of Curiosity's second Mars year on the surface.

On Sol 1353, the rover descended back onto the Murray formation. The successful traverse across the Naukluft plateau brought the rover to a milestone on its strategic route up Mount Sharp. From Yellowknife Bay until this point, the rover followed a southwest heading in order to align itself directly north of a gap in the east-west trending Bagnold dune field. This gap was identified as one of the only places to cross the dunes without encountering deep sand. Now the route turned south and the rover more directly ascended the mountain. Concerns over too rapidly ascending through the uninterrupted stretch of Murray formation ahead and missing informative changes in mineralogy led the science team to adopt a strategy of "survey drilling" every ~25 meters of elevation gain. The strategy was used for the next four drill sites until precluded by a major anomaly with the drill. Efficiency was improved by streamlining the drilling procedures and leaving each drill site with cached sample material before delivering to SAM. A drilled sample was collected at Oudam (Fig. 6) on Sol 1361.

On Sol 1369, the rover continued south through Hartmann's Valley toward the Murray buttes (Fig. 6), a set of spectacular sandstone-capped towers. Following similar studies at the Emerson and Naukluft plateaus, the team used stereo imaging of the capping units acquired between Sols 1383 and 1455 to characterize the types of dunes and sediment transport directions recorded in the rock. Between Sols 1389 and 1397, science activities were put on hold while the operations team diagnosed an anomaly with the rover's onboard data product catalog that caused an unexpected flight software reset. On Sol 1399, Curiosity approached the Bimbe blocky deposit, one of several such features seen in MRO-HiRISE images, and investigated it at multiple locations through Sol 1410.

On Sol 1418, a drilling campaign began at Marimba. Sample material was collected on the second drill attempt on Sol 1422, after drilling made insufficient progress on the first attempt. The rover moved onward on Sol 1427, heading expeditiously through the Murray buttes to the next drill site. A sample was collected on Sol 1464 at Quela, adjacent to one of the Murray buttes. The rover drove onward on Sol 1468 after successfully finishing the campaign. Curiosity began its second extended mission (EM2) in route to the fourth survey drill site. Sebina was successfully drilled on Sol 1495. A new mapping quadrangle, Bar Harbor, was reached on Sol 1500, themed after geologically significant areas around the state of Maine in the northeastern United States.

As the rover neared the gap where it would cross through the dune field, sand coverage of the surface increased, sometimes limiting access to bedrock. On Sol 1521, the team began investigating an atypically dark outcrop called Sutton Island (Fig. 7) and its surroundings. The fifth survey drill site at Precipice was reached on Sol 1526, but drilling on Sol 1536 was precluded by a fault in the drill's feed mechanism. While the engineering team assessed the anomaly, the science team noted that the combination of seasonal winds and abundant sand supply resulted in considerable windblown sand, and discussed how to minimize risk to the rover's instruments. On Sol 1543 and after extensive development and laboratory testing, SAM conducted its first "opportunistic derivatization" experiment that exposed an

onboard drilled sample to the residual vapors of a wet chemistry reagent that had leaked from a set of sealed sample cups.

3.6 Second Crossing of the Bagnold Dune Field

Driving resumed on Sol 1553 when the engineering team completed its initial diagnostics of the drill feed and permitted use of the arm and mobility system. Drilling remained indefinitely precluded, however. With 2016 coming to a close, the team uplinked a pre-planned set of activities for Sols 1558-1565, focusing on change detection and meteorology. The final plan of the year investigated a bedrock slab with intricate ridge and fracture patterns called Old Soaker and its surroundings, to be executed in early 2017 on Sols 1566-68. Given the prolonged drill feed issue, the science team decided to suspend the survey drilling goal and continue ascending. The team imaged the stratigraphy exposed on Ireson hill from different angles as the rover traversed south and then southwest toward the first waypoint of the second Bagnold dunes campaign (Lapotre and Rampe, 2018).

On Sol 1602, the team began investigating Nathan Bridges dune (named after an MSL team member who was an expert in aeolian processes) at Mapleton (Fig. 7) after intentionally scuffing it with the rover's wheel. On Sol 1604, the team steered the rover back to Ireson hill for closer observation, including contact science on blocks that tumbled down the hill. Sol 1617 had the rover at a second scuffed workspace called Sandy Point Beach on Nathan Bridges dune. The rover's departure was delayed until Sol 1628 to diagnose an issue with the MAHLI instrument, allowing additional change detection observations. On Sol 1637, the team began investigating a third scuffed workspace at Southern Cove. The rover drove off on Sol 1639 toward the final campaign waypoint at Ogunquit Beach at the Mount Desert Island ripple field. The drive on Sol 1645 featured the first use of traction control software (Sect. 8.1 of main paper).

The campaign called for scoop-based sampling at Ogunquit Beach, so after arriving and scuffing the workspace on Sol 1649, the rover acquired the first scoop, sieved it to < 150 μm, and delivered it to SAM on Sol 1651 for analysis. During delivery to CheMin on Sol 1652, diagnostics of the drill feed showed further degradation, potentially related to the use of the sample processing system. Out of caution, delivery to CheMin was deferred (and finally achieved on Sol 1831!), additional scooping at Ogunquit Beach was cancelled, and the rover continued along the strategic route on Sol 1659. On Sol 1686, the rover scuffed and parked at a megaripple. Its investigation on Sols 1687-89 was the last of the Bagnold dunes campaign.

The priority turned to making progress southeast while continuing to document blocky outcrops of the Murray formation among the sand. As the path uphill steepened, the science team decided to conduct contact science measurements at least every 5 m of elevation gain. Sols 1722-23 were dedicated to a SAM analysis of the Quela sample that had been carried within the instrument.

3.7 Vera Rubin Ridge Campaign

Vera Rubin ridge (VRR) is a geomorphic feature that is coincident with spectral absorptions in MRO Compact Reconnaissance Imaging Spectrometer for Mars (CRISM) data that are attributed to hematite. Its distinctive texture, expression, and mineralogy in orbiter data made it a key target for the mission. Fraeman et al. (2020) describe the goals and implementation of the VRR campaign. With the rover's drilling capability still unavailable, the campaign first involved imaging and collecting spectral data upon approach, followed by an extended reconnaissance of the ridge.

Upon reaching VRR, the strategic route turned eastward and paralleled the northern face of the ridge as it headed toward a location with slopes shallow enough for the rover to ascend. Five imaging stops were chosen to document the stratigraphy exposed on the northern face of the ridge and the relationship to underlying units. Sol 1726 brought Curiosity to the first imaging stop of the campaign. On Sol 1748, the rover drove to a ripple field and scuffed part of the workspace with its wheel. Sols 1749-50 were spent investigating the disturbed and undisturbed sand, particularly around a larger ripple crest. The third solar conjunction of the mission prevented communication on Sols 1759-79, so the team sent a pre-planned set of activities that focused on environmental monitoring. Curiosity began its sixth year on Mars when operations resumed on Sol 1780.

After additional imaging stops, the rover turned south on Sol 1799 (Fig. 8) and began ascending the ridge while regularly stopping for remote and contact science. The ascent also provided broad views of Gale crater's floor, including much of the path taken by the rover since landing. The rover completed the ascent by Sol 1814 and began investigating meter-scale fractures on its upper surface. Meanwhile, cached sample material from Ogunquit Beach was delivered to CheMin and SAM and analyzed by those instruments. The team steadily advanced the rover southward over the next few months. Curiosity reached the Kuruman mapping quadrangle on Sol 1828, with a theme chosen around geological sites in South Africa including the Kuruman banded iron formation. Shortly thereafter, Curiosity reached the Torridon mapping quadrangle on Sol 1896, named in recognition of areas in Scotland where key discoveries were made about the age of the Earth and the origin of life. The campaign next focused on small patches on top of VRR of the surface that appear blue-gray in MRO-HiRISE images, reaching one on Sol 1901 and subsequently adjusting the strategic route to visit additional examples.

From Sols 1854 to 1963, operations planning was restricted to three days a week (instead of an average of four) so that additional engineers could spend additional time recovering the mission's drilling capabilities. Sols 1913-1920 were planned in advance covering the end-of-year holidays. By Sol 1940, the team got its first look at the "clay-bearing unit" that had been hidden behind the ridge since landing.

Curiosity started a drill campaign at Lake Orcadie on Sol 1966 using the feed-extended drilling technique for the first time (Sect. 9.2 of main paper) in a non-percussive (rotary-only) mode. The Ogunquit Beach sample material was dumped on Sol 1969 and analyzed on the ground over subsequent sols. Unfortunately, the hole drilled on Sol 1977 was too shallow to acquire sample material. In case any heterogeneity in the rock worked in the drill's favor, the team attempted to drill nearby at Lake Orcadie 2 on Sol 1982, but again

failed to reach sampling depth. With percussive drilling expected within several weeks, the team decided against further rotary-only attempts and drove onward.

The traverse along the ridge provided terrific imaging of the crater floor and Peace Vallis to the north, as well as the Greenheugh pediment and higher units on Mount Sharp to the south. The rover turned northeast to an area with especially deep spectral absorptions attributed to hematite in MRO-CRISM spectra and made observations of that area on Sols 2004 to 2014. Curiosity completed three Mars years of surface operations on Sol 2006. The rover briefly entered the Biwabik mapping quadrangle on Sol 2004 that is named in recognition of the Iron Ranges and the Duluth Complex near Lake Superior in central North America. Over Sols 2015-2020, the team investigated Bressay, a collection of diverse and seemingly out-of-place rocks reminiscent of other enigmatic blocky deposits seen prior to the VRR.

The science team decided to interrupt the VRR campaign in order to drill a sample from the Blunts Point member below the ridge, since the drill was not available when the rover initially traversed through that region. This diversion also offered a rare opportunity to study the lateral equivalents of strata encountered during the ascent of the ridge to the west. On Sol 2027, the rover re-entered the Biwabik quad as it drove to a location where it could descend the north face of the VRR. Curiosity acquired stereo images of Taconite, a putative small impact crater, on Sols 2032-2035. The rover arrived at Duluth on Sol 2053 and successfully collected a sample on Sol 2057 using rotary-percussive feed-extended drilling. Because the loss of the drill feed motor prevented sample material from being transferred to the sample processing system for sieving and portioning, portions were delivered directly from the drill bit into the CheMin and SAM inlets.

Three test portions were delivered to the workspace on Sol 2059, followed by delivery to CheMin on Sol 2061. Because CheMin did not receive sufficient sample material, additional test portions were delivered to the closed SAM inlet cover (to duplicate the rover deck environment) and to the workspace. To mitigate the risk of low portion volume on CheMin's second try, three portions were delivered on Sol 2068. A lower drop-off height also was used. The subsequent successful CheMin analysis marked the full recovery of drill sample analysis capability after it was lost on Sol 1536. SAM received the Duluth sample on Sol 2071.

On Sol 2074, the team initiated a global dust storm campaign involving increased meteorological measurements in response to a large, growing dust storm observed by MRO on the other side of the planet. Curiosity completed the drill campaign at Duluth on Sol 2084 and began heading north to re-ascend the ridge. By Sol 2085, the sky dramatically peaked in darkness as dust from the now planetary-scale storm arrived over Gale crater. The rover re-entered the Torridon quad on Sol 2094.

The team resumed its study of VRR on Sol 2100 with a focus on acquiring drilled samples from the Pettegrove Point member (near the deep absorption features in MRO-CRISM spectra) and the red and gray facies of the Jura member, returning to sites previously visited when percussive drilling was unavailable. Drilling was attempted within the

Pettegrove Point member at Voyageurs on Sol 2112 and Ailsa Craig on Sol 2122, but each failed to reach sampling depth. The team began using indirect indicators of rock hardness to select targets that might be less resistant to drilling. These included the presence of scratches from the rover's brush, whether the rock appears to erode faster than mineral veins within it, and any clues from the local topography (e.g., recessive layers or outcrops). The presence of angular facets or wind-carved flutes also was considered to indicate harder rocks.

The team agreed to try a third and final time to sample the Pettegrove Point member at Stoer. The attempt on Sol 2136 was successful and sample material was delivered to CheMin and SAM. On Sol 2156, the rover began heading toward the previously visited Loch Eriboll area, where both red and gray Jura outcrops are present. On Sol 2159, a noble gas experiment was run on the Quela sample that had been carried within SAM since Sol 1484. Drilling was attempted on the gray Jura target Inverness on Sol 2170 but failed to reach sampling depth. On Sol 2172, as the rover was completing observations of the partial drill hole and tailings, it experienced an anomaly in its file system that led to a lengthy recovery. Diagnostic activities occupied plans until Sol 2204, when science operations resumed on the rover's alternate (A-side) computer after a side swap on Sol 2188. Environmental monitoring with imaging, DAN, REMS, and RAD resumed first, followed by contact science and mobility by Sol 2218.

After wrapping up activities at Inverness, the rover headed back to the Lake Orcadie area. Although the two earlier rotary drilling attempts there were unsuccessful, their telemetry suggested that the rock was potentially softer than at Inverness. Drilling was successful at Highfield on Sol 2224 and sample material was delivered to CheMin and SAM. The Highfield sample was dumped on Sol 2245 and the rover drove onward on Sol 2250 toward a red Jura target for drilling, if one could be found along path back to the strategic route. Red Jura rocks had been associated with highly fractured outcrops and rubble, and indeed, the team inspected a few potential sites but found them unsuitable for drilling. Drilling was successful at Rock Hall on Sol 2261, and sample material was delivered to CheMin and SAM.

On Sol 2265, the team sent an 11-sol plan to cover the winter holiday period. The Sol 2276-78 plan also was uplinked prior to the holidays, but executed on Mars just prior to the team's resumption of science operations in the new year. The Rock Hall sample was dumped on Sol 2288. On Sol 2296, Curiosity drove away from Rock Hall, having collected drilled samples from the three highest-priority rock types of the VRR. The mission's focus now turned toward exploration of the clay-bearing unit.

3.8 Glen Torridon Campaign

After acquiring a number of panoramas from the VRR, Curiosity descended the ridge on Sol 2302 and began exploring Glen Torridon (Fig. 9), the valley south of the ridge that is associated with spectral absorptions in MRO-CRISM maps attributed to smectite clay minerals. Like the VRR, the clay-bearing unit within Glen Torridon was held as a major target for the mission and its exploration and objectives were planned in advance by a campaign working group. The group designed a traverse that would take the rover across

three subunits defined on orbiter images: the smooth, ridged clay-bearing unit, the fractured clay-bearing unit, and the fractured intermediate unit. Campaign objectives included the investigation and sampling of each subunit.

Much of the surface of the smooth, ridged unit was covered with small pebbles and sand, creating challenges for the rover's visual odometry system that uses distinct terrain features to aid rover navigation. The rover traversed eastward along the south side of the ridge toward the lowest-elevation area in Glen Torridon, which also is associated with the strongest spectral absorptions attributed to smectites. The science team characterized a diversity of surface materials, including abundant pebbles, dark sand, sand ripples, pebble-covered ridges, and occasional larger boulders and bedrock exposures. On Sol 2320, the rover proceeded to a pebble-covered ridge, created wheel imprints, backed up and imaged the ridge and wheel-disturbed areas, and then continued onward. Sols 2322-2332 were devoted to addressing another anomaly with the rover's A-side computer's memory.

By Sol 2338, the team finished investigation of an outcrop within a local area of bedrock exposures, and headed toward the Milltimber outcrop. After another unexpected reset of the A-side computer, the project made the decision to swap back to the B-side computer, which had been reformatted and prepared for operation after its anomaly several months earlier. On Sol 2347, science operations resumed. The science team turned the rover around to inspect Muir of Ord, a tilted block with exposed layering and a cracked texture. Sol 2359 was dedicated to investigating an area of bedrock exposures, including Woodland Bay.

Sol 2365 brought the rover to Aberlady, the first drill target in Glen Torridon. It was confirmed to be in family with the low-K, high-Mg composition measured in larger blocks in the Glen Torridon region. There was considerable discussion among the team whether this compositional class, or the high-K, low-Mg class measured primarily in the widespread pebbles, would be more representative of the materials that result in the spectral absorptions detected from orbit. The team decided to drill at Aberlady and keep a lien to attempt to sample the other composition. Aberlady was successfully drilled on Sol 2370 using only rotary motion, although percussion was enabled if needed. The rock appeared to fracture during drilling, raising the question of whether powder was reliably collected. The team decided to deliver material to CheMin (with success), but SAM did not wish to risk an empty analysis.

Selection of several of the drill targets in Glen Torridon was made more challenging by the need to accommodate up to three SAM experiments (evolved gas analysis, gas chromatography/mass spectroscopy, and wet chemistry), each requiring separate sample deliveries. When using the new drilling methods, two drill holes are required to ensure that enough sample portions are available for portion tests, CheMin, and three SAM experiments. The Aberlady site had room for two holes, but with the partial failure of the first hole, it was not immediately clear whether there was room for a third. The team then debated whether to drill again near Aberlady, drive back to a previously characterized site, or further explore the low-elevation region in search of a new drill target. After sampling engineers were able to locate an alternate site near Aberlady with room for two holes, the

team decided to slightly adjust the rover's position and re-start the drill campaign at that site, Kilmarie. Kilmarie was successfully drilled on Sol 2384 and sample material was delivered to CheMin and to SAM for two experiments. The SAM team chose not to pursue a third experiment, so a second drill hole at Kilmarie was not required.

The rover used its wheel to scuff sand within a nearby ripple field Rigg on Sol 2408 and made measurements of the grain size distribution and composition on a ripple crest, trough, and a secondary ripple. On Sol 2412, the team drove the rover toward the far side of the blocky region around Aberlady and Kilmarie, with objectives of fully exploring the low-elevation area and searching for a high-K, low-Mg target that was large enough to drill. Unable to locate such a target, on Sol 2416 the team steered the rover west, back to the Woodland Bay area to follow up on a thinly/thickly laminated facies first seen at the Flodigarry target on Sol 2356. The rover reached the area on Sol 2420 and repositioned on Sol 2422 for additional observations.

On Sol 2429, the rover headed east with the intent of re-joining the strategic route and addressing the next Glen Torridon campaign objective: to characterize one of the bedrock-capped ridges typical of the smooth, ridged clay-bearing unit near its contact with the overlying fractured clay-bearing unit. The rover arrived at the northern end of Teal ridge (Fig. 9) on Sol 2434, drove southwest parallel to the ridge, then turned southeast to ascend the ridge on Sol 2439 for remote and contact science on the cap rock. The rover was able to ascend the ridge slope at a tilt of up to 23° with only moderate slip. The intricate sedimentary structures in the outcrop were imaged extensively with the Mastcam and a MAHLI dog's eye mosaic. Contact science was executed at the highest rover tilt (23°) of the mission at that time. While at the ridge, a routine measurement of atmospheric methane on Sol 2441 revealed a large increase above the seasonal background level, prompting the team to add a follow-on observation on Sol 2446.

On Sol 2447, the rover backed down the ridge and traversed toward Harlaw, a ridge on the opposite side of a shallow valley. The team placed the strategic route within this valley, dubbed the "visionarium," because MRO-HiRISE images suggested that it would allow observations of ridges on either side for a few hundred meters. The team studied the base of Harlaw with remote and contact science on Sols 2450-52. The rover then ascended partway up the ridge and imaged sedimentary structures using stereo Mastcam and oblique MAHLI views at multiple locations. On Sol 2466, the rover continued south toward an east-west ridge that marked the southern end of the visionarium and was identified as a potential drill site within the fractured clay-bearing unit. Sols 2470-72 were dedicated to contact science on large blocks in the workspace while Sol 2474 was used to image the finely layered face of the southern, ridge-forming outcrop. After drives to the east and up the base of the ridge, the team acquired more images and ChemCam observations of the outcrop on Sol 2476, and contact science on Sol 2477 at East Caithness while at a tilt of 25°.

Drives on Sols 2477 and 2480 brought the rover to the top of the southern outcrop, from where the team selected its next drill target, Glen Etive. A slab was chosen that could fit two drill holes, since this target was a candidate for a SAM wet chemistry experiment. Drilling of Glen Etive completed successfully on Sol 2486, the seven-year anniversary of Curiosity's

landing. Sample material was delivered to CheMin and to SAM. Starting on Sol 2505, activities began being restricted as the mission prepared for its fourth solar conjunction. Two 7-sol plans were made in advance to span the period when commands could not be reliably sent from Earth. The plans included daily Navcam imaging to capture any changes on the surface due to wind, along with regular DAN, REMS, and RAD observations. During conjunction, the science team reviewed the results from Glen Etive and made the decision to drill a second hole to enable the SAM wet chemistry experiment.

After the rover regained communication, on Sol 2523 the team resumed activities related to the first drill hole and the rover successfully drilled a second hole on the same rock slab on Sol 2527. Sample material was delivered to SAM for comparison with the first hole. After review of those results, sample material was delivered to SAM on Sol 2540 for the mission's first wet chemistry experiment on a drilled sample. The team prioritized delivery to SAM before CheMin in order to reduce the risk of running out of sample material for this experiment.

On Sol 2544 Curiosity began its third extended mission (EM3). After spending a few sols finishing the Glen Etive drilling activities, Curiosity resumed its southward drive on Sol 2555. The Culbin Sands megaripple was investigated before and after the rover's wheel created a "scuff," or small trench on Sol 2556. On Sol 2563, the rover drove towards the contact between the fractured clay-bearing unit and the fractured intermediate unit.

The next campaign target was Central butte, an imposing hill that may be an erosional remnant of the Greenheugh pediment to the south (Fig. 9). The butte offered the opportunity to investigate the strata exposed on its steep slopes and observe their sedimentary structures from multiple angles. The rover climbed the northern side of the butte twice from Sol 2568 to 2586. Sol 2582 included an early-morning search for frost with ChemCam, following similar attempts on Sols 2548 and 2565. A drive on Sol 2582 brought the rover to its highest point on the butte.

After finishing up at Central butte, the rover continued west around its northern side and the team set their eyes on Western butte. Along the way, the rover stopped for several sols while operations ceased over the US Thanksgiving Day holiday. The team decided to use this break in driving (as well as the great views of the crater through the relatively clear atmosphere) as an opportunity to capture the largest panorama of the mission: a full 360° stereo Mastcam mosaic that required four sols (2597 and 2600-02) to capture. Western butte is at a higher elevation than Central butte, so its slopes offered the chance to examine both the strata exposed on Central butte as well as higher strata. On Sol 2615, SAM acquired a seasonal measurement of atmospheric methane. A drive on Sol 2618 took the rover on top of the shoulder of Western butte, including a look at dark blocks similar to those that cover its higher slopes.

Sols 2620-30 covered the winter holiday period and included continual REMS and RAD monitoring, as well as another methane measurement on Sol 2626. A drive on Sol 2633 brought the rover to its highest point on Western butte at a tilt of 21°, although it took until Sol 2640 to remove a "wheelie" from the rover's mobility system and enable safe

deployment of the rover's arm, given issues with relay communications and the SAM instrument. While atop Western butte, the rover surveyed the Greenheugh pediment and Gediz Vallis ridge to the south. On Sol 2645 the rover resumed driving, stopping at a trough on the northern side of the shoulder of Western butte. Sols 2649 to 2652 were spent diagnosing an issue with the rover's inertial measurement unit, which tracks the rover's orientation.

As the rover progressed southward toward the scarp that forms the northern margin of the Greenheugh pediment, the team decided to attempt to climb it and reach the upper surface of the pediment capping unit. Although the strategic route to higher on Mount Sharp ascends the pediment further to the south, it was decided that a brief investigation here, including a drill hole, would provide a head start on answering high-priority science questions. In addition, the team wanted to investigate any geochemical trends in the fractured intermediate unit that might be related to the presence of the capping unit. The team added two additional sampling objectives to the campaign: one during the climb as close to the capping unit as possible, and one on the fractured intermediate unit away from the scarp, after coming back down.

On Sol 2662 the rover set a new record by performing contact science at a 26.9° tilt as it neared a narrow bench set into the pediment scarp that offered a place to safely drill. After positioning the rover on the bench on Sol 2664, the drill campaign at Hutton began. Drilling was completed on Sol 2668, followed by sample deliveries to CheMin and twice to SAM on later sols. The team had planned to continue ascending the pediment scarp in a ravine between it and Tower butte, but now saw that it was blocked by deep sand. However, a new path was found that required traversing laterally and then straight up the steep, sandy scarp. After re-debating the merits of ascending the pediment at this point in the mission, the team agreed to make an attempt on the new, more challenging route. Sol 2677 marked the completion of four Mars years of surface operations.

Curiosity began the pediment ascent on Sol 2691. After setting a record for the highest tilt during a drive (31.9°) on Sol 2692, it reached a spot for contact science on exposed rock just below the pediment cap on Sol 2693. A drive on Sol 2695 brought the rover to the top of the Greenheugh pediment and therefore onto an unexplored, major geologic feature. Objectives included investigating two distinct types of capping rock, acquiring images of the pediment surface and Gediz Vallis ridge, and locating a site for drilling. The rover reached the Edinburgh site on Sol 2702 and made a drilling attempt on Sol 2711. The hardness of the rock prevented the drill from reaching full depth, but the team concluded that enough sample material was collected for a limited number of laboratory experiments.

Planning and executing a drilling attempt was an especially significant accomplishment for the team because it was done with all operations team members (not only the science team, but also the JPL-based engineers) participating remotely, through phone and internet connections only, due to the outbreak of the COVID-19 pandemic. The rover descended from the pediment on Sol 2734 and headed toward a site for drilling bedrock in the small valley between Western and Tower buttes. The target Glasgow was reached on Sol 2747

and successfully drilled on Sol 2754. After analyses by CheMin and SAM, the rover drove away on Sol 2780.

With much of the Glen Torridon campaign now complete, including the pediment ascent and extra drill holes, the team decided to prioritize more frequent and longer drives in order to hasten the rover's arrival at the sulfate-bearing unit, another major objective for the mission. This effort was called "Sulfate Unit or Bust," recalling the "Mount Sharp or Bust" push early in the mission. The drive to the east would be interrupted, however, by three waypoints for remote and contact science: the bright bedrock at Bloodstone hill, a blocky unit with a distinct steep slope in MRO-CRISM spectra, and a large, rippled sand sheet named the Sands of Forvie. More significantly, the team decided to conduct a final set of SAM analyses on samples of the clay-bearing unit, including two wet chemistry experiments. The fractured clay-bearing unit was selected for sampling based on previous detections of organic molecules and clay minerals, its relative lack of iron oxides, and the low likelihood that it had experienced alteration related to the pediment given their vertical separation. Geologists on the team used a combination of orbiter- and surface-based mapping to predict where this unit would be found along the rover's path to the east. A significant deviation to the north and to lower elevation was required to reach the area.

The plan on Sol 2784 included a twilight image that placed Mars in the foreground below Earth and Venus in the sky. Drives between Sol 2795 and 2800 ascended the base of Bloodstone hill until excessive slip prevented further progress. The rover resumed driving toward the next drill site on Sol 2802. A patch containing two key fixes to the rover's flight software was installed in steps between Sols 2805 and 2815. On Sol 2827, the SAM experiment performed the first of three dry runs (no sample delivery and no puncturing of the sealed sample cup) for its upcoming tetramethylammonium hydroxide (TMAH) wet chemistry experiment. The purpose of these runs was to test the instrument performance and resource usage throughout the complex experiment in order to reduce the chance of a fault during the actual run, since there are only two TMAH sample cups. Normally such testing would be done using the SAM engineering model, but it was not available due to the pandemic.

On Sol 2829, Curiosity arrived at the Mary Anning drill site (Fig. 9), after the team confirmed that the bedrock matched the characteristics of the fractured clay bearing unit. The target was drilled successfully on Sol 2838. Sample material was delivered to CheMin on Sol 2842 and SAM on Sol 2844, completing Curiosity's eighth year on Mars.

References

F. Abilleira, 2011 Mars Science Laboratory trajectory reconstruction and performance from launch through landing. (JPL Technical Report Server, 2013), https://trs.jpl.nasa.gov/handle/2014/44069. Accessed 28 December 2020

R.E. Arvidson, K.D. Iagnemma, M. Maimone, A.A. Fraeman, F. Zhou, M.C. Heverly, P. Bellutta, D. Rubin, N.T. Stein, J.P. Grotzinger, A.R. Vasavada, Mars Science Laboratory Curiosity

rover megaripple crossings up to sol 710 in Gale Crater. J. Field Robotics 34, 495-518 (2017). https://doi.org/10.1002/rob.21647

S.G. Banham, S. Gupta, D.M. Rubin, J.A. Watkins, D.Y. Sumner, K.S. Edgett, J.P. Grotzinger, K.W. Lewis, L.A. Edgar, K.M. Stack-Morgan, R. Barnes, J.F. Bell III, M.D. Day, R.C. Ewing, M.G.A. Lapotre, N.T. Stein, F. Rivera-Hernandez, A.R. Vasavada, Ancient Martian aeolian processes and palaeomorphology reconstructed from the Stimson formation on the lower slope of Aeolis Mons, Gale crater, Mars. Sedimentology 65, 993-1042 (2018). https://doi.org/10.1111/sed.12469

N.T. Bridges, B.L. Ehlmann, The Mars Science Laboratory (MSL) Bagnold Dunes Campaign, Phase I: Overview and introduction to the special issue. J. Geophys. Res., Planets 123, 3-19 (2018). https://doi.org/10.1002/2017JE005401

A. Chen, M. Greco, T. Martin-Mur, B. Portock, A. Steltzner, Approach and entry, descent, and landing operations for Mars Science Laboratory. J. Spacecraft Rockets 51, 1004-1013 (2014a). https://doi.org/10.2514/1.A32632

A. Chen, A. Cianciolo, A.R. Vasavada, C. Karlgaard, J. Barnes, B. Cantor, D. Kass, S. Rafkin, D. Tyler, Reconstruction of atmospheric properties from Mars Science Laboratory entry, descent, and landing. J. Spacecraft Rockets 51, 1062-1075 (2014b). https://doi.org/10.2514/1.A32708

A.D. Cianciolo, B. Cantor, J. Barnes, D. Tyler Jr., S. Rafkin, A. Chen, D. Kass, M. Mischna, A.R. Vasavada, Atmospheric assessment for Mars Science Laboratory entry, descent, and landing operations. (NASA Technical Reports Server, 2013), https://ntrs.nasa.gov/citations/20140001381. Accessed 29 December 2020

A.A. Fraeman, L.A. Edgar, E.B. Rampe, L.M. Thompson J. Frydenvang , C.M. Fedo, J.G. Catalano, W.E. Dietrich, T.S.J. Gabriel, A.R. Vasavada, J.P. Grotzinger, J. L'Haridon, N. Mangold, V.Z. Sun, C.H. House, A.B. Bryk, C. Hardgrove, S. Czarnecki , K.M. Stack, R.V. Morris, R.E. Arvidson, S.G. Banham, K.A. Bennett, J.C. Bridges, C.S. Edwards, W.W. Fischer, V.K. Fox, S. Gupta, B.H.N. Horgan, S.R. Jacob, J.R. Johnson, S.S. Johnson, D.M. Rubin, M.R. Salvatore, S.P. Schwenzer, K.L. Siebach, N.T. Stein, S. Turner, D.F. Wellington, R.C. Wiens, A.J. Williams, G. David, G.M. Wong, Evidence for a diagenetic origin of Vera Rubin Ridge, Gale Crater, Mars: Summary and synthesis of Curiosity's exploration campaign. J. Geophys. Res., Planets 125, e2020JE006527 (2020). https://doi.org/10.1029/2020JE006527

C.D. Karlgaard, P. Kutty, Mars Science Laboratory Entry Atmospheric Data System trajectory and atmosphere reconstruction. J. Spacecraft Rockets 51, 1029-1047 (2014a). https://doi.org/10.2514/1.A32770

M.G.A. Lapotre, E.B. Rampe, Curiosity's investigation of the Bagnold Dunes, Gale crater: Overview of the two-phase scientific campaign and the introduction to the special collection. Geophys. Res. Lett. 45, 10200-10210 (2018). https://doi.org/10.1029/2018GL079032

T.J. Martin-Mur, G.L. Kruizinga, P.D. Burkhart, F. Abilleira, M.C. Wong, J.A. Kangas, Mars Science Laboratory interplanetary navigation. J. Spacecraft Rockets 51, 1014-1028 (2014). https://doi.org/10.2514/1.A32631

M.S. Rice, S. Gupta, A.H. Treiman, K.M. Stack, F. Calef, L.A. Edgar, J. Grotzinger, N. Lanza, L. Le Deit, J. Lasue, K.L. Siebach, A. Vasavada, R.C. Wiens, J. Williams, Geologic overview of the Mars Science Laboratory rover mission at The Kimberley, Gale crater, Mars. J. Geophys. Res., Planets 122, 2-20 (2017). https://doi.org/10.1002/2016JE005200

K.M. Stack, C.S. Edwards, J.P. Grotzinger, S. Gupta, D.Y. Sumner, F.J. Calef III, L.A. Edgar, K.S. Edgett, A.A. Fraeman, S.R. Jacob, L. Le Deit, K.W. Lewis, M.S. Rice, D. Rubin, R.M.E. Williams, K.H. Williford, Comparing orbiter and rover image-based mapping of an ancient sedimentary environment, Aeolis Palus, Gale crater, Mars. Icarus 280, 3-21 (2016). https://doi.org/10.1016/j.icarus.2016.02.024

K.M. Stack, J.P. Grotzinger, M.P. Lamb, S. Gupta, D.M. Rubin, L.C. Kah, L.A. Edgar, D.M. Fey, J.A. Hurowitz, M. McBride, F. Rivera-Hernández, D.Y. Sumner, J.K. Van Beek, R.M.E. Williams, R.A. Yingst, Evidence for plunging river plume deposits in the Pahrump Hills member of the Murray formation, Gale crater, Mars. Sedimentology 66, 1768-1802 (2019). https://doi.org/10.1111/sed.12558

A.R. Vasavada, A. Chen, J.R. Barnes, P.D. Burkhart, B.A. Cantor, A.M. Dwyer-Cianciolo, R.L. Fergason, D.P. Hinson, H.L. Justh, D.M. Kass, S.R. Lewis, M.A. Mischna, J.R. Murphy, S.C.R. Rafkin, D. Tyler, P.G. Withers, Assessment of environments for Mars Science Laboratory entry, descent, and surface operations. Space Sci. Rev. 170, 793-835 (2012). https://doi.org/10.1007/s11214-012-9911-3

A.R. Vasavada, J.P. Grotzinger, R.E. Arvidson, F.J. Calef, J.A. Crisp, S. Gupta, J. Hurowitz, N. Mangold, S. Maurice, M.E. Schmidt, R.C. Wiens, R.M.E. Williams, R.A. Yingst, Overview of the Mars Science Laboratory mission: Bradbury Landing to Yellowknife Bay and beyond. J. Geophys. Res., Planets 119, 1134-1161 (2014). https://doi.org/10.1002/2014JE004622

D.W. Way, J.L. Davis, J.D. Shidner, Assessment of the Mars Science Laboratory entry, descent, and landing simulation. (NASA Technical Reports Server, 2013), https://ntrs.nasa.gov/citations/20130010129. Accessed 01 January 2021

R.M.E. Williams, M.C. Malin, K.M. Stack, D.M. Rubin, Assessment of Aeolis Palus stratigraphic relationships based on bench-forming strata in the Kylie and the Kimberley regions of Gale crater, Mars. Icarus 309, 84-104 (2018). https://doi.org/10.1016/j.icarus.2018.02.028

Table 1 Mars and Earth dates (UTC) of key mission milestones

Milestone	Sol	Date Planned	Date Executed (12:00 LMST)
**Landing**	**0**	**--**	**August 6, 2012**
Sol 500	500	December 31, 2013	January 1, 2014
**Start of EM1**	**767**	**October 1, 2014**	**October 3, 2014**
Sol 1000	1000	May 29, 2015	May 30, 2015
**Start of EM2**	**1478**	**October 1, 2016**	**October 3, 2016**
Sol 1500	1500	October 24, 2016	October 26, 2016
Sol 2000	2000	March 22, 2018	March 23, 2018
Sol 2500	2500	August 17, 2019	August 18, 2019
**Start of EM3**	**2544**	**October 1, 2019**	**October 3, 2019**
Sol 3000	3000	January 12, 2021	January 13, 2021

Fig. 1 Map of Curiosity's traverse as of early 2021. Image credit: NASA/JPL-Caltech/U. of Arizona

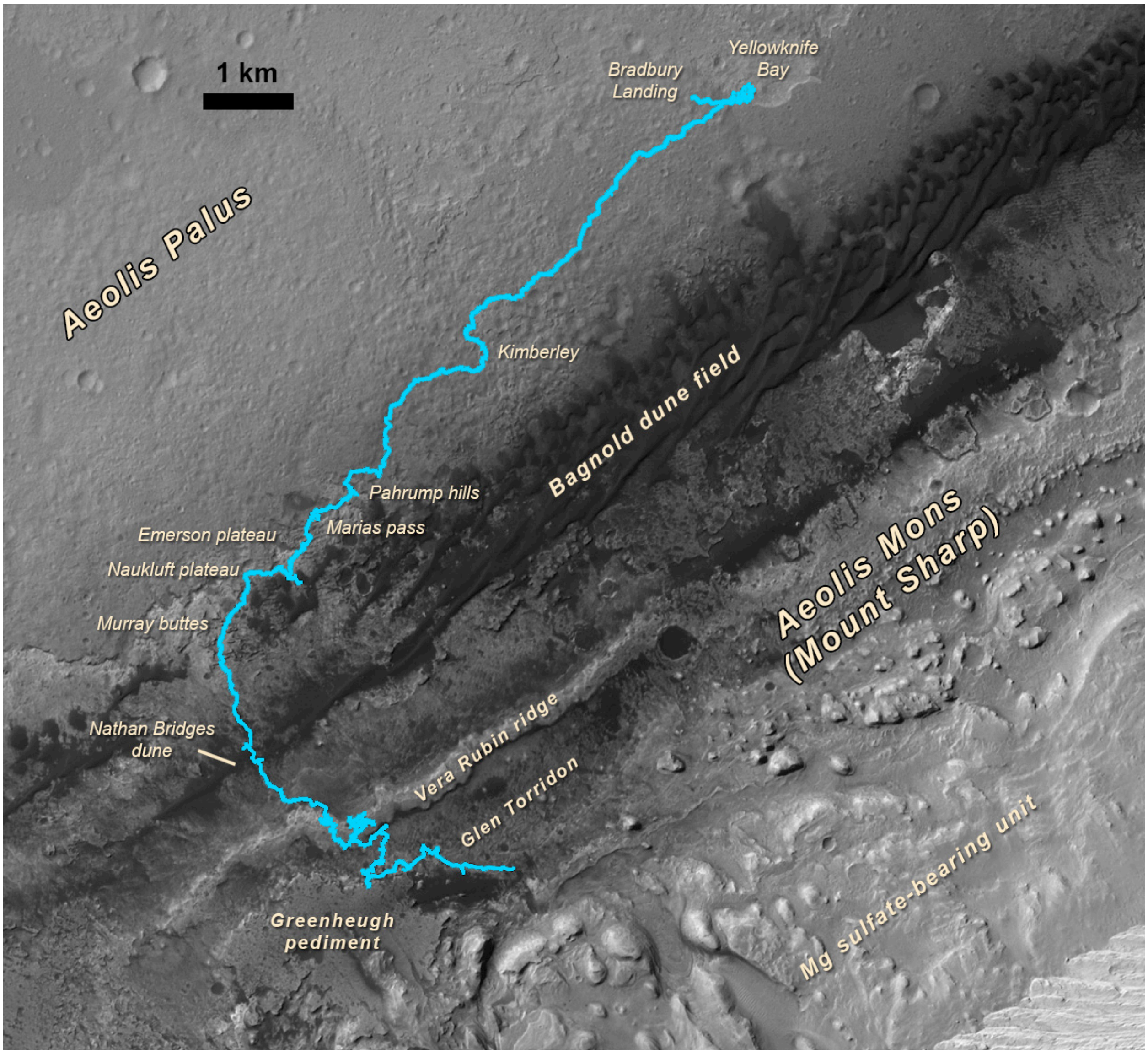

Fig. 2 Map of Curiosity's traverse (Sols 511-634) from Dingo Gap to the Kimberley. In Figs. 2 through 9, geographic features are in italics, science observation targets are in bold, the sols that the rover arrived at parking spots (dots) are in white, north is up, and a scale bar is located in the upper left corner. Image credit: NASA/JPL-Caltech/U. of Arizona

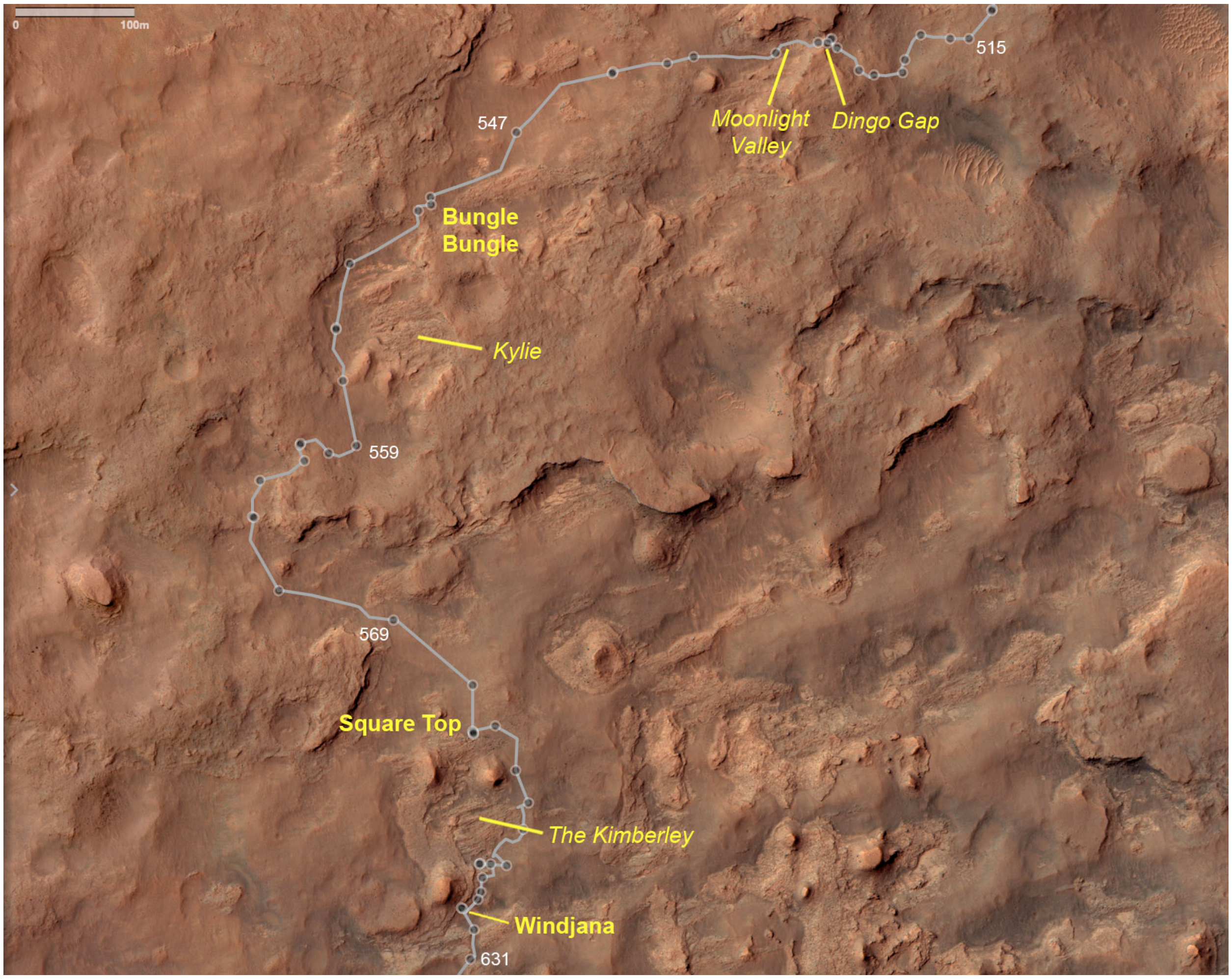

Fig. 3 Map of Curiosity's traverse (Sols 690-952) approaching and at Pahrump hills

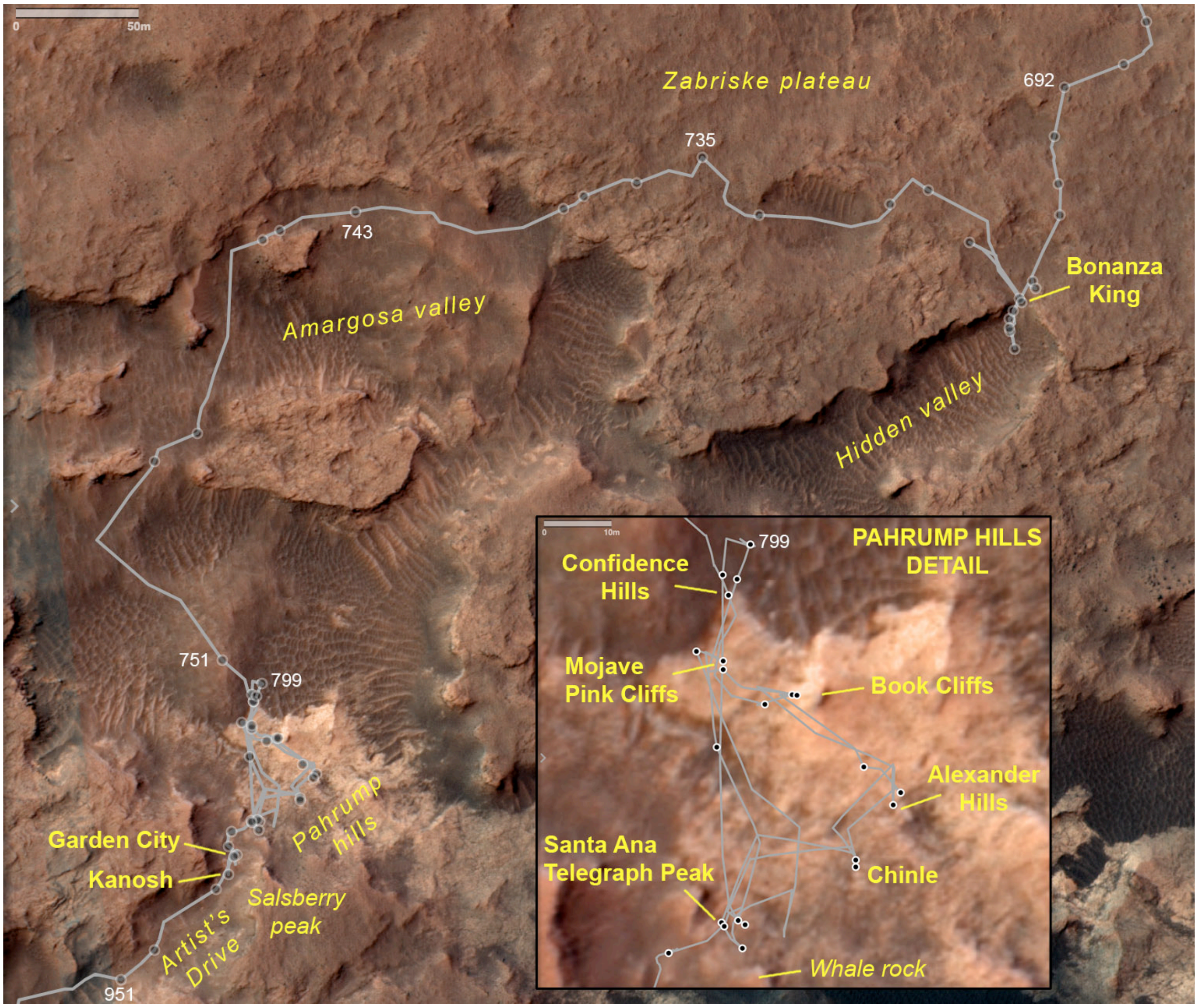

Fig. 4 Map of Curiosity's traverse (Sols 960-1074) approaching and at Marias pass

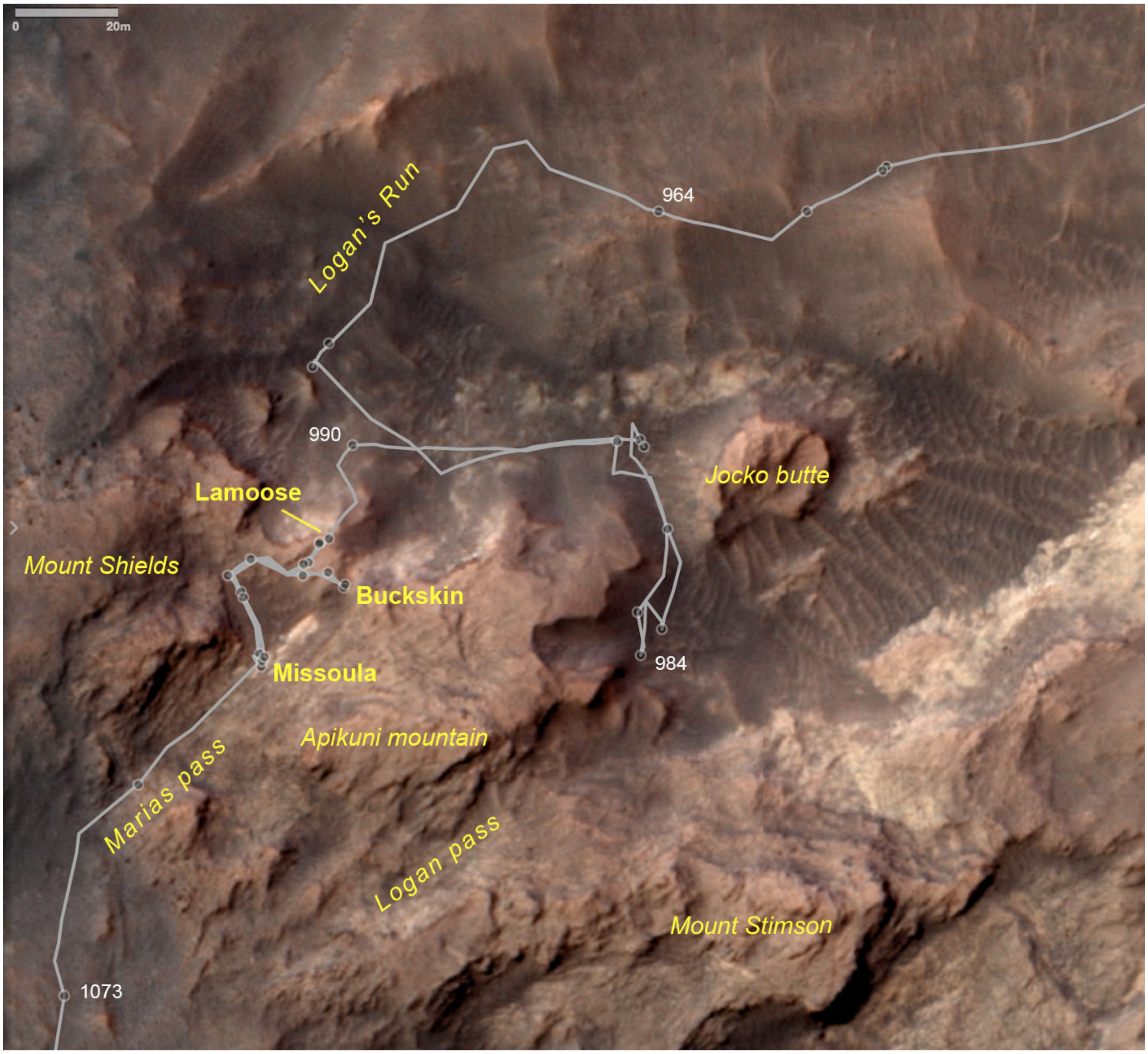

Fig. 5 Map of Curiosity's traverse (Sols 1073-1385) across Emerson plateau, Bagnold dune field (first crossing), and Naukluft plateau

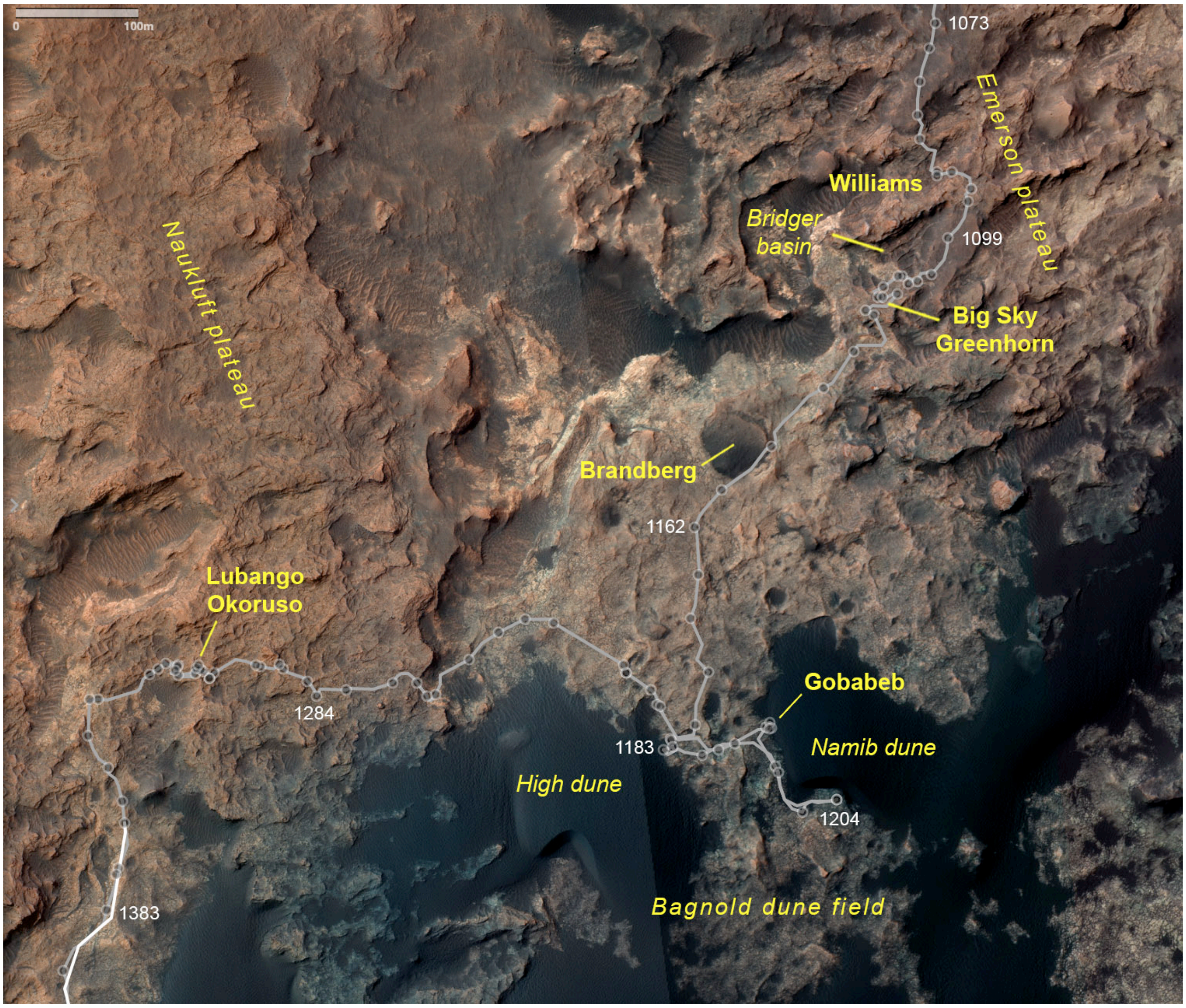

Fig. 6 Map of Curiosity's traverse (Sols 1162-1509) highlighting exploration near Murray buttes

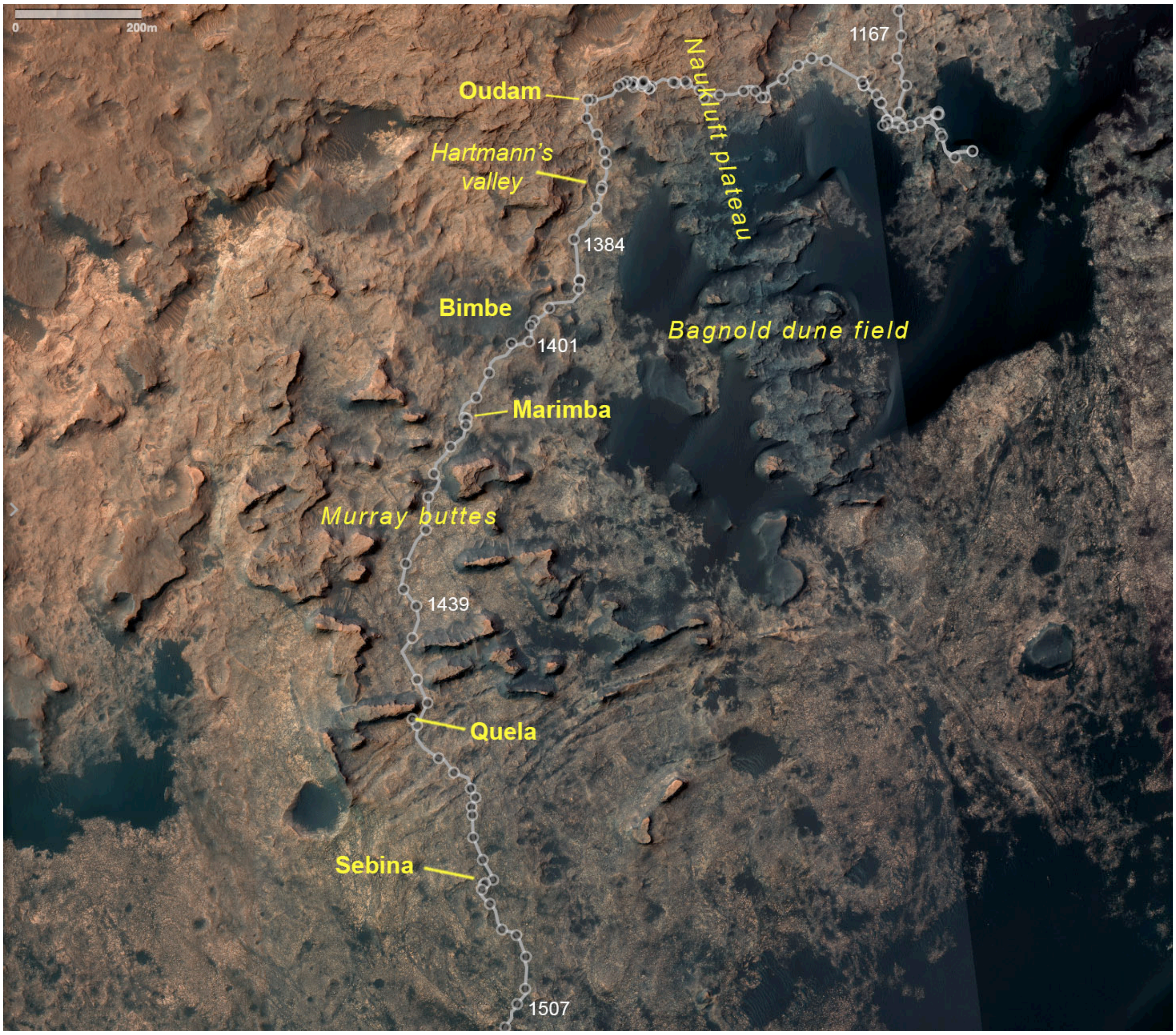

Fig. 7 Map of Curiosity's traverse (Sols 1508-1680) through Bagnold dune field (second crossing)

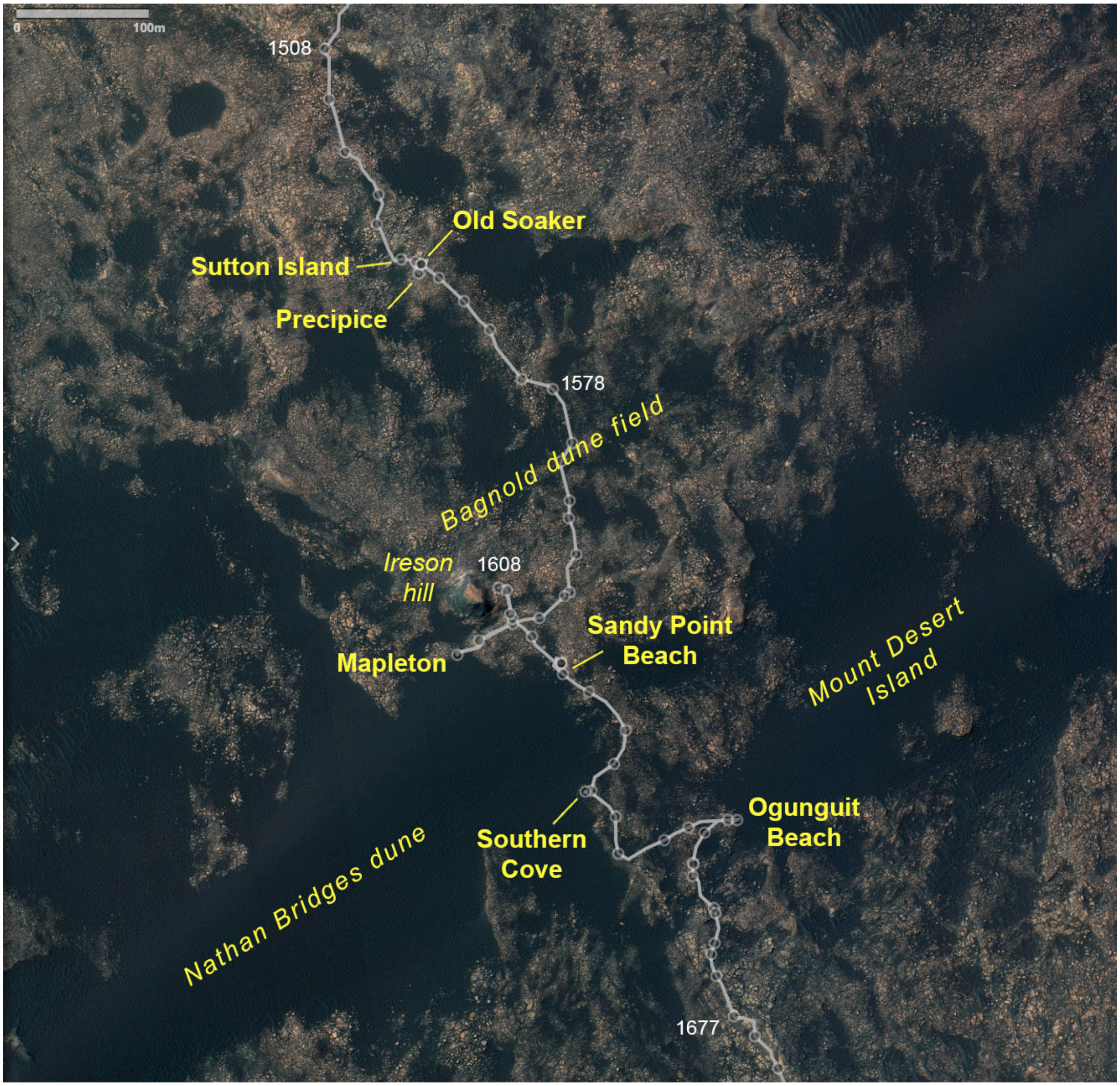

Fig. 8 Map of Curiosity's traverse across (Sols 1782-2602) highlighting exploration of Vera Rubin ridge

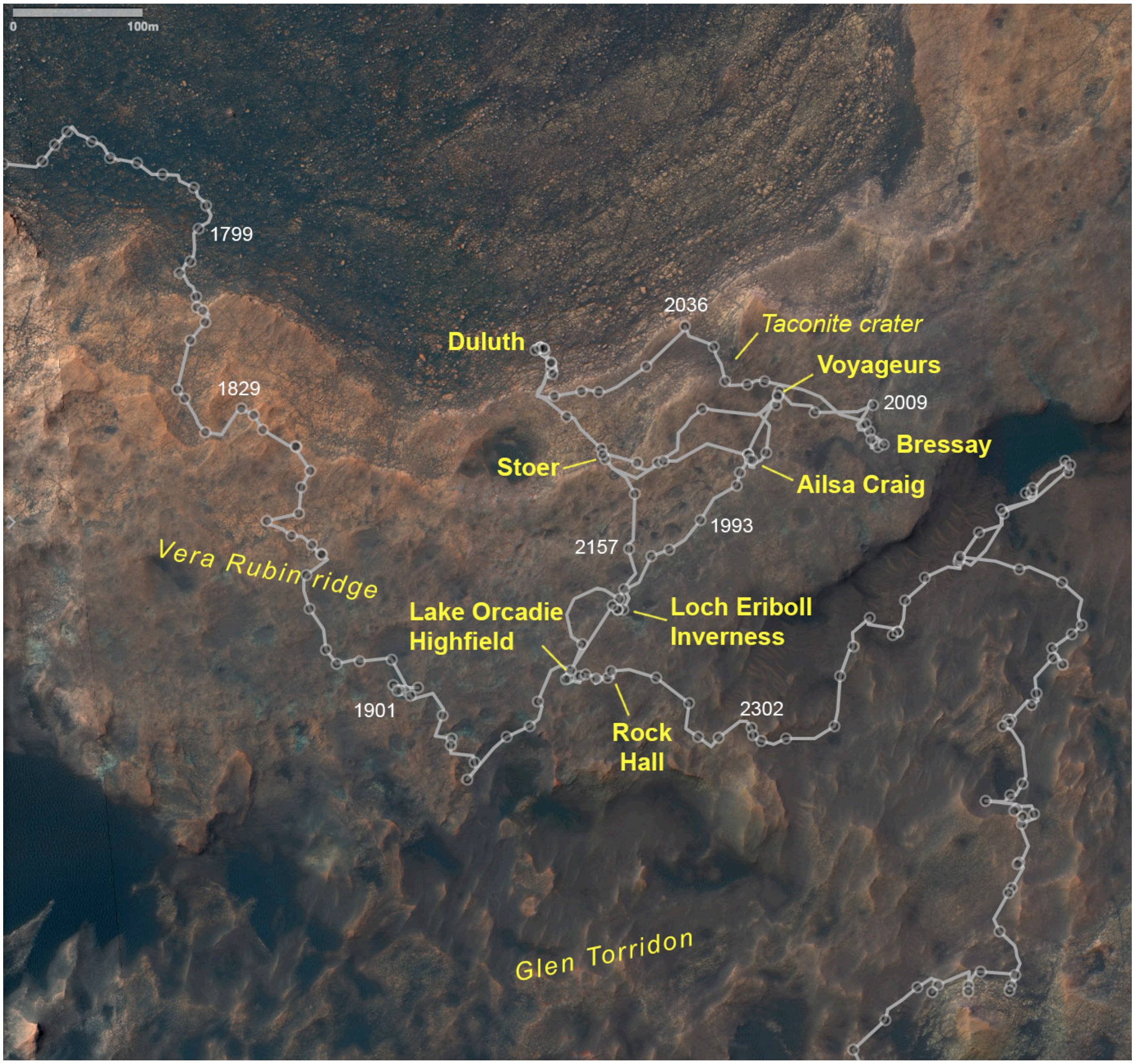

Fig. 9 Map of Curiosity's traverse (Sols 1962-2936) highlighting exploration of Glen Torridon

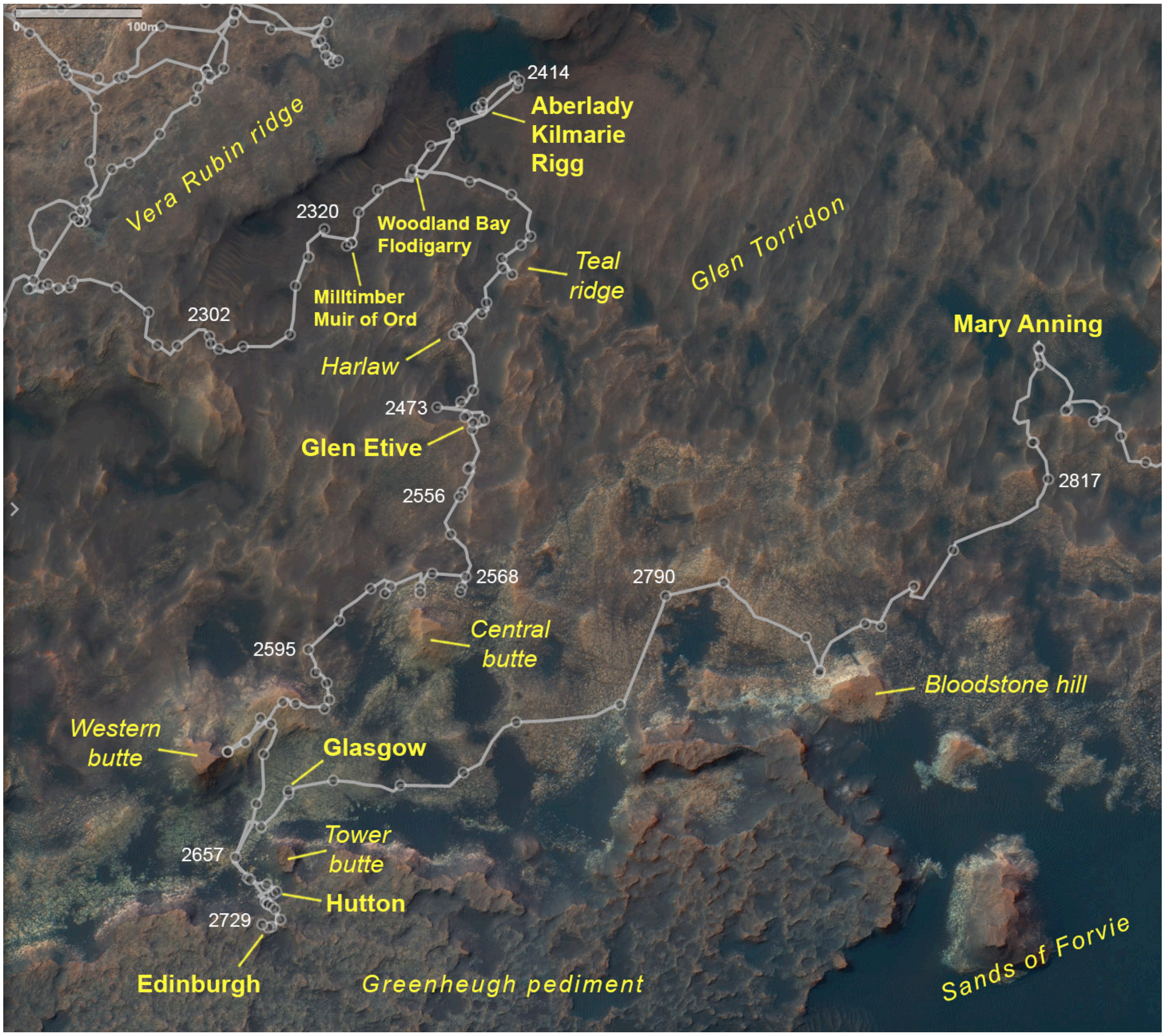

Supplement: Supplementary file 1 — Mars Science Laboratory Mission Narrative through Sol 2844 (Eight Earth Years) (PDF 4.8 MB) [file 11214_2022_882_MOESM1_ESM.pdf]
