# Supplementary material for: Mission Overview and Scientific Contributions from the Mars Science Laboratory Curiosity Rover After Eight Years of Surface Operations
Source: Space Sci Rev. 2022 Apr 5;218(3):14. doi: 10.1007/s11214-022-00882-7 (PMC8981195; doi:10.1007/s11214-022-00882-7)
Supplement: Supplementary file 2 — Mission Science Objectives, Functional Requirements, and Success Criteria (PDF 69 kB) [file 11214_2022_882_MOESM2_ESM.pdf]

**ONLINE RESOURCE 2**

# Mission Overview and Scientific Contributions from the Mars Science Laboratory Curiosity Rover After Eight Years of Surface Operations

*Space Science Reviews*

Ashwin R. Vasavada

Jet Propulsion Laboratory, California Institute of Technology, Pasadena, California, USA

Email: ashwin.r.vasavada@jpl.nasa.gov

**Mission Science Objectives, Functional Requirements, and Success Criteria**

## 1. Science Objectives

Overall: Quantitatively assess past and present habitable environments at Gale crater.

1. Characterize geological features contributing to deciphering geological history and the processes that have formed or modified rocks and regolith, with emphasis on the role of water.
2. Determine the mineralogy and chemical composition of surface and near-surface materials, including an inventory of elements such as C, H, N, O, P, S, etc. known to be building blocks for life.
3. Determine energy sources that could be used to sustain biological processes.
4. Characterize organic compounds and potential biomarkers in rocks and regolith.
5. Identify potential biosignatures (chemical, textural, isotopic) in rocks and regolith.
6. Identify and quantitatively assess "taphonomic windows" for organic carbon (subset of habitable environments also capable of preserving organic compounds) through refined models for primary facies distributions and diagenesis.
7. Characterize the local environment, including basic meteorology, the state and cycling of water and $CO_2$, and the near-surface distribution of hydrogen.
8. Determine the stable isotopic and noble gas composition of the present-day atmosphere and the stable isotopic composition of H, C, N, O, S, and Cl in volatile compounds thermally released from solid samples.
9. Explore and characterize major environmental transitions recorded in the geology and geochemistry of the foothills of Mount Sharp and adjacent plains.
10. Characterize the broad spectrum of surface radiation, including galactic cosmic radiation, solar particle events, and secondary neutrons.

## 2. Functional Requirements and Status

From Appendix B-8 to NASA's Mars Exploration Program Plan, July 2006.

| Functional Requirement | Status |
|---|---|
| 1. The project shall launch a mobile surface mission to Mars during the 2009 opportunity from the Eastern Test Range. | Launched 11/26/11 after slip of one launch opportunity. |
| 2. The project shall deliver a flight system to be launched on a launch vehicle competitively selected under the NASA Launch Services contract compatible with both Atlas 5 and Delta IV intermediate-class launch vehicles. | Launched on Atlas 5. |
| 3. The project system shall be able to land at altitudes of up to +1.0 km relative to the MOLA areoid. The threshold for altitude capability is 0 km. | Landed at -4.5 km elevation. System design met threshold capability of 0 km. |
| 4. The project system shall be capable of landing and operating at sites between 45°N and 45°S latitude selected as late as one year before launch without compromising overall mission safety. The threshold for latitude capability is between 30°N and 30°S. | Landed at 4.6°S. System design met threshold capability of 30°S to 30°N. |
| 5. The project system shall be able to land with an error of 10 km or less radially from a designated point on the surface of Mars (excluding any uncontrolled effects of winds during parachute descent) utilizing a guided atmospheric entry. The threshold for landing error capability is less than 20 km radially from a designated point on the surface of Mars. | Landed 2.4 km (<1 sigma) from designated point. System design achieved 9.5 x 3.5 km (semi-axes) 3-sigma ellipse. |
| 6. The project system shall provide data communication throughout critical events, at a rate sufficient to determine the state of the spacecraft in support of fault reconstruction, to relay assets provided by the Mars Program or to the Deep Space Network. | Near-continuous data transmission demonstrated during launch, entry, descent, and landing (EDL). |
| 7. The project system shall acquire scientific data about the rover's local region and conduct in-situ analysis with the rover and its scientific payload on the surface of Mars for at least one martian year (669 sols). The threshold for mission duration is one-half of a martian year (335 sols). | Active for >2844 sols. |
| 8. The project shall deliver to Mars a rover with the capability of a total traverse path length of at least 20 km. The threshold for total traverse capability is 10 km path length. | > 23 km traverse distance at Sol 2844. |
| 9. The project system shall be able to select, acquire, process, distribute, and analyze at least 74 samples of rock, rock fragments, and/or regolith. The threshold capability is at least 28 samples. | Met threshold. As of Sol 2844, Curiosity has acquired and analyzed 33 samples. |
| 10. The project shall conduct near real-time public release of imagery and other science/technology data via the Internet, and will provide regular releases for public information purposes. | Raw imagery released within 1 day of receipt. Regular public releases of news and processed imagery. |
| 11. The project shall archive copies of all verified, validated, and calibrated data acquired by the mission to the Planetary Data System within six months after its receipt on Earth. | Archive data volumes delivered to the Planetary Data System ranging in age from 3 to 7 months per agreement with NASA HQ. |

## 3. Mission Success Criteria and Status

From Appendix B-8 to NASA's Mars Exploration Program Plan, July 2006.

| Mission Success Criterion | Status |
|---|---|
| 1. Land safely on the surface of Mars. | Successfully landed Aug. 5, 2012 |
| 2. Provide mobility capability on the surface of Mars. | Successfully drove to multiple science-specified sites. Reached edge of landing ellipse with total traverse path of ~8 km at end of Prime Mission. |
| 3. Assess the biological potential of at least one target environment, including the chemical and mineralogical analysis of multiple samples. | At Yellowknife Bay, Curiosity characterized geological features, determined the mineralogical/chemical composition and characterized organic compounds from multiple portions from two acquired samples of rock powder, searched for potential biosignatures, and characterized the broad spectrum of surface radiation. |
| 4. Characterize the geology of the landing region at all available spatial scales. | Curiosity has used its Mastcam, ChemCam remote micro-imager, MAHLI hand lens imager, and MARDI nadir camera to characterize geology at scales of tens of km to 15 micrometers. |
| 5. Investigate planetary processes of relevance to past habitability. | Curiosity has determined the stable isotopic and noble gas composition of the present-day atmosphere with a view to atmospheric evolution. Analysis of D/H in water evolved from solid samples with a known geologic context provided a time marker for atmospheric loss. Geological, chemical, and mineralogical analyses of Yellowknife Bay and other sites have characterized fluvial processes, environmental conditions, weathering processes, and other processes related to past habitability. |
| 6. Collect at least 75% of the science data that is transmitted by the rover. | Science data return to Earth is essentially lossless. <5% of data required re-transmission. |
| 7. Provide for regular public release of imagery and other science data via the Internet as well as regular releases for public information purposes. | Public website quickly posts raw images and press releases with associated science images and data products for key events and discoveries. |
| 8. Archive a copy of verified, validated, and calibrated data acquired by the mission to the Planetary Data System within six months after receipt of the data on Earth. | With a few exceptions that have now been remedied, all verified, validated, and calibrated data acquired by instruments during the prime mission were archived with an age range of three to seven months after receipt of the data on Earth. |
