# Supplementary material for: Mission Overview and Scientific Contributions from the Mars Science Laboratory Curiosity Rover After Eight Years of Surface Operations
Source: Space Sci Rev. 2022 Apr 5;218(3):14. doi: 10.1007/s11214-022-00882-7 (PMC8981195; doi:10.1007/s11214-022-00882-7)
Supplement: Supplementary file 3 — Summary Table of Rover Traverses (PDF 241 kB) [file 11214_2022_882_MOESM3_ESM.pdf]

ONLINE RESOURCE 3

Mission Overview and Scientific Contributions from the Mars Science Laboratory Curiosity Rover After Eight Years of Surface Operations

Space Science Reviews

Ashwin R. Vasavada

Jet Propulsion Laboratory, California Institute of Technology, Pasadena, California, USA

Email: ashwin.r.vasavada@jpl.nasa.gov

Summary Table of Rover Traverses

Sol	Distance (m)	Total Odometry (m)	Azimuth (degrees CW from North)	Elevation (m)	Planetocentric Latitude	East Longitude
0	0.0	0.0	111.5	-4501.1	-4.589467	137.441633
16	7.0	7.0	228.0	-4501.1	-4.589465	137.441734
21	4.9	11.9	195.3	-4501.4	-4.589401	137.441686
22	15.1	27.0	64.6	-4502.4	-4.589403	137.441892
24	21.5	48.5	116.5	-4502.6	-4.589447	137.442181
26	29.8	78.3	45.2	-4503.0	-4.589750	137.442476
29	30.6	108.9	239.8	-4503.6	-4.590122	137.442894
38	32.3	141.2	90.6	-4504.6	-4.590244	137.443302
39	21.7	162.9	85.5	-4504.8	-4.590319	137.443663
40	37.2	200.2	89.3	-4504.5	-4.590037	137.444178
41	26.9	227.0	89.3	-4505.0	-4.589847	137.444581
42	32.0	259.1	109.8	-4507.2	-4.590042	137.445051
43	30.0	289.1	82.0	-4507.9	-4.590477	137.445310
45	4.5	293.5	124.3	-4508.1	-4.590435	137.445348
48	41.7	335.2	171.3	-4510.4	-4.590301	137.446000
49	30.7	366.0	89.2	-4512.1	-4.590306	137.446506
50	48.9	414.9	45.2	-4515.5	-4.590176	137.447304
52	36.3	451.2	99.8	-4516.6	-4.590062	137.447900
53	2.1	453.3	81.4	-4516.6	-4.590066	137.447940
55	23.5	476.8	0.2	-4517.9	-4.590063	137.448297
56	5.9	482.7	282.5	-4517.9	-4.590017	137.448351
57	1.8	484.5	282.8	-4517.9	-4.590020	137.448339
59	5.5	490.0	276.7	-4517.8	-4.589996	137.448342
100	1.9	491.9	272.3	-4517.9	-4.590022	137.448310
102	25.3	517.2	87.8	-4518.3	-4.589948	137.448695
111	1.9	519.1	56.2	-4518.2	-4.589922	137.448676
120	34.6	553.7	45.6	-4518.3	-4.590443	137.448830
121	24.2	577.9	23.9	-4518.7	-4.590282	137.449107
122	1.0	578.9	65.3	-4518.7	-4.590275	137.449120
123	19.4	598.3	36.9	-4519.8	-4.590054	137.449349
124	14.0	612.3	32.9	-4520.0	-4.589866	137.449277
125	26.1	638.4	269.8	-4520.9	-4.589637	137.449331
127	32.8	671.2	45.4	-4521.3	-4.589231	137.449383
130	5.6	676.8	324.1	-4521.0	-4.589137	137.449388
133	21.7	698.5	214.6	-4521.0	-4.589463	137.449258
147	3.0	701.5	202.2	-4520.9	-4.589506	137.449235
151	0.7	702.2	204.3	-4520.8	-4.589516	137.449231
152	2.4	704.6	200.7	-4520.5	-4.589552	137.449223
159	1.4	705.9	223.8	-4520.7	-4.589535	137.449238
162	9.0	714.9	220.4	-4520.5	-4.589451	137.449141
163	1.9	716.8	221.7	-4520.4	-4.589476	137.449120
164	3.2	720.1	200.8	-4520.5	-4.589467	137.449136
166	3.3	723.4	165.7	-4520.5	-4.589485	137.449129
272	3.1	726.5	198.7	-4520.4	-4.589467	137.449116
274	0.7	727.2	200.1	-4520.4	-4.589479	137.449112
295	6.3	733.5	40.9	-4520.3	-4.589502	137.449112
297	19.8	753.3	233.6	-4520.7	-4.589571	137.449275
299	8.4	761.7	232.4	-4519.9	-4.589661	137.449168
301	12.0	773.7	244.4	-4519.3	-4.589745	137.448996

302	2.7	776.5	215.1	-4519.0	-4.589779	137.448964
307	31.8	808.2	151.1	-4519.2	-4.590148	137.449275
308	22.0	830.2	234.8	-4518.3	-4.590378	137.449050
309	1.8	832.0	270.0	-4518.2	-4.590374	137.449024
313	9.3	841.3	104.8	-4518.2	-4.590455	137.448910
317	6.8	848.1	160.3	-4518.2	-4.590507	137.448813
324	18.0	866.1	166.6	-4518.4	-4.590203	137.448728
327	40.0	906.2	241.0	-4516.7	-4.590189	137.448117
329	41.1	947.3	244.3	-4515.4	-4.590202	137.447535
331	28.0	975.3	221.1	-4513.9	-4.590515	137.447213
333	15.5	990.8	197.7	-4512.9	-4.590705	137.447088
335	38.2	1029.0	218.9	-4511.0	-4.591184	137.446737
336	32.9	1062.0	239.9	-4509.1	-4.591452	137.446288
337	37.7	1099.6	242.1	-4506.9	-4.591769	137.445740
338	34.1	1133.7	237.6	-4504.9	-4.592082	137.445290
340	100.3	1234.0	242.6	-4504.0	-4.593047	137.444250
342	62.4	1296.4	206.7	-4501.2	-4.593831	137.443652
343	33.7	1330.1	241.4	-4500.1	-4.593837	137.443104
344	70.1	1400.2	240.5	-4499.7	-4.594072	137.441967
345	70.2	1470.3	235.2	-4499.7	-4.594665	137.440999
347	60.1	1530.5	210.2	-4499.4	-4.595182	137.440156
349	70.2	1600.6	209.1	-4500.2	-4.596308	137.439878
351	85.1	1685.7	235.0	-4501.4	-4.596859	137.438602
354	57.1	1742.8	235.0	-4500.5	-4.596819	137.438280
356	50.0	1792.8	205.1	-4501.0	-4.596951	137.437010
358	35.0	1827.9	194.1	-4500.6	-4.597514	137.436924
361	73.1	1900.9	203.0	-4501.4	-4.598564	137.436402
363	84.6	1985.5	215.9	-4502.0	-4.598978	137.435081
365	26.5	2012.0	225.1	-4502.1	-4.599240	137.434730
369	70.1	2082.1	214.9	-4503.4	-4.599891	137.433740
370	81.6	2163.7	209.7	-4503.3	-4.600005	137.432560
371	110.2	2273.8	204.1	-4503.3	-4.600243	137.431071
372	40.1	2313.9	215.6	-4502.1	-4.600518	137.430491
374	42.9	2356.8	207.9	-4501.7	-4.601090	137.430643
376	43.0	2399.8	210.8	-4503.5	-4.601642	137.430208
377	61.3	2461.1	221.1	-4502.4	-4.602228	137.429607
378	90.1	2551.2	222.6	-4503.2	-4.603365	137.428661
379	15.1	2566.3	230.1	-4502.5	-4.603459	137.428428
383	42.4	2608.8	224.6	-4503.5	-4.603925	137.427912
385	141.5	2750.3	186.3	-4500.9	-4.606005	137.427272
388	24.3	2774.5	179.4	-4501.7	-4.606251	137.427059
390	75.2	2849.7	160.5	-4505.9	-4.607074	137.426279
392	2.7	2852.5	141.1	-4505.7	-4.607115	137.426291
396	9.8	2862.3	173.9	-4505.1	-4.607217	137.426275
402	22.8	2885.0	260.1	-4504.9	-4.607204	137.425896
403	67.9	2953.0	220.7	-4504.7	-4.608044	137.425251
404	64.3	3017.2	262.9	-4502.8	-4.608585	137.424417
406	72.6	3089.9	210.9	-4503.5	-4.609542	137.423910
409	70.6	3160.4	186.1	-4503.2	-4.610645	137.424049
410	32.9	3193.4	214.5	-4502.1	-4.611086	137.423736
412	97.3	3290.7	217.8	-4502.9	-4.611823	137.422374
413	79.8	3370.5	160.2	-4500.4	-4.613046	137.422372
417	58.5	3429.0	175.4	-4498.8	-4.613358	137.421488
419	125.8	3554.8	179.7	-4497.9	-4.615214	137.420769
422	70.0	3624.7	179.3	-4497.6	-4.616304	137.420416
424	94.4	3719.2	205.0	-4498.0	-4.617718	137.420161

426	47.8	3767.0	205.6	-4496.6	-4.618001	137.419445
429	46.4	3813.4	200.7	-4496.5	-4.618455	137.418884
431	71.5	3884.9	210.1	-4495.6	-4.619215	137.418180
433	93.4	3978.3	221.0	-4492.4	-4.619987	137.417152
436	93.5	4071.8	200.2	-4492.8	-4.621081	137.416146
437	31.9	4103.7	200.9	-4492.1	-4.621534	137.416014
438	48.8	4152.6	211.9	-4494.1	-4.621985	137.415335
439	25.5	4178.1	220.1	-4494.3	-4.622291	137.415058
440	4.7	4182.8	192.6	-4493.7	-4.622335	137.415000
453	46.9	4229.7	209.7	-4492.2	-4.622509	137.414675
454	103.3	4333.0	211.8	-4492.9	-4.622435	137.413126
455	87.2	4420.2	141.3	-4492.9	-4.623129	137.412360
465	50.3	4470.5	206.4	-4492.7	-4.623464	137.411841
470	74.0	4544.5	209.5	-4491.0	-4.624251	137.411061
472	50.0	4594.5	215.8	-4489.7	-4.624878	137.410566
474	8.7	4603.2	204.7	-4490.1	-4.624881	137.410477
477	5.3	4608.4	186.2	-4489.8	-4.624957	137.410437
488	0.5	4609.0	186.6	-4489.7	-4.624948	137.410444
490	1.3	4610.2	186.7	-4489.8	-4.624968	137.410437
494	20.2	4630.4	211.6	-4488.8	-4.625259	137.410284
504	23.5	4653.9	210.4	-4489.7	-4.625275	137.409923
506	25.1	4679.0	215.0	-4489.0	-4.625550	137.409625
508	9.5	4688.5	198.6	-4489.2	-4.625699	137.409575
511	28.5	4717.0	189.7	-4487.8	-4.626121	137.409568
513	1.2	4718.2	190.5	-4487.8	-4.626143	137.409561
515	30.3	4748.6	211.2	-4486.7	-4.626535	137.409230
518	15.7	4764.3	225.7	-4487.3	-4.626539	137.408967
519	25.3	4789.6	225.7	-4489.5	-4.626482	137.408553
520	26.0	4815.5	209.9	-4489.9	-4.626841	137.408323
521	11.2	4826.8	171.1	-4489.4	-4.627014	137.408288
524	24.1	4850.9	219.8	-4487.0	-4.627050	137.407881
526	15.0	4865.9	219.8	-4487.3	-4.626986	137.407666
527	28.2	4894.1	217.9	-4487.6	-4.626670	137.407362
528	15.7	4909.8	229.0	-4486.8	-4.626551	137.407263
532	1.0	4910.8	228.4	-4486.7	-4.626539	137.407279
533	7.1	4918.0	283.2	-4486.9	-4.626598	137.407214
535	7.0	4925.0	271.4	-4487.8	-4.626590	137.407094
538	41.1	4966.1	217.6	-4489.3	-4.626746	137.406492
540	73.1	5039.2	214.9	-4487.6	-4.626792	137.405318
542	22.8	5061.9	215.7	-4487.0	-4.626894	137.404949
545	47.0	5109.0	224.8	-4488.4	-4.627012	137.404176
546	1.2	5110.2	224.6	-4488.4	-4.627025	137.404164
547	100.3	5210.4	131.6	-4491.0	-4.627868	137.402797
548	100.1	5310.5	135.8	-4487.3	-4.628784	137.401576
549	7.0	5317.5	182.4	-4487.2	-4.628889	137.401586
550	15.9	5333.3	184.2	-4486.7	-4.628981	137.401401
552	79.2	5412.5	229.0	-4487.8	-4.629728	137.400426
553	55.2	5467.7	191.0	-4487.5	-4.630632	137.400238
554	1.2	5468.9	186.2	-4487.4	-4.630652	137.400236
555	46.7	5515.6	179.2	-4484.9	-4.631381	137.400334
559	57.2	5572.8	215.0	-4483.4	-4.632302	137.400525
560	26.3	5599.1	214.5	-4481.5	-4.632410	137.400130
561	30.5	5629.6	195.3	-4481.0	-4.632288	137.399722
562	1.3	5630.9	195.0	-4481.0	-4.632269	137.399728
563	20.2	5651.0	331.4	-4480.4	-4.632515	137.399781
564	41.9	5692.9	179.5	-4483.4	-4.632799	137.399149

565	33.2	5726.1	115.0	-4481.1	-4.633312	137.399040
566	1.4	5727.5	115.0	-4481.1	-4.633321	137.399061
568	68.3	5795.7	333.2	-4480.3	-4.634360	137.399416
569	102.9	5898.7	218.5	-4482.1	-4.634771	137.401049
572	89.4	5988.1	215.4	-4478.5	-4.635697	137.402174
574	38.1	6026.2	170.8	-4478.7	-4.636341	137.402172
581	2.9	6029.1	190.7	-4478.7	-4.636386	137.402182
586	1.6	6030.7	137.2	-4478.6	-4.636361	137.402185
587	22.3	6053.1	91.1	-4477.1	-4.636269	137.402499
588	45.6	6098.6	139.9	-4478.0	-4.636899	137.402792
589	30.0	6128.7	247.8	-4478.5	-4.637361	137.402967
593	31.3	6159.9	177.6	-4479.0	-4.637730	137.402896
595	55.9	6215.8	239.4	-4479.2	-4.638249	137.402664
597	27.5	6243.3	260.8	-4480.3	-4.638223	137.402268
603	50.7	6294.0	206.3	-4480.0	-4.638727	137.402249
606	19.1	6313.1	315.9	-4479.4	-4.638860	137.402033
609	4.8	6318.0	304.8	-4479.2	-4.638843	137.402010
630	24.5	6342.5	119.5	-4479.7	-4.639160	137.402201
631	26.6	6369.1	241.9	-4478.4	-4.639572	137.402145
634	68.7	6437.8	144.2	-4475.4	-4.640390	137.401362
635	75.1	6512.9	190.3	-4475.9	-4.640521	137.400151
636	59.3	6572.2	269.9	-4474.4	-4.640005	137.399300
637	40.4	6612.7	236.0	-4475.0	-4.640230	137.398761
640	1.3	6613.9	217.6	-4475.0	-4.640230	137.398761
641	40.3	6654.3	269.0	-4475.0	-4.639947	137.398279
643	85.2	6739.5	251.0	-4478.6	-4.640294	137.396916
644	104.2	6843.7	179.6	-4475.9	-4.640884	137.395383
646	30.5	6874.2	301.2	-4473.4	-4.640664	137.394932
649	57.2	6931.4	150.1	-4475.0	-4.640434	137.394303
651	31.8	6963.3	180.5	-4473.8	-4.640872	137.394031
655	85.5	7048.8	249.9	-4473.0	-4.641378	137.392712
656	45.7	7094.5	229.5	-4473.5	-4.641963	137.392235
657	121.0	7215.5	230.1	-4473.1	-4.642161	137.390339
658	34.1	7249.6	150.4	-4473.7	-4.642553	137.389964
660	1.2	7250.8	150.7	-4473.7	-4.642553	137.389964
661	137.6	7388.4	255.6	-4474.4	-4.643583	137.388428
662	133.0	7521.3	214.3	-4470.2	-4.645296	137.387226
663	25.3	7546.6	119.7	-4468.5	-4.645663	137.387052
664	66.5	7613.1	160.6	-4468.5	-4.646649	137.386574
665	142.5	7755.6	179.5	-4470.7	-4.647737	137.384829
667	1.2	7756.8	179.9	-4470.7	-4.647737	137.384829
668	105.2	7862.0	230.2	-4471.2	-4.649367	137.384680
669	38.8	7900.8	180.3	-4467.9	-4.649497	137.384049
670	107.1	8008.0	199.9	-4469.8	-4.650872	137.384859
671	116.8	8124.7	79.9	-4472.3	-4.652372	137.383811
672	82.2	8206.9	352.8	-4473.9	-4.653536	137.384183
674	5.3	8212.2	89.2	-4473.8	-4.653448	137.384171
676	16.4	8228.6	69.9	-4473.5	-4.653496	137.383899
677	19.6	8248.2	153.9	-4472.3	-4.653823	137.383900
678	66.5	8314.7	90.3	-4471.5	-4.654926	137.384049
679	1.2	8315.9	89.5	-4471.6	-4.654926	137.384070
683	52.4	8368.3	220.3	-4470.5	-4.655472	137.384641
685	60.4	8428.7	187.2	-4467.2	-4.656308	137.384236
688	82.4	8511.1	210.5	-4464.5	-4.657405	137.383456
689	9.6	8520.7	181.1	-4463.5	-4.657560	137.383456
690	29.5	8550.3	164.0	-4461.9	-4.658036	137.383611

691	23.9	8574.2	242.1	-4460.5	-4.658331	137.383454
692	31.0	8605.2	186.0	-4459.6	-4.658484	137.383046
695	23.4	8628.5	179.9	-4459.3	-4.658824	137.382975
696	19.9	8648.4	169.7	-4457.5	-4.659146	137.383003
702	13.2	8661.6	180.0	-4456.7	-4.659361	137.383015
703	29.6	8691.2	151.4	-4456.7	-4.659819	137.382829
705	4.9	8696.1	115.0	-4456.3	-4.659866	137.382845
706	22.3	8718.4	204.5	-4458.0	-4.660075	137.382667
708	1.2	8719.5	204.2	-4458.0	-4.660075	137.382667
709	14.3	8733.9	344.9	-4458.4	-4.660284	137.382705
710	7.8	8741.7	351.1	-4458.3	-4.660157	137.382668
711	1.9	8743.6	72.2	-4458.2	-4.660138	137.382663
713	8.8	8752.4	353.1	-4457.7	-4.660020	137.382694
714	37.5	8790.0	190.2	-4457.1	-4.659548	137.382393
717	32.2	8822.1	196.7	-4457.0	-4.659935	137.382735
719	3.6	8825.7	233.8	-4457.0	-4.659959	137.382750
729	64.1	8889.8	178.4	-4457.8	-4.659190	137.382105
731	19.8	8909.6	109.6	-4457.9	-4.659285	137.381843
733	58.7	8968.3	274.4	-4457.9	-4.659364	137.380941
735	38.0	9006.3	159.4	-4458.0	-4.658964	137.380553
738	33.0	9039.3	247.0	-4458.1	-4.659144	137.380101
739	23.0	9062.3	247.8	-4458.5	-4.659233	137.379735
740	11.7	9074.0	249.9	-4458.8	-4.659319	137.379596
743	92.6	9166.6	255.6	-4459.6	-4.659342	137.378166
744	31.9	9198.6	238.5	-4459.1	-4.659468	137.377644
746	8.1	9206.7	226.6	-4458.8	-4.659534	137.377528
747	92.1	9298.8	201.9	-4460.9	-4.660861	137.377076
748	22.6	9321.4	199.4	-4459.5	-4.661054	137.376779
751	114.4	9435.7	142.7	-4459.4	-4.662413	137.377245
753	22.4	9458.1	160.0	-4460.6	-4.662699	137.377464
780	21.9	9480.0	105.1	-4459.0	-4.662973	137.377554
785	6.0	9486.0	81.2	-4458.8	-4.662949	137.377623
787	18.5	9504.5	101.2	-4457.8	-4.663130	137.377802
790	7.3	9511.8	139.7	-4457.2	-4.663194	137.377895
792	15.8	9527.6	167.0	-4455.5	-4.663362	137.377782
794	29.9	9557.5	131.4	-4453.8	-4.663523	137.377504
797	53.1	9610.6	30.5	-4460.7	-4.662649	137.377449
799	10.9	9621.5	111.2	-4461.3	-4.662574	137.377518
803	9.9	9631.4	200.8	-4460.9	-4.662660	137.377485
807	16.9	9648.3	84.4	-4459.5	-4.662886	137.377449
812	12.1	9660.3	99.7	-4458.6	-4.662950	137.377635
817	35.6	9695.9	178.3	-4456.9	-4.663224	137.377876
826	26.0	9721.9	175.1	-4455.2	-4.663379	137.377782
835	31.6	9753.4	135.8	-4453.7	-4.663517	137.377445
837	5.2	9758.6	131.4	-4452.6	-4.663582	137.377499
862	69.0	9827.6	45.5	-4459.8	-4.662841	137.377382
864	4.4	9832.0	114.7	-4459.7	-4.662864	137.377450
896	17.1	9849.1	170.2	-4458.5	-4.663079	137.377433
901	27.5	9876.6	196.6	-4453.8	-4.663513	137.377488
903	6.5	9883.1	199.8	-4453.5	-4.663527	137.377452
923	10.2	9893.3	234.9	-4452.1	-4.663594	137.377313
924	9.0	9902.3	141.9	-4451.4	-4.663694	137.377288
926	5.6	9907.9	187.7	-4451.0	-4.663752	137.377347
939	0.6	9908.5	187.4	-4451.0	-4.663752	137.377347
940	13.8	9922.3	183.9	-4450.4	-4.663883	137.377288
944	13.0	9935.3	167.3	-4451.0	-4.663761	137.377328

949	23.1	9958.4	211.8	-4450.2	-4.663988	137.377203
950	37.1	9995.5	205.3	-4449.9	-4.664403	137.376782
951	18.4	10013.9	226.9	-4450.6	-4.664607	137.376550
952	89.6	10103.5	219.3	-4452.4	-4.665341	137.375427
956	64.8	10168.3	273.3	-4452.2	-4.666001	137.374831
957	63.6	10231.9	260.6	-4454.7	-4.666047	137.373767
958	21.9	10253.8	242.6	-4453.9	-4.665962	137.373533
960	102.4	10356.2	230.3	-4456.6	-4.666651	137.372081
962	1.2	10357.4	221.7	-4456.5	-4.666666	137.372067
963	17.3	10374.7	232.3	-4456.1	-4.666799	137.371819
964	32.1	10406.8	281.9	-4455.4	-4.666799	137.371331
967	89.3	10496.1	226.9	-4457.1	-4.667232	137.370245
971	5.9	10502.1	217.6	-4456.2	-4.667310	137.370189
976	74.3	10576.3	134.9	-4454.9	-4.667549	137.371267
978	1.9	10578.2	153.2	-4454.7	-4.667573	137.371281
981	27.9	10606.1	160.1	-4452.0	-4.667843	137.371360
983	20.8	10626.9	204.0	-4450.3	-4.668170	137.371342
984	18.0	10644.9	197.8	-4449.1	-4.668255	137.371271
986	8.9	10653.8	158.2	-4449.7	-4.668114	137.371261
987	42.9	10696.6	119.9	-4454.9	-4.667553	137.371194
990	52.5	10749.1	215.2	-4453.6	-4.667567	137.370323
991	24.4	10773.6	204.5	-4447.9	-4.667875	137.370243
992	6.2	10779.8	216.1	-4447.0	-4.667950	137.370177
995	33.7	10813.5	175.6	-4447.1	-4.668250	137.370016
997	2.7	10816.2	140.5	-4446.7	-4.668293	137.370020
1030	4.5	10820.7	130.9	-4446.9	-4.668276	137.370025
1035	35.1	10855.8	56.9	-4447.0	-4.667958	137.370161
1037	5.5	10861.4	16.3	-4447.5	-4.667884	137.370213
1039	0.3	10861.7	16.1	-4447.5	-4.667889	137.370212
1042	16.9	10878.6	231.8	-4448.0	-4.667942	137.369987
1044	8.6	10887.2	155.0	-4447.9	-4.668048	137.369953
1046	1.2	10888.4	155.2	-4448.0	-4.668066	137.369963
1049	21.0	10909.5	91.4	-4447.0	-4.667994	137.370159
1051	6.0	10915.5	85.2	-4446.8	-4.667984	137.370240
1053	4.4	10919.9	91.0	-4446.7	-4.668035	137.370292
1056	0.9	10920.8	33.4	-4446.8	-4.668025	137.370299
1066	25.3	10946.2	210.7	-4447.8	-4.667994	137.369910
1067	18.3	10964.5	126.0	-4447.1	-4.668263	137.370031
1072	35.2	10999.8	211.2	-4445.0	-4.668682	137.369616
1073	47.1	11046.8	164.7	-4442.5	-4.669375	137.369373
1074	21.2	11068.0	183.4	-4442.0	-4.669720	137.369292
1078	28.7	11096.7	185.2	-4439.8	-4.670183	137.369158
1080	27.0	11123.7	181.6	-4438.1	-4.670639	137.369126
1083	21.6	11145.2	163.7	-4438.6	-4.670968	137.369164
1085	39.0	11184.2	101.0	-4438.1	-4.671414	137.369401
1087	6.2	11190.4	142.4	-4437.7	-4.671475	137.369400
1093	15.8	11206.3	83.1	-4436.8	-4.671421	137.369600
1094	25.5	11231.8	179.0	-4434.5	-4.671650	137.369875
1098	13.0	11244.9	181.4	-4434.0	-4.671826	137.369825
1099	34.4	11279.3	221.5	-4433.6	-4.672323	137.369551
1100	34.5	11313.8	219.4	-4433.0	-4.672832	137.369321
1104	20.9	11334.7	197.8	-4434.3	-4.672928	137.369131
1106	10.5	11345.2	231.2	-4434.1	-4.672960	137.368998
1107	17.0	11362.2	230.1	-4435.2	-4.672854	137.368915
1108	4.8	11367.0	332.5	-4435.4	-4.672847	137.368862
1110	15.2	11382.2	45.7	-4435.4	-4.673003	137.368660

1112	13.9	11396.0	142.1	-4434.7	-4.673136	137.368583
1127	7.6	11403.7	184.2	-4434.5	-4.673146	137.368647
1144	18.6	11422.3	215.2	-4434.0	-4.673099	137.368855
1148	32.1	11454.4	215.6	-4433.4	-4.673327	137.368412
1151	8.9	11463.3	140.5	-4433.1	-4.673389	137.368529
1153	53.7	11517.0	211.0	-4434.4	-4.673886	137.368252
1155	39.3	11556.3	226.9	-4433.2	-4.674395	137.367837
1158	68.4	11624.7	225.2	-4432.2	-4.675198	137.367116
1160	55.9	11680.6	213.2	-4433.8	-4.675794	137.366429
1162	38.4	11718.9	314.2	-4432.3	-4.676310	137.366053
1167	39.4	11758.4	193.1	-4429.3	-4.676959	137.366108
1168	36.7	11795.0	75.7	-4427.4	-4.677560	137.366000
1172	51.3	11846.4	130.7	-4425.7	-4.678290	137.366245
1173	45.7	11892.0	185.2	-4424.9	-4.679022	137.366059
1174	27.8	11919.9	176.5	-4424.9	-4.679218	137.365724
1179	1.2	11921.0	175.8	-4424.8	-4.679238	137.365726
1181	8.6	11929.6	177.9	-4424.6	-4.679323	137.365756
1183	8.9	11938.6	221.7	-4424.0	-4.679363	137.365626
1185	34.6	11973.2	119.5	-4422.9	-4.679435	137.366156
1187	28.9	12002.1	91.1	-4422.9	-4.679281	137.366608
1192	42.1	12044.2	152.5	-4421.9	-4.679588	137.367168
1194	47.4	12091.6	329.2	-4419.6	-4.680219	137.367546
1196	33.3	12124.9	235.5	-4419.9	-4.680045	137.368018
1215	68.3	12193.2	330.4	-4421.9	-4.679673	137.367213
1216	42.9	12236.1	345.6	-4424.2	-4.679053	137.367034
1221	9.6	12245.7	340.0	-4423.8	-4.678995	137.367096
1243	12.7	12258.4	231.6	-4423.5	-4.679064	137.367114
1244	2.1	12260.5	239.8	-4423.4	-4.679074	137.367085
1248	45.7	12306.2	209.6	-4423.2	-4.679368	137.366381
1249	70.6	12376.8	327.3	-4426.7	-4.678745	137.365536
1250	17.0	12393.8	334.6	-4426.5	-4.678760	137.365580
1255	14.7	12408.5	305.1	-4427.1	-4.678544	137.365453
1256	24.9	12433.4	297.9	-4427.6	-4.678302	137.365110
1257	0.0	12433.4	199.4	-4427.6	-4.678302	137.365110
1260	6.9	12440.4	239.9	-4428.0	-4.678194	137.365072
1262	70.0	12510.4	326.7	-4430.1	-4.677623	137.364110
1264	24.6	12535.0	271.6	-4432.1	-4.677569	137.363716
1267	24.3	12559.3	239.2	-4433.5	-4.677754	137.363353
1269	36.3	12595.6	217.1	-4432.6	-4.678125	137.362943
1274	46.1	12641.7	211.3	-4427.4	-4.678607	137.362474
1276	12.2	12653.9	339.2	-4426.6	-4.678625	137.362358
1281	13.5	12667.4	320.1	-4426.4	-4.678448	137.362218
1282	26.8	12694.1	214.4	-4426.5	-4.678445	137.361890
1283	39.3	12733.4	234.5	-4427.3	-4.678543	137.361240
1284	28.4	12761.9	209.8	-4427.1	-4.678627	137.360840
1289	14.6	12776.4	135.2	-4426.9	-4.678425	137.360712
1290	22.5	12799.0	138.7	-4426.7	-4.678254	137.360375
1292	7.0	12806.0	247.2	-4427.0	-4.678186	137.360323
1294	16.7	12822.7	250.4	-4427.9	-4.678222	137.360066
1296	4.0	12826.7	290.8	-4427.8	-4.678202	137.360004
1298	38.4	12865.0	235.2	-4429.7	-4.678310	137.359418
1301	4.7	12869.7	328.2	-4429.4	-4.678359	137.359359
1309	7.3	12877.1	279.9	-4429.1	-4.678285	137.359273
1310	20.7	12897.7	256.8	-4431.0	-4.678339	137.358931
1311	16.6	12914.3	217.5	-4431.2	-4.678201	137.358930
1315	1.2	12915.5	215.4	-4431.3	-4.678217	137.358918

1316	25.5	12940.9	355.9	-4429.6	-4.678316	137.359160
1317	7.3	12948.2	230.2	-4429.0	-4.678261	137.359234
1329	13.0	12961.2	58.4	-4429.3	-4.678240	137.359173
1342	5.8	12967.0	182.5	-4429.3	-4.678196	137.359202
1344	28.2	12995.2	331.1	-4431.3	-4.678277	137.358886
1346	16.5	13011.8	304.7	-4433.3	-4.678178	137.358756
1349	12.0	13023.8	284.4	-4434.5	-4.678254	137.358650
1352	16.5	13040.3	235.5	-4434.9	-4.678327	137.358531
1353	51.9	13092.2	240.6	-4434.9	-4.678670	137.357823
1357	9.1	13101.4	9.2	-4435.6	-4.678670	137.357706
1369	31.9	13133.3	191.1	-4433.3	-4.679170	137.357681
1371	32.2	13165.5	163.8	-4430.3	-4.679605	137.357965
1373	31.6	13197.1	163.6	-4429.5	-4.680071	137.358158
1376	17.4	13214.5	178.3	-4429.4	-4.680366	137.358188
1378	44.2	13258.7	159.4	-4428.7	-4.681072	137.358073
1383	31.3	13290.0	194.8	-4428.0	-4.681571	137.357943
1384	64.6	13354.6	203.1	-4426.4	-4.682396	137.357345
1385	68.4	13423.0	164.4	-4421.7	-4.683500	137.357494
1386	1.2	13424.2	167.1	-4421.7	-4.683521	137.357499
1387	12.3	13436.5	186.4	-4420.6	-4.683733	137.357477
1398	65.8	13502.3	206.8	-4419.4	-4.684237	137.356675
1399	31.9	13534.2	240.2	-4420.6	-4.684578	137.356275
1400	13.5	13547.8	218.3	-4420.3	-4.684720	137.356182
1401	26.5	13574.2	335.3	-4417.3	-4.685133	137.356131
1405	29.7	13603.9	318.0	-4417.9	-4.685180	137.355671
1410	67.9	13671.8	189.6	-4415.7	-4.685962	137.355070
1412	45.3	13717.1	198.4	-4412.2	-4.686642	137.354730
1414	44.8	13761.9	152.7	-4410.8	-4.687163	137.354390
1417	14.6	13776.5	331.4	-4410.4	-4.687185	137.354474
1427	11.1	13787.6	185.0	-4409.6	-4.687369	137.354450
1428	45.6	13833.1	231.7	-4407.7	-4.687932	137.354027
1431	53.0	13886.1	192.6	-4405.4	-4.688671	137.353581
1432	51.5	13937.7	170.7	-4403.9	-4.689277	137.353432
1433	54.7	13992.3	179.5	-4401.3	-4.690159	137.353373
1434	1.2	13993.5	178.8			
1435	63.4	14056.9	209.0	-4398.6	-4.691072	137.352835
1438	41.5	14098.4	149.3	-4397.1	-4.691746	137.352761
1439	34.0	14132.4	150.0	-4393.8	-4.692201	137.353103
1446	53.1	14185.6	196.2	-4388.8	-4.693054	137.353000
1448	78.0	14263.5	154.3	-4383.8	-4.694173	137.353128
1452	44.5	14308.1	155.0	-4378.8	-4.694773	137.353419
1454	40.5	14348.6	215.7	-4380.1	-4.695388	137.353138
1455	27.5	14376.1	287.9	-4379.5	-4.695202	137.353006
1468	87.8	14463.9	100.7	-4375.8	-4.696268	137.353717
1469	34.8	14498.7	139.2	-4374.4	-4.696646	137.354129
1471	41.8	14540.5	142.5	-4372.7	-4.697072	137.354563
1473	16.4	14556.9	142.0	-4371.2	-4.697312	137.354695
1475	16.2	14573.0	192.3	-4370.1	-4.697568	137.354597
1478	12.7	14585.7	179.6	-4368.8	-4.697779	137.354607
1480	34.9	14620.6	178.8	-4367.1	-4.698369	137.354630
1482	39.3	14659.8	150.1	-4364.3	-4.698971	137.354891
1485	35.9	14695.7	161.0	-4361.3	-4.699516	137.355171
1487	17.4	14713.1	243.0	-4360.5	-4.699647	137.354906
1489	7.1	14720.2	277.5	-4360.8	-4.699603	137.354949
1499	11.4	14731.6	212.5	-4359.8	-4.699775	137.354873
1500	31.3	14762.9	98.6	-4359.8	-4.700149	137.355090

1501	46.0	14808.9	150.5	-4354.9	-4.700830	137.355413
1502	25.5	14834.4	134.0	-4353.8	-4.700985	137.355798
1503	37.1	14871.5	156.4	-4351.8	-4.701562	137.356046
1506	50.8	14922.3	184.9	-4348.6	-4.702422	137.356025
1507	29.4	14951.8	220.2	-4349.5	-4.702815	137.355825
1508	44.3	14996.1	212.8	-4351.0	-4.703451	137.355497
1509	40.1	15036.2	181.6	-4349.8	-4.704095	137.355551
1512	44.0	15080.2	161.2	-4346.5	-4.704769	137.355754
1514	43.5	15123.7	159.9	-4343.4	-4.705312	137.356177
1516	24.3	15148.0	137.8	-4341.6	-4.705692	137.356162
1519	36.8	15184.8	79.1	-4337.9	-4.706139	137.356473
1521	11.4	15196.2	110.0	-4336.4	-4.706176	137.356653
1526	15.9	15212.1	95.7	-4335.8	-4.706323	137.356701
1553	17.7	15229.8	238.6	-4335.8	-4.706201	137.356727
1555	0.0	15229.8	243.1	-4335.8	-4.706200	137.356728
1571	16.9	15246.7	130.2	-4335.0	-4.706375	137.356950
1574	26.0	15272.7	136.3	-4333.5	-4.706670	137.357275
1576	29.6	15302.3	130.3	-4331.3	-4.707039	137.357608
1577	46.1	15348.4	139.6	-4327.4	-4.707671	137.358014
1578	25.4	15373.8	124.9	-4325.1	-4.707794	137.358418
1582	44.4	15418.2	154.6	-4321.5	-4.708481	137.358663
1583	16.3	15434.5	200.6	-4321.2	-4.708713	137.358525
1584	31.5	15465.9	164.9	-4318.6	-4.709222	137.358631
1585	13.6	15479.6	189.4	-4317.5	-4.709448	137.358621
1587	28.2	15507.7	178.1	-4314.4	-4.709901	137.358709
1589	31.0	15538.7	172.7	-4311.5	-4.710381	137.358625
1591	8.3	15547.0	178.9	-4311.3	-4.710432	137.358567
1594	26.2	15573.2	248.7	-4310.4	-4.710716	137.358238
1596	20.6	15593.8	250.2	-4311.2	-4.710789	137.357896
1598	28.6	15622.3	237.6	-4312.5	-4.711008	137.357472
1601	26.6	15648.9	184.8	-4314.2	-4.711185	137.357186
1604	55.4	15704.3	23.7	-4311.5	-4.710725	137.357893
1605	21.7	15726.0	351.5	-4313.5	-4.710364	137.357821
1608	9.3	15735.3	257.0	-4313.4	-4.710330	137.357716
1610	26.4	15761.7	173.2	-4312.1	-4.710667	137.357869
1611	23.5	15785.2	134.6	-4309.9	-4.710951	137.358143
1612	31.9	15817.1	111.6	-4306.9	-4.711296	137.358524
1616	0.2	15817.4	114.4	-4306.9	-4.711295	137.358520
1617	20.0	15837.3	268.0	-4306.6	-4.711431	137.358536
1628	25.2	15862.5	122.9	-4305.8	-4.711659	137.358878
1630	40.8	15903.3	138.2	-4304.6	-4.712144	137.359345
1632	29.2	15932.5	207.7	-4304.1	-4.712572	137.359197
1635	28.6	15961.1	179.5	-4304.1	-4.712916	137.358904
1636	6.5	15967.6	268.7	-4304.4	-4.712933	137.358832
1639	31.2	15998.8	149.2	-4302.3	-4.713247	137.359220
1641	1.2	16000.0	149.3			
1642	27.0	16027.0	92.0	-4301.0	-4.713716	137.359246
1643	40.9	16067.9	69.6	-4300.3	-4.713544	137.359843
1645	20.7	16088.6	70.5	-4300.0	-4.713385	137.360153
1646	30.7	16119.4	92.0	-4300.0	-4.713294	137.360651
1648	9.5	16128.9	79.0	-4300.0	-4.713292	137.360774
1659	34.7	16163.6	225.0	-4299.8	-4.713454	137.360358
1662	24.6	16188.2	194.0	-4298.7	-4.713832	137.360206
1664	1.8	16190.0	165.9	-4298.6	-4.713857	137.360213
1666	12.9	16202.9	177.8	-4298.1	-4.714011	137.360207
1669	34.2	16237.1	132.0	-4296.4	-4.714411	137.360472

1671	4.4	16241.5	172.6	-4295.9	-4.714476	137.360498
1672	23.4	16264.9	195.0	-4295.1	-4.714859	137.360483
1673	8.7	16273.5	198.6	-4294.6	-4.715000	137.360439
1676	17.3	16290.8	154.0	-4293.8	-4.715278	137.360509
1677	33.1	16323.9	157.5	-4291.6	-4.715793	137.360731
1678	28.7	16352.6	139.2	-4289.7	-4.716056	137.360995
1679	30.3	16382.9	148.5	-4287.7	-4.716453	137.361298
1680	28.5	16411.4	139.3	-4286.2	-4.716865	137.361536
1682	1.2	16412.6	139.2			
1683	28.9	16441.5	113.4	-4284.6	-4.717228	137.361730
1684	37.6	16479.1	125.9	-4282.5	-4.717673	137.362007
1685	18.3	16497.4	139.4	-4280.9	-4.717866	137.362248
1686	13.1	16510.5	135.8	-4280.5	-4.718007	137.362262
1689	12.3	16522.9	147.9	-4279.8	-4.718194	137.362252
1691	7.8	16530.6	211.7	-4279.4	-4.718292	137.362215
1693	18.4	16549.0	144.0	-4279.2	-4.718300	137.362392
1696	53.3	16602.4	128.1	-4275.6	-4.718407	137.363151
1698	47.9	16650.2	92.2	-4272.6	-4.718872	137.363767
1700	33.3	16683.5	141.9	-4270.4	-4.719019	137.364250
1703	2.9	16686.4	144.5	-4269.9	-4.719058	137.364277
1705	14.9	16701.3	107.5	-4269.0	-4.719160	137.364342
1707	16.4	16717.6	154.1	-4267.6	-4.719392	137.364335
1711	13.6	16731.2	146.8	-4266.6	-4.719536	137.364429
1712	7.7	16739.0	184.8	-4265.5	-4.719654	137.364449
1717	28.5	16767.4	134.5	-4262.5	-4.720094	137.364428
1718	26.1	16793.5	116.0	-4260.2	-4.720235	137.364837
1719	15.6	16809.1	235.4	-4260.2	-4.720341	137.364617
1720	26.3	16835.4	135.0	-4257.3	-4.720706	137.364639
1721	12.4	16847.9	134.9	-4255.6	-4.720799	137.364765
1724	27.1	16874.9	115.3	-4254.0	-4.720888	137.365202
1726	21.5	16896.4	127.3	-4251.7	-4.721069	137.365512
1727	27.3	16923.8	109.5	-4248.7	-4.721238	137.365942
1728	19.9	16943.6	62.3	-4247.8	-4.721185	137.366263
1730	33.0	16976.6	64.3	-4247.3	-4.720891	137.366705
1732	14.9	16991.5	80.6	-4246.2	-4.720941	137.366921
1734	16.6	17008.1	94.3	-4246.4	-4.720813	137.367164
1737	20.7	17028.8	96.0	-4245.1	-4.720790	137.367506
1739	33.0	17061.8	103.8	-4243.8	-4.720662	137.368006
1741	15.9	17077.6	99.4	-4243.5	-4.720600	137.368194
1746	26.5	17104.2	117.3	-4240.7	-4.720669	137.368559
1747	10.1	17114.2	113.7	-4239.3	-4.720740	137.368714
1748	9.0	17123.3	171.1	-4239.0	-4.720813	137.368772
1751	8.2	17131.5	81.9	-4238.5	-4.720753	137.368845
1752	10.3	17141.8	74.1	-4237.6	-4.720731	137.369016
1753	26.5	17168.3	104.1	-4236.2	-4.720838	137.369438
1754	37.9	17206.2	179.1	-4233.0	-4.721167	137.369881
1781	36.6	17242.7	-179.7	-4227.3	-4.721671	137.370068
1782	12.8	17255.5	131.8	-4225.5	-4.721803	137.370226
1785	32.5	17288.0	52.1	-4225.9	-4.721768	137.370736
1786	15.9	17303.8	47.0	-4227.9	-4.721558	137.370903
1787	13.8	17317.6	44.6	-4228.9	-4.721390	137.371060
1788	25.0	17342.6	122.3	-4227.5	-4.721511	137.371384
1789	20.3	17363.0	179.4	-4225.1	-4.721724	137.371620
1793	23.6	17386.5	108.1	-4224.5	-4.721793	137.371969
1794	23.1	17409.6	98.8	-4222.5	-4.721947	137.372307
1795	30.3	17439.9	99.4	-4220.1	-4.722115	137.372712

1796	19.7	17459.6	-179.1	-4217.5	-4.722351	137.372879
1798	1.1	17460.9	177.8			
1799	22.4	17483.4	-144.5	-4214.2	-4.722648	137.372777
1800	28.3	17511.7	-156.5	-4209.1	-4.723077	137.372674
1802	14.5	17526.2	-121.0	-4206.9	-4.723229	137.372530
1807	28.4	17554.6	163.5	-4202.6	-4.723537	137.372746
1809	9.1	17563.7	157.1	-4200.1	-4.723678	137.372752
1812	5.8	17569.5	124.2	-4198.9	-4.723717	137.372832
1814	12.1	17581.6	-160.8	-4196.6	-4.723872	137.372863
1819	18.0	17599.6	-134.0	-4195.4	-4.724105	137.372669
1822	41.2	17640.7	-155.7	-4191.6	-4.724744	137.372494
1827	9.4	17650.2	140.6	-4190.8	-4.724866	137.372593
1828	30.4	17680.6	137.1	-4186.7	-4.725301	137.372861
1829	39.1	17719.7	90.5	-4187.6	-4.724985	137.373335
1830	11.2	17730.8	121.6	-4185.1	-4.725077	137.373495
1834	14.3	17745.1	179.7	-4182.3	-4.725270	137.373617
1837	29.2	17774.3	-179.9	-4180.5	-4.725487	137.374050
1843	0.0	17774.4	161.5	-4180.4	-4.725487	137.374050
1846	22.1	17796.5	147.7	-4177.3	-4.725801	137.374236
1848	14.9	17811.3	-157.5	-4176.7	-4.726014	137.374116
1850	20.8	17832.1	-177.0	-4174.1	-4.726363	137.374086
1864	25.3	17857.4	136.7	-4174.7	-4.726458	137.373672
1867	22.2	17879.6	128.4	-4172.2	-4.726644	137.373991
1869	18.2	17897.9	131.8	-4170.5	-4.726766	137.374257
1871	10.7	17908.6	120.5	-4168.5	-4.726887	137.374382
1873	0.4	17909.0	132.3	-4168.3	-4.726891	137.374390
1877	26.9	17935.9	-147.4	-4166.6	-4.727165	137.374183
1887	26.7	17962.6	169.0	-4163.7	-4.727595	137.374229
1889	35.7	17998.4	146.2	-4160.1	-4.728133	137.374437
1891	14.1	18012.5	-140.2	-4158.0	-4.728315	137.374578
1894	19.7	18032.1	162.2	-4157.2	-4.728287	137.374879
1896	25.3	18057.5	122.7	-4157.0	-4.728258	137.375304
1901	27.6	18085.1	-162.8	-4154.2	-4.728672	137.375479
1903	11.0	18096.0	-163.2	-4154.6	-4.728659	137.375406
1905	14.3	18110.4	120.9	-4152.9	-4.728628	137.375638
1910	19.1	18129.5	173.1	-4155.0	-4.728596	137.375322
1912	5.5	18135.1	143.3	-4154.6	-4.728669	137.375377
1923	11.0	18146.1	143.6	-4153.6	-4.728728	137.375542
1928	38.4	18184.5	-175.1	-4150.1	-4.728985	137.375962
1930	31.8	18216.3	-171.2	-4149.3	-4.729290	137.376080
1939	5.4	18221.7	-176.8	-4148.7	-4.729378	137.376072
1942	29.5	18251.3	-15.7	-4148.0	-4.729596	137.376384
1944	16.4	18267.6	-137.8	-4147.5	-4.729821	137.376284
1946	44.6	18312.3	-132.7	-4148.2	-4.729268	137.376800
1949	20.2	18332.5	179.6	-4146.7	-4.729128	137.377104
1950	23.8	18356.3	130.7	-4146.4	-4.728805	137.377209
1962	52.3	18408.6	-141.8	-4146.8	-4.728507	137.377606
1985	28.5	18437.0	-159.3	-4147.3	-4.728059	137.377785
1986	81.9	18518.9	175.3	-4150.6	-4.727474	137.378273
1989	45.5	18564.4	149.5	-4152.6	-4.726920	137.378690
1991	15.2	18579.6	175.7	-4152.3	-4.726826	137.378923
1993	34.4	18614.1	149.8	-4154.2	-4.726439	137.379336
1996	39.0	18653.0	-113.7	-4155.4	-4.726000	137.379802
1998	10.7	18663.8	-119.1	-4157.0	-4.725828	137.379853
1999	15.7	18679.5	140.5	-4159.7	-4.725591	137.379957
2003	0.1	18679.6	-154.3	-4159.7	-4.725589	137.379956

2004	58.4	18738.0	-148.9	-4164.6	-4.724768	137.380363
2007	35.4	18773.4	157.1	-4161.0	-4.725043	137.380821
2009	47.1	18820.5	148.4	-4157.1	-4.724951	137.381588
2012	38.4	18858.9	-155.5	-4155.4	-4.725289	137.381546
2014	18.1	18877.0	84.3	-4152.7	-4.725489	137.381649
2017	7.6	18884.6	-117.8	-4152.8	-4.725463	137.381729
2020	14.0	18898.7	-116.1	-4154.1	-4.725394	137.381563
2023	13.5	18912.1	156.9	-4156.3	-4.725196	137.381495
2027	88.2	19000.3	-104.5	-4165.9	-4.724633	137.380167
2030	16.1	19016.4	-114.7	-4166.3	-4.724681	137.379941
2032	18.4	19034.7	-115.5	-4168.2	-4.724634	137.379658
2034	28.8	19063.5	168.3	-4176.0	-4.724193	137.379505
2036	30.2	19093.7	156.1	-4181.3	-4.723921	137.379133
2039	44.3	19138.0	-143.9	-4179.1	-4.724447	137.378631
2040	42.6	19180.7	-104.8	-4178.6	-4.724776	137.378004
2041	13.8	19194.5	-108.4	-4179.2	-4.724768	137.377773
2044	21.3	19215.8	-103.6	-4180.6	-4.724832	137.377420
2045	38.0	19253.8	-113.4	-4187.4	-4.724478	137.377380
2047	13.0	19266.9	-160.2	-4188.7	-4.724399	137.377382
2051	0.6	19267.5	140.9	-4188.8	-4.724389	137.377382
2052	9.8	19277.2	-120.4	-4191.3	-4.724254	137.377292
2053	8.1	19285.3	-158.9	-4192.5	-4.724196	137.377247
2086	5.6	19290.9	164.4	-4192.0	-4.724210	137.377281
2087	0.0	19290.9	163.7	-4192.0	-4.724210	137.377281
2089	14.2	19305.1	167.0	-4192.4	-4.724226	137.377172
2092	29.1	19334.2	-163.0	-4186.3	-4.724546	137.377398
2094	51.9	19386.0	136.5	-4176.8	-4.725102	137.377589
2095	38.1	19424.1	131.9	-4170.9	-4.725512	137.378038
2098	30.9	19455.0	171.9	-4164.8	-4.725694	137.378503
2102	22.6	19477.6	15.7	-4162.8	-4.725690	137.378776
2104	57.7	19535.3	10.3	-4165.6	-4.724997	137.379358
2107	58.9	19594.1	10.0	-4163.8	-4.724945	137.380316
2108	6.7	19600.8	2.2	-4164.4	-4.724835	137.380337
2115	1.3	19602.1	-153.5	-4164.5	-4.724815	137.380337
2116	54.2	19656.3	-177.3	-4159.0	-4.725571	137.380190
2119	22.1	19678.4	25.9	-4158.7	-4.725657	137.379975
2120	14.6	19693.0	-9.6	-4158.6	-4.725662	137.380008
2126	74.8	19767.8	-89.3	-4162.6	-4.725677	137.378846
2128	54.6	19822.4	-41.9	-4169.0	-4.725651	137.378088
2132	3.9	19826.3	-24.6	-4169.9	-4.725593	137.378061
2156	40.6	19866.9	151.3	-4162.1	-4.726099	137.378477
2157	44.9	19911.8	-134.5	-4155.6	-4.726822	137.378402
2161	35.5	19947.2	179.9	-4151.6	-4.727345	137.378336
2163	17.5	19964.8	-151.5	-4149.9	-4.727605	137.378325
2166	6.6	19971.3	-52.0	-4150.1	-4.727606	137.378251
2218	5.1	19976.4	-145.2	-4150.7	-4.727554	137.378184
2221	59.4	20035.9	-150.0	-4146.7	-4.728401	137.377640
2222	9.7	20045.5	137.4	-4147.0	-4.728514	137.377571
2250	16.8	20062.3	0.0	-4145.8	-4.728477	137.377757
2252	7.9	20070.3	4.3	-4145.0	-4.728449	137.377835
2255	27.0	20097.3	3.4	-4144.0	-4.728509	137.377966
2256	9.4	20106.7	16.2	-4143.6	-4.728492	137.378124
2257	12.2	20118.9	-138.4	-4143.8	-4.728414	137.378160
2296	1.2	20120.1	-138.2			
2297	36.7	20156.8	112.3	-4142.8	-4.728501	137.378749
2298	31.6	20188.4	134.0	-4141.1	-4.728830	137.379179

2299	30.7	20219.1	125.8	-4139.9	-4.729268	137.379298
2300	21.7	20240.8	9.8	-4140.1	-4.729274	137.379569
2302	31.7	20272.5	145.4	-4142.0	-4.729148	137.379996
2304	6.0	20278.5	168.0	-4141.7	-4.729247	137.380018
2306	8.4	20287.0	113.2	-4141.1	-4.729317	137.380124
2309	20.7	20307.6	64.4	-4143.0	-4.729292	137.380446
2311	40.9	20348.5	57.5	-4145.4	-4.729127	137.381083
2313	38.2	20386.8	2.9	-4147.4	-4.728473	137.381157
2316	26.1	20412.9	15.0	-4148.3	-4.728090	137.381330
2320	36.7	20449.6	52.9	-4150.3	-4.727720	137.381540
2338	31.3	20480.9	19.4	-4150.6	-4.727897	137.381913
2350	5.5	20486.5	93.8	-4150.5	-4.727938	137.381842
2352	30.6	20517.1	4.8	-4150.8	-4.727496	137.382002
2354	25.6	20542.7	0.1	-4152.3	-4.727188	137.382263
2357	34.1	20576.8	-12.4	-4153.9	-4.726991	137.382707
2359	27.7	20604.4	35.6	-4155.2	-4.726605	137.382969
2361	28.0	20632.4	19.1	-4157.2	-4.726357	137.383291
2364	30.2	20662.7	-10.7	-4157.8	-4.726109	137.383663
2365	1.9	20664.6	0.5	-4157.9	-4.726079	137.383653
2381	0.3	20664.9	15.5	-4158.0	-4.726074	137.383655
2407	3.0	20667.9	13.0	-4158.1	-4.726024	137.383665
2408	14.4	20682.3	-32.6	-4158.0	-4.726094	137.383580
2412	42.7	20725.0	130.8	-4158.2	-4.725676	137.384102
2413	9.0	20734.1	-89.1	-4157.7	-4.725819	137.384142
2414	5.9	20739.9	14.5	-4157.9	-4.725723	137.384165
2416	67.1	20807.1	-160.2	-4157.0	-4.726310	137.383264
2420	61.5	20868.6	-65.5	-4153.9	-4.726959	137.382714
2422	7.8	20876.3	-88.1	-4154.1	-4.726929	137.382726
2429	46.8	20923.1	113.8	-4154.2	-4.727085	137.383499
2432	36.1	20959.3	140.4	-4152.4	-4.727260	137.384051
2434	40.8	21000.1	140.3	-4150.0	-4.727808	137.384308
2435	11.0	21011.1	139.5	-4145.7	-4.727930	137.384186
2436	19.2	21030.3	-125.8	-4148.5	-4.728111	137.383924
2439	16.3	21046.6	142.9	-4144.8	-4.728331	137.384067
2447	12.9	21059.5	-139.0	-4147.1	-4.728235	137.383942
2448	32.9	21092.4	170.7	-4145.2	-4.728701	137.383727
2449	11.7	21104.1	-125.1	-4143.9	-4.728855	137.383658
2453	25.5	21129.6	-142.5	-4142.3	-4.729044	137.383392
2454	7.5	21137.2	-149.2	-4142.2	-4.729047	137.383342
2459	2.7	21139.9	144.3	-4141.9	-4.729083	137.383366
2463	5.6	21145.4	-164.5	-4141.3	-4.729125	137.383286
2466	53.5	21199.0	-152.7	-4138.7	-4.729871	137.383535
2468	14.6	21213.6	-98.2	-4138.3	-4.730028	137.383384
2473	21.6	21235.2	-92.1	-4137.0	-4.730096	137.383056
2475	29.0	21264.2	179.6	-4135.5	-4.730212	137.383460
2476	8.8	21273.0	-173.2	-4135.3	-4.730231	137.383428
2477	23.5	21296.5	166.4	-4134.4	-4.730267	137.383685
2480	15.2	21311.7	-116.1	-4133.1	-4.730405	137.383529
2481	6.9	21318.5	-123.5	-4133.4	-4.730309	137.383541
2555	39.8	21358.4	-145.7	-4129.6	-4.730907	137.383501
2556	8.2	21366.6	-124.4	-4129.5	-4.730925	137.383476
2559	25.6	21392.2	175.4	-4126.5	-4.731232	137.383396
2563	4.7	21396.9	-150.6	-4125.8	-4.731299	137.383354
2565	33.9	21430.7	151.4	-4123.8	-4.731805	137.383239
2568	40.6	21471.4	-158.2	-4119.5	-4.732368	137.383448
2570	9.6	21480.9	-147.0	-4118.1	-4.732508	137.383384

2572	4.3	21485.2	-72.5	-4117.5	-4.732566	137.383371
2575	39.2	21524.5	132.5	-4120.9	-4.732323	137.382994
2577	16.5	21541.0	-152.2	-4119.3	-4.732509	137.382827
2582	4.6	21545.5	-173.0	-4118.1	-4.732576	137.382831
2586	42.6	21588.1	-114.5	-4121.5	-4.732500	137.382436
2589	8.6	21596.7	-25.0	-4121.7	-4.732595	137.382358
2590	31.5	21628.2	-122.7	-4124.3	-4.732527	137.382170
2592	1.2	21629.4	-123.8			
2593	36.1	21665.6	165.3	-4123.3	-4.732951	137.381746
2595	34.4	21700.0	-126.8	-4121.7	-4.733336	137.381325
2602	20.3	21720.3	-179.8	-4119.2	-4.733652	137.381425
2604	14.8	21735.1	153.7	-4117.9	-4.733794	137.381579
2606	14.3	21749.4	175.3	-4116.0	-4.734019	137.381613
2609	8.0	21757.4	-144.0	-4114.4	-4.734132	137.381548
2611	35.9	21793.3	-100.3	-4110.7	-4.734070	137.381160
2613	12.9	21806.2	-122.5	-4109.6	-4.734043	137.380990
2616	28.9	21835.1	-35.7	-4109.6	-4.734255	137.380683
2618	23.0	21858.2	-133.3	-4108.6	-4.734583	137.380479
2633	16.3	21874.5	-128.0	-4106.4	-4.734705	137.380243
2639	0.1	21874.6	-128.7	-4106.4	-4.734704	137.380245
2643	42.9	21917.5	122.0	-4109.3	-4.734338	137.380823
2645	28.5	21946.1	-141.1	-4109.5	-4.734745	137.380730
2654	40.6	21986.6	-150.1	-4108.7	-4.735401	137.380635
2657	47.5	22034.1	-160.7	-4104.8	-4.736123	137.380356
2658	21.0	22055.1	170.2	-4101.8	-4.736402	137.380514
2659	21.0	22076.1	117.2	-4098.2	-4.736489	137.380773
2661	12.6	22088.7	-159.4	-4095.4	-4.736616	137.380853
2664	4.7	22093.4	-16.8	-4095.4	-4.736585	137.380899
2691	24.9	22118.3	138.0	-4094.8	-4.736721	137.380753
2692	2.0	22120.3	118.8	-4093.5	-4.736736	137.380778
2693	1.1	22121.4	135.2	-4092.8	-4.736750	137.380788
2695	3.5	22124.9	124.0	-4091.8	-4.736774	137.380832
2698	13.3	22138.1	154.4	-4088.8	-4.736956	137.380950
2700	16.7	22154.8	-151.8	-4088.8	-4.737048	137.380839
2702	4.6	22159.5	144.8	-4088.7	-4.737061	137.380790
2729	5.6	22165.1	122.7	-4089.6	-4.737024	137.380703
2732	24.4	22189.5	-39.4	-4090.9	-4.736821	137.380883
2734	34.3	22223.8	-112.7	-4101.5	-4.736416	137.380550
2742	65.7	22289.5	159.7	-4105.4	-4.735710	137.380695
2745	35.7	22325.1	25.7	-4107.8	-4.735271	137.381058
2747	2.5	22327.6	20.2	-4107.9	-4.735237	137.381058
2780	37.1	22364.7	-100.2	-4107.3	-4.735099	137.381659
2781	58.1	22422.8	-99.5	-4106.6	-4.735159	137.382562
2783	55.1	22477.9	-88.4	-4103.9	-4.734989	137.383416
2786	61.3	22539.2	-100.1	-4105.6	-4.734311	137.384116
2788	86.0	22625.2	-89.6	-4101.6	-4.734080	137.385510
2790	95.9	22721.1	-172.7	-4109.8	-4.732619	137.386130
2793	48.7	22769.8	175.2	-4105.9	-4.732442	137.386911
2795	83.9	22853.8	170.5	-4098.6	-4.733185	137.388016
2797	31.0	22884.8	170.9	-4092.5	-4.733663	137.388191
2800	10.2	22895.1	173.4	-4093.0	-4.733624	137.388197
2802	56.2	22951.3	-140.0	-4099.1	-4.732994	137.388803
2804	15.8	22967.0	-142.6	-4098.8	-4.733033	137.389022
2813	44.4	23011.4	175.4	-4101.7	-4.732501	137.389459
2816	62.9	23074.3	169.9	-4107.9	-4.732016	137.389987
2817	102.5	23176.8	-100.0	-4113.7	-4.731062	137.391268

2820	32.8	23209.6	-100.6	-4117.5	-4.730572	137.391068
2822	1.2	23210.8	-100.3	-4117.4	-4.730576	137.391048
2824	83.8	23294.6	-90.4	-4126.5	-4.729530	137.391150
2829	24.4	23319.0	166.8	-4128.1	-4.729307	137.391138
